# Effects of Lignin-Diverted Reductant with Polyphenol Oxidases on Cellulose Degradation by Wild and Mutant Types of Lytic Polysaccharide Monooxygenase

**DOI:** 10.3390/cimb46040230

**Published:** 2024-04-21

**Authors:** Kai Li, Yuan Wang, Xiao Guo, Bo Wang

**Affiliations:** College of Chemical and Biological Engineering, Shandong University of Science and Technology, Qingdao 266590, China; adolf5259@hotmail.com (K.L.); skdwy1993@163.com (Y.W.); guoxiao920218@163.com (X.G.)

**Keywords:** lytic polysaccharide monooxygenase, polyphenol oxidases, reducing agents, lignin-diverted small phenolic compounds

## Abstract

Establishing a multi-enzyme synergistic lignocellulosic biodegradation system using lytic polysaccharide monooxygenase (LPMO) and polyphenol oxidases is vital for efficiently utilizing plant biomass waste, ultimately benefiting the carbon cycle and promoting environmental protection. Single-residue mutations of LPMO can improve the efficiency of lignocellulosic biomass degradation. However, the activity of mutant-type LPMO in relation to lignin-diverted reducing agents has not been sufficiently explored. In this study, laccase and tyrosinase were initially investigated and their optimal conditions and impressive thermal stability were revealed, indicating their potential synergistic abilities with LPMO in lignocellulose biodegradation. When utilizing gallic acid as a reducing agent, the activities of LPMOs were increased by over 10%, which was particularly evident in mutant-type LPMOs after the addition of polyphenol oxidases. In particular, the combination of tyrosinase with either 4-hydroxy-3-methoxyphenylacetone or *p*-coumaric acid was shown to enhance the efficacy of LPMOs. Furthermore, the highest activity levels of wild-type LPMOs were observed with the addition of laccase and 3-methylcatechol. The similarities between wild and mutant LPMOs regarding their activities in lignin-diverted phenolic compounds and reducing agents are almost identical, suggesting that the single-residue mutation of LPMO does not have a detrimental effect on its performance. Above all, this study indicates that understanding the performance of both wild and mutant types of LPMOs in the presence of polyphenol oxidases and various reducing agents constitutes a key link in the industrialization of the multi-enzyme degradation of lignocellulose.

## 1. Introduction

Lignocellulose is generally recognized as the primary component of plant biomass, and it has been identified as a high-value raw material for advanced biofuel production, bio-based materials, and green chemistry [1,2]. Lignin, in particular, is considered to be the second-most-widespread plant biopolymer on earth, after cellulose, and approximately 70 million tons of lignin is produced annually by the pulping industry [3,4]. Lignin, a highly branched aromatic polymer with extraordinary structural diversity, is regularly polymerized with a series of oxidative radical coupling reactions that occur in three types of phenylpropane subunits, namely, *p*-coumaryl alcohol, coniferyl alcohol, and sinapyl alcohol. The primary components of lignin are guaiacyl (G), syringyl (S), and *p*-hydroxyphenyl (H) units, interconnected by two common types of bond, corresponding to ether bonds (such as α-O-4, β-O-4, and 4-O-5) and carbon–carbon bonds (such as β-β, β-5, β-1, and 5-5) [5]. Within the walls of plant cells, certain polysaccharides can be associated with the lignin network via alkyl/aryl-ether bonds to form lignin–carbohydrate complexes. The enzymatic depolymerization of lignin has received substantial attention, due to its high efficiency and environmentally protective capabilities. However, the diverse chemical composition of lignocellulose hinders its biodegradation. Regarding the aim of further improving the performance of lignin-degrading enzymes, it is expected that lignin biodegradation in conjunction with multiple enzymes will contribute to the resolution of the above issues [6].

Lytic polysaccharide monooxygenases (LPMOs), a group of copper-containing redox enzymes, are widely recognized for their capability to oxidatively cleave the glycosidic bonds of polymeric carbohydrates via an oxygen-rebound mechanism, utilizing a reducing agent [7,8,9]. Based on the classification of the Carbohydrate-Active enzymes (CAZy) database (http://www.cazy.org (accessed on 6 March 2024)), LPMOs have been identified in eight auxiliary activity (AA) families, including AA9, AA10, AA11, AA13, AA14, AA15, AA16, and, more recently, AA17 [10,11]. The AA9 LPMO has been thoroughly studied in recent years, primarily because of its high enzymatic conversion efficiency in relation to plant biomass. In an LPMO-assisted enzymatic cellulose degradation, it was demonstrated that the addition of the AA9 LPMO can enhance the catalytic efficiency of commercial cellulase, resulting in an increase in the total saccharification yield to 23.9% and 48.5% [9]. It has been established that AA9 LPMOs can be utilized commercially, as they can increase the rate of cellulose degradation in the Cellic CTec 3 and Accelerase TRIO enzyme and further hydrolyze several cellulosic components and various cellulosic substrates, including cellulose, glucomannans, xyloglucans, β-glucan, etc. [9,12]. Moreover, low molecular reducing agents, including ascorbic acid, cysteine, glutathione, hydroquinone, etc., are commonly utilized as electron donors, boosting the catalytic efficiency of LPMOs [13]. While the AA9 LPMO is currently attracting significant research attention, there are still a number of obstacles to developing LPMO-containing cellulase, mainly due to the high costs of enzyme production and the complex structure of lignocellulosic biomass [6,12]. Therefore, designing multi-enzyme biocatalysts, comprising LPMOs and other low-cost enzymes, is established, as a strategy achieving the low-cost enzymatic degradation and high-yield conversion of lignocellulosic biomass.

Laccase, a kind of multicopper oxidase, is typically present in both fungal and bacterial microorganisms, exhibiting an extraordinary ability to oxidatively cleave a wide variety of phenolic substrates [1,14]. When using laccase for lignin depolymerization, the laccase-catalyzed oxidation of hydroquinone might help to generate reactive oxygen species through reactions of lignin with O_2_, resulting in the formation of hydrogen peroxide [15]. Additionally, it has been ascertained that a suitable concentration of hydrogen peroxide has a beneficial effect on the initiation of LPMO, yet the reducing agents remain indispensable for activating copper ions at the active site [4,15,16]. Hence, the oxidation products of laccase-treated lignin may enhance LPMO activity. Furthermore, low-molecular-weight phenolic compounds from laccase-treated lignin exhibit a positive impact on the LPMO oxidation of cellulose, whereby cellulose substrates are degraded via electrons provided by soluble low-molecular-weight lignin via long-range electron-transfer pathways [17,18]. However, another study concluded that the employment of lignin-degrading enzymes, including laccase, could generate phenoxy radicals during lignocellulose biodegradation [19]. These exhibit potential antioxidant capacities, leading to the stabilization of the lignocellulosic biomass [19].

Tyrosinase, categorized as polyphenol oxidase (EC 1.14.18.1), is a multifunctional copper-containing oxidase and widely used in the pretreatment of lignocellulosic biomasses, wherein it can efficiently catalyze the *ortho*-hydroxylation of monophenols to *ortho*-diphenols and the further oxidation of *ortho*-diphenols into *ortho*-quinone using O_2_ as a co-substrate [20]. The tyrosinase from *Myceliophthora thermophila* C1 appears to be capable of enhancing LPMO activity by oxidizing lignin building blocks, resulting in up to a 75-fold rise in the LPMO lignocellulose oxidation process [4]. Moreover, the C1-, C4-, and C1/C4-oxidizing AA9 LPMOs were found to be correlated with the physicochemical features and structural properties of lignin; in particular, low-molecular-weight-lignin-related compounds had a beneficial effect on the LPMO–cellulose interaction [21]. These findings indicate that LPMO activity towards cellulosic substrates is improved by lignin-derived phenolic compounds, which donate electrons to the enzyme and thus improve the processes of enzymatic hydrolysis [4,21]. 

Enzymatic lignocellulosic bioconversion is a critical factor in the success of industrial biorefineries dealing with plant biomass waste, appearing to play a significant role in both carbon cycle and economic sustainable development. So far, the mutation of a single residue in LPMOs has been proven to improve the cellulose oxidization abilities of AA9 LPMOs from *Myceliophthora thermophila* C1 [9]. However, few relevant studies have been conducted on mutant-type LPMOs with lignin-diverted reducing agents, forming a major obstruction to the utilization of point-mutation LPMOs. Therefore, the main purpose of our study is to provide further insights into the differences in LPMO activities between wild and mutant types in various reducing agents, in order to elucidate the interactions between LPMO and lignin-diverted phenolic compounds. The polyphenol oxidases, either laccase or tyrosinase, are incorporated into LPMO reactions, along with diverse reducing agents, to evaluate the effect of polyphenol oxidases on the performance of the LPMOs. Importantly, our experimental results, combined with previous observations, establish the effects of LPMOs’ catalytic efficiency when co-incubated with polyphenol oxidases and lignin-diverted small phenolic compounds. It is hypothesized that the small phenolic compounds acting as reducing agents are suitable for promoting LPMO activity, whereas the addition of polyphenol oxidases have different impacts on cellulose degradation. Above all, it is expected that our study will offer a deeper understanding of the interrelations between both wild and mutant types of LPMOs, cellulose, polyphenol oxidases, and lignin-diverted small phenolic compounds, which can be used to create a multi-enzyme lignocellulose degradation system.

## 2. Materials and Methods

### 2.1. Materials

The organosolv lignin (Chemical Abstracts Service (CAS) No.: 8068-03-9, C_81_H_92_O_28_), a dry brown powder comprising 95% lignin content and 3.98% moisture, was purchased from Hangzhou Dingyan Chem Co., Ltd (Jianggan, Hangzhou, China). Laccase from *Trametes versicolor* (CAS No.: 80498-15-3) was obtained from Shanghai Aladdin Biochemical Technology Co., Ltd. (Shanghai, China) and tyrosinase from mushroom (CAS No.: 9002-10-2) was purchased from Sigma-Aldrich (St. Louis, MO, USA). The microcrystalline cellulose (CAS No.: 9004-34-6, (C_6_H_10_O_5_)_n_), coomassie brilliant blue 5×G250 (PC0015), and 3,5-dinitrosalicylic acid (DNS) agents (D7800) were purchased from Beijing Solarbio Science & Technology Co., Ltd. (Beijing, China). Reducing agents or enzyme substrates, including 3-hydroxy-4-methoxycinnamic acid (C_10_H_10_O_4_), 3-methylcatechol (C_7_H_8_O_2_), 4-hydroxy-3-methoxycinnamic acid (C_10_H_10_O_4_), 4-hydroxy-3-methoxyphenylacetic acid (C_9_H_10_O_4_), 4-hydroxy-3-methoxyphenylacetone (C_10_H_12_O_3_), 4-hydroxybenzoic acid (C_7_H_6_O_3_), 2,2′-azinobis (3-ethylbenzothiazoline-6-sulfonic acid) (ABTS, C_18_H_18_N_4_O_6_S_4_), caffeic acid (C_9_H_8_O_4_), coniferyl alcohol (C_10_H_12_O_3_), gallic acid (C_7_H_6_O_5_), guaiacol (C_7_H_8_O_2_), guaiacylglycerol-β-guaiacyl ether (C_17_H_20_O_6_), *p*-coumaric acid (C_9_H_8_O_3_), sinapic acid (C_11_H_12_O_5_), vanillic acid (C_8_H_8_O_4_), and veratryl alcohol (C_9_H_12_O_3_), were purchased from Shanghai Aladdin Biochemical Technology Co., Ltd. (Shanghai, China). Sodium ascorbate (C_6_H_7_NaO_6_) and 5 mg/mL bovine serum albumin (CAS No.: 9048-46-8) were obtained from Shanghai Macklin Biochemical Technology Co., Ltd. (Shanghai, China). All other chemicals used were of an analytical grade.

### 2.2. Expression and Purification of Lytic Polysaccharide Monooxygenases (LMPOs)

Two kinds of AA9 LPMOs, including wild-type (WT) and mutant-type (R17L) LPMOs from *Myceliophthora thermophila* C1, were expressed and purified in accordance with the previous study, with some modifications [22]. In brief, the transformants of the LPMOs were selected for kanamycin resistance and grown in buffered glycerol complex medium (BMGY) at 30 °C and 200 rpm for 16–18 h. Afterwards, the cultures were centrifuged, and then the pellets were resuspended on a buffered methanol-complex medium (BMMY) with 1 mM Cu^2+^ (final concentration). The expression of the LPMOs was induced by the addition of methanol (5% *v*/*v*) to the BMMY every 24 h, and the cultures were grown at 30 °C in 200 rpm for 6 days. After expression, the cultures were centrifuged at 4 °C at 12,000 rpm, and then the supernatants were filtered on a 0.45 µm syringe filter (Polyethersulfone (PES) membrane, Nantong Longjin Membrane Technology Co., Ltd.). Recombinant proteins were purified from the filtered supernatants using a nickel-nitrilotriacetic acid (Ni-NTA) Superflow resin column (Qiagen, Hilden, Germany), and a binding buffer (20 mM Tris-HCl, 500 mM NaCl, 20 mM imidazole, 1 mM dithiothreitol, pH 7.4) was used to wash the column to reduce non-tagged protein binding; this was followed by the use of an elution buffer (20 mM Tris-HCl, 500 mM NaCl, 500 mM imidazole, 1 mM dithiothreitol, pH 7.4) to recover His-tagged proteins. Finally, the purified LPMOs were desalted in a 3.5 kDa dialysis bag using a desalting buffer (20 mM Tris-HCl buffer, 100 mM NaCl, pH 7.4). The purity of the LPMOs was evaluated using Sodium dodecyl sulfate-polyacrylamide gel electrophoresis (SDS-PAGE), and the enzyme concentration was determined via coomassie brilliant blue G-250, using bovine serum albumin (5 mg/mL) solutions as standards.

### 2.3. Assessment of Lytic Polysaccharide Monooxygenase (LMPO) Activity

The LPMO activities in different reducing agents were evaluated as described previously, with some modifications [4,22]. The LPMO activity was determined in mixtures containing 1 mg/mL microcrystalline cellulose for 1 mM reducing agents in 25 mM pH 7.5 Tris-HCl buffer (final concentrations). The enzymatic reactions were performed at 1 μM of LPMO by adding water, laccase (to a final concentration of 0.18 μM), or tyrosinase (to a final concentration of 0.24 μM). For the light-sensitive reducing agents, the LPMO reaction was carried out in darkness. The enzymatic reaction was monitored at 45 °C for 48 h. Afterwards, cellulase was added to the reaction mixtures to a final concentration of 1.4 U/mL, and then incubation continued for 12 h. After incubation, the enzymatic reactions were stopped by heating at 95 °C for 10 min, followed by centrifugation (12,000× *g*, 5 min) to separate the supernatants. Finally, the concentrations of reducing sugars in the supernatants were determined via the DNS method using glucose (ranges from 0 mM to 10 mM) as standards [23]. 

### 2.4. Simultaneous Reactions of Lytic Polysaccharide Monooxygenase (LMPO) and Polyphenol Oxidase

The effects of lignin and its enzymatic hydrolysis products on LPMO activity were evaluated in accordance with the previous study [15]. The organosolv lignin was first washed five times using water (10% *w*/*w*), in order to remove the soluble parts from the substrate. The washed organosolv lignin sample was prepared by diluting the concentration of 5% *w*/*w* in 25 mM Tris-HCl buffer. For the purpose of establishing a one-pot reaction, 900 µL of organosolv lignin suspension (5% *w*/*w*) was added to 220 µL of the enzymatic reaction mixture, which contained 1 μM LPMO together with 10 mg/mL microcrystalline cellulose; then, 1 mM sodium ascorbate was added to the 25 mM Tris-HCl buffer with a pH of 7.5. The one-pot reaction was initiated by mixing 80 µL of either Tris-HCl buffer, 0.09 μM laccase, or 0.12 μM tyrosinase (final concentration). The experimental setups for the supernatants from the oxidization products of the polyphenol oxidases were identical to those used for the assessment of LPMO activity. The conditions and processes of the one-pot reactions were in line with the previously described methods for assessing LPMO activity.

### 2.5. Oxidation Products of Laccase and Tyrosinase

The organosolv lignin degradation product profiles were investigated for laccase and tyrosinase using 10% (*w*/*w*) of organosolv lignin in water at 45 °C for 48 h. The concentrations of enzymes in the enzymatic reaction were 0.014 μM laccase or 0.018 μM tyrosinase (final concentration). The samples were heated for inactivation at 95 °C for 10 min and centrifuged for 10 min at 9000 rpm as the enzymatic reaction was completed. Lastly, all samples were filtered (0.22 µm) to remove small insoluble substrate for the LC-MS analysis.

The compositions of laccase and tyrosinase hydrolytic products were determined via LC-MS, according to the method used in the previous study [15]. The samples were evaluated by injection into a Hypersil Gold Phenyl column (150 mm × 2.1 mm; 3 μm, Thermo Fisher Scientific, Waltham, MA, USA) with a 0.4 mL/min flow rate at 40 °C. Three types of eluents, eluent A (0.1% formic acid in water), eluent B (acetonitrile), and eluent C (water), were used for elution, which took place according to the following steps: from 0 to 15 min, 10% A, linear gradient of B from 0 to 90% and linear gradient of C from 90 to 0%; from 15 to 20 min, 10% A, 90% B, and 0% C; from 20 to 25 min, 10% A, 0% B, and 90% C. The electrospray was operated in both positive and negative scan modes to collect the absorbance data from 190 to 700 nm using a target mass of 200 *m*/*z* with a scan range of 50 to 2200 *m*/*z*. The spray parameters were set as follows: a capillary voltage of 4.5 kV, a nebulizer pressure of 3.0 bar, a 12.0 L/min drying gas flow, a gas temperature of 280 °C, and 0.5 kV of end-plate offset. The results of the EICs (extracted ion chromatograms) together with *m*/*z* and MS^2^ fragmentation data were generated using MassLynx V4.1 (Waters Corporation, Milford, MA, USA).

### 2.6. Protein Structures and Electrostatic Plots of Lytic Polysaccharide Monooxygenases (LMPOs)

The protein sequences of both wild and mutant types of LPMO were acquired according to the method used in our previous study [9]. Homology models for the two types of LPMOs, WT and R17L, were created using the Homology Modeling function of the SwissModel (https://swissmodel.expasy.org/interactive (accessed on 6 March 2024)), with NcLPMO9F from *Neurospora crassa* (PDB ID: 4QI8) as the template for the protein structure prediction [24,25]. Both the wild and mutant models of LPMO presented excellent root-mean-square deviation values (0.163 Å) and more than 76% of the sequence identities compared with the template, as calculated through the built-in functions of UCSF Chimera [9,24,25,26]. The homology models of the LPMOs were presented using UCSF Chimera as both surface and ribbon plots, and then electrostatic potential surfaces of the LPMOs were created using the built-in function of electrostatic potential according to Coulomb’s law [26].

### 2.7. Optimal pH and Temperature Conditions of Laccase and Tyrosinase

The optimal reaction conditions of laccase and tyrosinase were analyzed using the response surface method (RSM) and central composite design (CCD) for two factors (pH and temperature) and three levels (Table S2). The enzymatic reaction conditions, such as the reaction time, pH and temperature ranges, and concentrations of the substrate and enzyme, were established according to previous studies and preliminary experiments [18,20]. The laccase activity was determined using a concentration of 1 mM 2,2′-azinobis (3-ethylbenzothiazoline-6-sulfonic acid) (absorbance at 420 nm) in a 25 mM sodium acetate buffer at an enzyme concentration of 0.27 μM. The RSM experiment for laccase was conducted by selecting a pH range from 4.0 to 7.0 and a temperature range from 30 °C to 60 °C as the appropriate parameter ranges. The center point of laccase in the RSM experiment was pH 5.5 and 45 °C. The definition of one activity unit (U) of laccases was established by the amount of the enzyme converting 1 μmol of catechins per minute at 25 °C and a pH of 5.0. Moreover, the tyrosinase activity was determined based on the conversion of 10 mM veratryl alcohol (absorbance at 310 nm) under a 0.36 μM enzyme concentration in a 25 mM sodium phosphate buffer. The RSM reaction conditions for tyrosinase were optimized within a pH range of 5.5 to 8.5 and a 15 °C to 45 °C incubation temperature, where the conditions of the center point were pH 5.5 and 45 °C. One unit of tyrosinase activity (U) was quantified as 1 μmol 3,4-dihydroxy-L-phenylalanine converted from tyrosine per minute. Lastly, the absorbance of the enzymatic product was regarded as the optimized response using JMP Pro 17.0.0 (SAS Institute Inc., Cary, NC, USA) for the RSM calculation.

### 2.8. Kinetic Parameters of Laccase and Tyrosinase

The kinetic constants of laccase were determined for different concentrations of ABTS, ranging from 0.1 mM to 1.2 mM within an enzyme concentration of 0.27 μM at an optimal pH and 45 °C for 5 min. The maximum reaction rates (mM/min) were applied for the calculation of kinetic constants. The standard curve of ABTS was established via over-oxidation with the following substrate concentrations, ranging from 0 mM to 0.15 mM. Furthermore, the kinetic constants of tyrosinase were confirmed by 0 mM to 5 mM of veratryl alcohol concentrations using an enzyme concentration of 0.18 μM at an optimal pH and temperature for 12 h. The kinetic constants of tyrosinase were determined in reaction rates (mM/min). Veratraldehyde was used to prepare the standard curve (ranging from 0 mM to 1 mM). All the other parameters for the enzymatic reaction were the same as those originally configured in the RSM experiment. The kinetics of laccase and tyrosinase were analyzed via linear regression in a Hanes–Woolf plot.

The thermal stability of laccase was established in temperatures ranging from 60 to 85 °C at different inactivation times of up to 1 h. Afterwards, the activity of laccase was measured under optimal reaction conditions on 1 mM ABTS using an enzyme concentration of 0.18 μM for 10 min. Furthermore, the tyrosinase was first incubated at different temperatures (ranging from 50 to 75 °C) at relevant time intervals up to 1 h. The tyrosinase activity was subsequently determined under optimal reaction conditions at an enzyme concentration of 0.27 μM using 10 mM veratryl alcohol for 12 h. The enzymatic reactions were performed as previously described for the RSM experiment.

### 2.9. Statistical Analysis

The experimental results are expressed as the mean ± standard deviation. The determination of statistical significance was established via one-way analysis of variance (ANOVA, *p* < 0.05) with Turkey’s multiple comparison tests using OriginPro 2021 (OriginLab Corporation, Northampton, MA, USA).

## 3. Results and Discussion

### 3.1. Simultaneous Reactions of Lytic Polysaccharide Monooxygenase (LMPO) and Polyphenol Oxidase

An investigation of one-pot reactions involving the collaborative action of polyphenol oxidases and LPMOs made it possible to determine how LPMO reactions were affected by factors such as lignin-degrading enzymes, lignin substrates, enzymatic lignin oxidation products, etc. First, the effects of laccase on organosolv lignin as a substrate were investigated via Liquid Chromatography Mass Spectrometry (LC-MS) analysis (Figure S3a–c), which revealed that the samples comprised small soluble aromatic phenolic compounds, thus indicating that a depolymerization of the organosolv lignin substrate occurred after treatment with laccase. These findings corroborate prior investigations into the enzymatic reaction between laccase and organosolv lignin, where small phenolic compounds were present in the laccase oxidation products [15]. 

**Figure 1:**
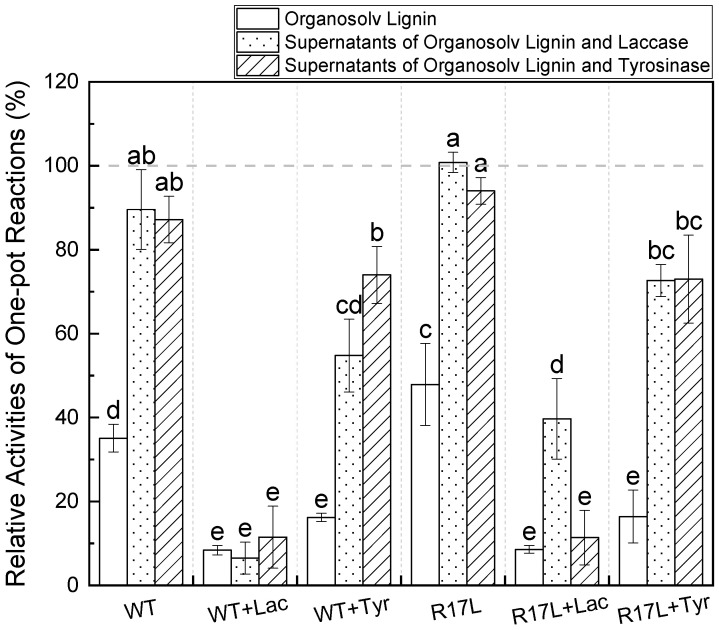
One-pot reactions containing a polyphenol oxidase (laccase or tyrosinase), LPMO, microcrystalline cellulose, and a lignin substrate (organosolv lignin, lignin degradation products of laccase, or lignin degradation products of tyrosinase). The total release of the reducing sugar concentration from R17L using sodium ascorbate as the reducing regents is equal to 100% (gray dashed line). The significant (*p* < 0.05) differences are marked by different letters (a–e) within the same item.

Taking into consideration the earlier discoveries of lignin oxidized by enzymatic activity, the one-pot reactions were designed to explore the synergistic relationships between polyphenol oxidases and LPMOs. The activities for both the wild-type LPMO (WT) and the point-mutant type of LPMO (R17L) were significantly (*p* < 0.05) decreased by around 50% when organosolv lignin was present in the enzymatic reactions, compared to the competitors of enzymatic reaction supernatants (Figure 1). Compared with WT, R17L exhibited significantly (*p* < 0.05) higher activity towards cellulose, a finding that aligns with a previous study, which showed that the engineered type of LPMO presented a specific activity 1.8 times higher than that of the wild-type LPMO [9]. Furthermore, it is evident that utilizing the organosolv lignin degradation products of laccases and tyrosinase can lead to an apparent promotional effect on LPMOs’ activities compared with the control using sodium ascorbate as reducing agent, while no apparent differences were found in the LPMOs’ activities toward two enzymatic lignin products (Figure 1). This result is in agreement with prior research, demonstrating that the laccase degradation supernatant of lignin enriched in low-molecular-weight lignin-derived compounds has a beneficial effect on LPMO cellulose hydrolyzation [18]. According to the LC-MS analysis of small phenolic compounds found in supernatants, our research suggests that these molecules can boost the cellulose hydrolysis of LPMOs. This agrees with a previous study’s finding that low-molecular-weight phenolic compounds from lignin can transfer electrons to LPMOs by means of long-range electron transfer [17]. The one-pot reaction samples in both organosolv lignin and the enzymatic lignin oxidation products without laccase or tyrosinase exhibited significantly (*p* < 0.05) or insignificantly (*p* > 0.05) higher LPMO activity than the groups with the polyphenol oxidase (Figure 1). This finding is aligned with previous studies showing that the consumption of oxygen during the simultaneous enzymatic depolymerization of cellulose and organosolv lignin creates a heightened level of competition for oxygen molecules, thus causing a reduction in LPMO activity [15,20]. 

After the addition of laccase in the LPMO reaction, all three types of one-pot reaction samples, consisting of two enzymatic lignin oxidation supernatants, exhibited a significant (*p* < 0.05) or insignificant (*p* > 0.05) decline in LPMO activity (Figure 1) compared to the samples to which tyrosinase was added; this was due to the high redox potential of laccase. It is a widely accepted fact that a high redox potential is the most notable feature of laccase, which is highly effective in the oxidation phenolic substrates through a four-electron reduction of oxygen to water. Our study thus revealed a negative correlation between the catalytic proficiency of LPMOs and the redox potential, particularly in tyrosinase, which is not regarded as an enzyme with high redox potential. The performance of the LPMOs is inhibited because of the oxidation of small phenolic compounds (e.g., ferulic acid) into phenoxyl radicals by laccase, which subsequently continues with coupling-based polymerization or a radical rearrangement [14,27]. These unstable phenoxyl radicals are not accessible to LPMOs [15,27]. Even though compounds derived from low-molecular-weight-lignin generated by laccases have the potential to donate electrons to LPMOs, the anoxic environment is a hurdle to improving cellulose hydrolysis yields [18]. Furthermore, the enzymatic products of lignin-derived compounds, some of which contain non-methoxylated monophenols, are probably oxidized by tyrosinase to 1,2-benzenediol moieties, and the electrons released by hydrolyzing process are accepted by the LPMOs, thereby enhancing the enzymatic cellulose oxidation process [4]. Above all, the findings based on the one-pot reactions lead us to the important conclusion that the small phenolic compounds produced by enzymatic oxidization in organosolv lignin may be beneficial in promoting LPMOs’ cellulose depolymerization process, whereas the enzymatic reactions between lignin oxidization and cellulose degradation should be separated in order to meet the oxygen prerequisites for these enzymes. 

### 3.2. Evaluation of Lytic Polysaccharide Monooxygenase (LMPO) Activities 

To ascertain the effect of the reducing agents derived from lignin building blocks and polyphenol oxidases (laccase and tyrosinase) on the activities of LPMOs, this study conducted experiments on both wild-type and mutant-type LPMOs with the aim of assessing their performance. Correctly understanding the interactions between LPMOs, enzymatic lignin biomass oxidization products, and polyphenol oxidases is crucial to constructing an advanced multi-enzyme approach for achieving lignocellulose biomass degradation. At first, no obvious change in the concentrations of the reducing sugars was observed when only reducing agents and cellulose were present in the reaction (data not displayed), suggesting that reducing agents may not have the ability to hydrolyze cellulose. This conclusion has been confirmed by other researchers, who believe that the use of a reducing agent alone (without a cellulose auto-oxidation enzyme) cannot degrade or release oxidized/non-oxidized gluco-oligosaccharides during the cellulose oxidation process [4]. The presence of the LPMOs’ activities showed a nearly identical reducing agent specificity between wild-type and mutant-type LPMOs when utilizing small phenolic compounds as electron donors for cellulose oxidation. Certain types of reducing agents, such as natural phenolic compounds that are either free or used as lignin building blocks, have also exhibited the capacity to improve LPMO activity when used as intrinsic electron donors [28]. Specifically, the activity of LPMO together with the laccases was the highest in the R17L group but the weakest in the WT group (Table 1). The observed changes in WT activity after the addition of tyrosinase were not obvious in the majority of the reducing agents when compared to the group without polyphenol oxidases. 

#### 3.2.1. Monophenols Group Reducing Agents

In comparison, some R17L activities in tyrosinase showed significant (*p* < 0.05) increases in relation to reducing agents, such as 4-hydroxybenzoic acid, *p*-coumaric acid, 4-hydroxy-3-methoxyphenylacetone, and sinapic acid, when compared to the group without polyphenol oxidases (Table 1). A previous study achieved an improvement in LPMO activities through the incubation of non-methoxylated monophenols (group 1a: 4-hydroxybenzoic acid and *p*-coumaric acid) and AbPPO, when compared with reducing agents alone, which is in accordance with our findings [4]. These increases have a significant influence on the R17L activities (*p* < 0.05), albeit with decreased activities being noted for other agents, including coniferyl alcohol, vanillic acid, 3-methylcatechol, and caffeic acid (Table 1). However, a few reducing agents displayed significantly (*p* < 0.05) higher levels of activity after the addition of tyrosinase, including *p*-coumaric acid, guaiacol, and 4-hydroxy-3-methoxyphenylacetone (Table 1). The same finding is presented in a previous study, which found that the activities of MtLPMO9B were enhanced in the presence of these three reducing agents, following the supplementation of AbPPO, when compared to the group of lignin-degrading enzymes [4]. A newly discovered polyphenol oxidase from *Myceliophthora thermophila* demonstrated outstanding efficiency in converting monophenols derived from lignocellulose, resulting in the generation of guaiacol oxidation products that served as potent reductants, ultimately boosting enzyme activity via electron donation to LPMOs [29]. Nevertheless, there were no significant (*p* > 0.05) changes in LPMO activities among a limited number of reductants, including WT in *p*-coumaric acid and R17L in guaiacol, sinapic acid, and guaiacylglycerol-β-guaiacyl ether (Table 1). A steady rise in the release of non-oxidized and C1-oxidized gluco-oligosaccharides in MtLPMO9B was also observed in a previous study when guaiacol and tyrosinase were co-incubated [4]. Intriguingly, our findings further indicate a significant (*p* < 0.05) or insignificant (*p* > 0.05) reducing trend in LPMO activities in the majority of reducing agents, following the addition of laccase, compared with other groups. Furthermore, the reference, which contains reducing agents (such as coniferyl alcohol, vanillic acid, etc.) without LPMOs exhibited a higher value of reducing sugars compared to the group co-incubated with laccase and LPMOs. This is likely due to the second inhibition of laccase through the chemical modification of the hydroxyl group and methylene groups on cellulose substrates [18].

#### 3.2.2. Other Reducing Agents

There were no significant differences (*p* > 0.05) among activities for both wild-type and mutant-type of LPMOs in reducing agents without polyphenol oxidases, with the exception of caffeic acid, which presented significantly (*p* < 0.05) higher activity in R17L in comparison to WT (Table 1). The activities of LPMOs followed variation trends similar to the addition of laccase to the reducing agents in comparison to the other two groups and in contrast to gallic acid, which presented the opposite results. Unlike gallic acid and 3-methylcatechol, the activities of the LPMOs with tyrosinase in most of the reducing agents were significantly (*p* < 0.05) higher than the group with laccase. However, 3-methylcatechol exhibited the highest LPMO activity levels after the addition of laccase. The restriction of the cellulose oxidation of LPMOs featuring laccase and reducing agents primarily occurs because of the strong redox potential of laccase, along with oxygen competition, when it is regarded as a co-substrate for both enzymatic reactions [15,18]. A possible reason for the enhancement of LPMO activities, regarding the incubation of 3-methylcatechol and laccase, concerns the generation of by-products during the lignin oxidation process, such as laccase-oxidized phenolic compounds or hydrogen peroxide, which are regarded as reducing agents or electron donors for LPMOs and contribute to the oxidative degradation of cellulose [4,15]. Two fungal oxidoreductases belonging to the novel AA16 family exhibit the capability to generate hydrogen peroxide by oxidizing low-molecular-weight reductants, obviously enhancing the activity of four LPMOs from *Myceliophthora thermophila*, while other enzymes from *Neurospora crassa* demonstrate the opposite trends [30]. By comparing the activities of LPMOs with added laccase to the group without polyphenol oxidases, significant (*p* < 0.05) or insignificant (*p* > 0.05) improvements were discovered simultaneously for a few reducing agents (Table 1), including *p*-coumaric acid, guaiacol, 3-methylcatechol, and gallic acid (only in R17L). As compared to WT, a significantly (*p* < 0.05) higher activity level of R17L was discovered when sodium ascorbate was used as the reducing agent, which appeared to show a remarkable impact on LPMO activities compared to lignin-derived reducing agents other than 3-methylcatechol and gallic acid (Table 1). Certainly, some reducing agents, such as sodium ascorbate and gallic acid, are capable of supplying electrons to LPMOs to oxidate cellulose in the absence of polyphenol oxidases. Numerous studies have concluded that sodium ascorbate and ascorbic acid are efficient reducing agents for LPMO activity [9,21]. The LPMOs’ activities in 3-methylcatechol and gallic acid without polyphenol oxidases were revealed to be significantly (*p* < 0.05) higher than the WT when sodium ascorbate was used as the reducing agent, while no significant (*p* > 0.05) differences in activities between WT and R17L in the same reductant. This result is in agreement with a previous study, which found that 3-methylcatechol (containing a 1,2-benzenediol moiety) and gallic acid (containing a 1,2,3-benzenetriol moiety) released extremely high levels of oxidized and non-oxidized gluco-oligosaccharides during the LPMO reaction [28]. There were lower levels of LPMO activity in group 1a (4-hydroxybenzoic acid and *p*-coumaric acid) than groups 2 (3-methylcatechol and caffeic acid) and 3 (gallic acid), primarily because of the higher oxidation potential of monophenol compounds compared to other phenolic compounds comprising 1,2-benzenediol or 1,2,3-benzenetriol moieties, which weaken the reducing potential of copper at the active site of the LPMOs [28,31]. The reduction of copper ions at the active site of the LPMOs can be enhanced by phenolic compounds with either a 1,2-benzenediol or 1,2,3-benzenetriol moiety, due to their low redox potential, which can stabilize the delocalization of π-electrons by extra hydroxyl groups via the resonance effect [31,32]. The electron donation capacity of the reducing agents is easily affected by the influences of electron-donating or electron-withdrawing groups in substituted aromatic rings [33,34]. In conclusion, tyrosinase with a low redox potential is preferable to high-redox-potential laccase, thereby improving the cellulose conversion of LPMO in the presence of lignin-derived small phenolic compounds.

### 3.3. Insights into Protein Structure and Reducing Agent Specificity for Lytic Polysaccharide Monooxygenase (LMPO) Reactions

**Figure 2:**
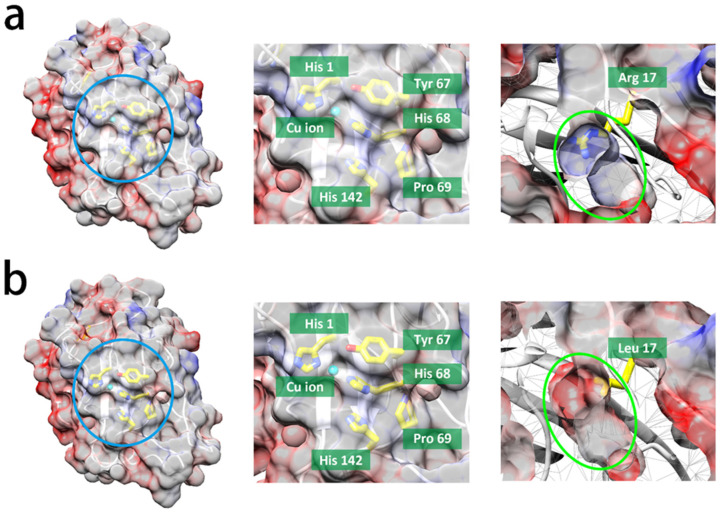
Homology models of LPMOs predicted by the NcLPMO9F template from the *Neurospora crassa* (Protein Data Bnak ID: 4QI8) alignment from SwissModel [35,36]: (**a**) WT and (**b**) R17L. Left: the surface structure of LPMOs along with the electrostatic potential (blue or red represent the electrostatic potential being either positive or negative, respectively). Middle: Magnification homology models of LPMOs around the copper ion with active sites (light blue circle). Right: Magnification homology models of LPMOs at mutant positions (green circle). The elements of carbon, copper, sulfur, and oxygen are represented by yellow, cyan, blue, and red, respectively.

The copper ion (yellow) present in either WT or R17L is encircled by positively charged amino acids, exhibiting nearly identical electrostatic potential (Figure 2). Additionally, it is also surrounded by essential residues for cellulose degradation in LPMOs (Figure 2a left and Figure 2b left), including His 1, Tyr 67, His 68, Pro 69, and His 142 [22,26]. This figure also shows that the models of WT and R17L have a different residue at position 17, where arginine is present in WT and leucine is present in R17L (Figure 2a right and Figure 2b right). While these residues are located far away from the copper ion and exhibit varying electrostatic characteristics, R17L demonstrates a more positive charge than WT (Figure 2a right and Figure 2b right). Despite the more positively charged R17L at location 17 (Figure 2a middle and Figure 2b middle), no substantial variations in LPMO activities were detected in the two enzymes, following the addition of the reducing agents, demonstrating that identical protein structures and electrostatic potential may result in reducing agents with similar specificities. Our study considered that the influences of the mutated residue on LPMO activity might be largely dependent on the location of the residue mutation, leading us to the possible conclusion that the shorter the distance between the mutation residue and the copper ion, the greater the effect of the LPMO cellulose oxidation will be.

**Figure 3:**
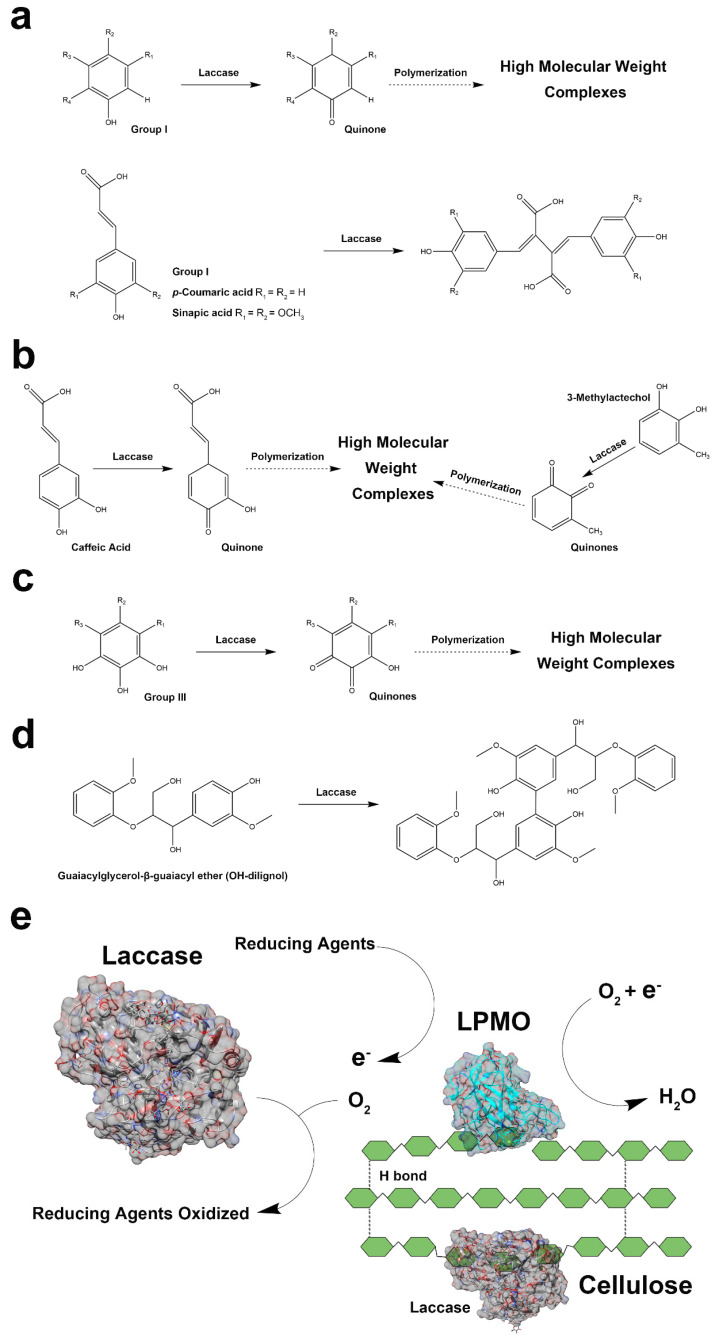
Possible reaction routes whereby laccase oxidates different reducing agents (**a**–**d**), and a proposed schematic representation of laccase-oxidized reducing agents involved in LPMO cellulose degradation (**e**). The dotted arrows indicate a possible route for laccase oxidation.

**Figure 4:**
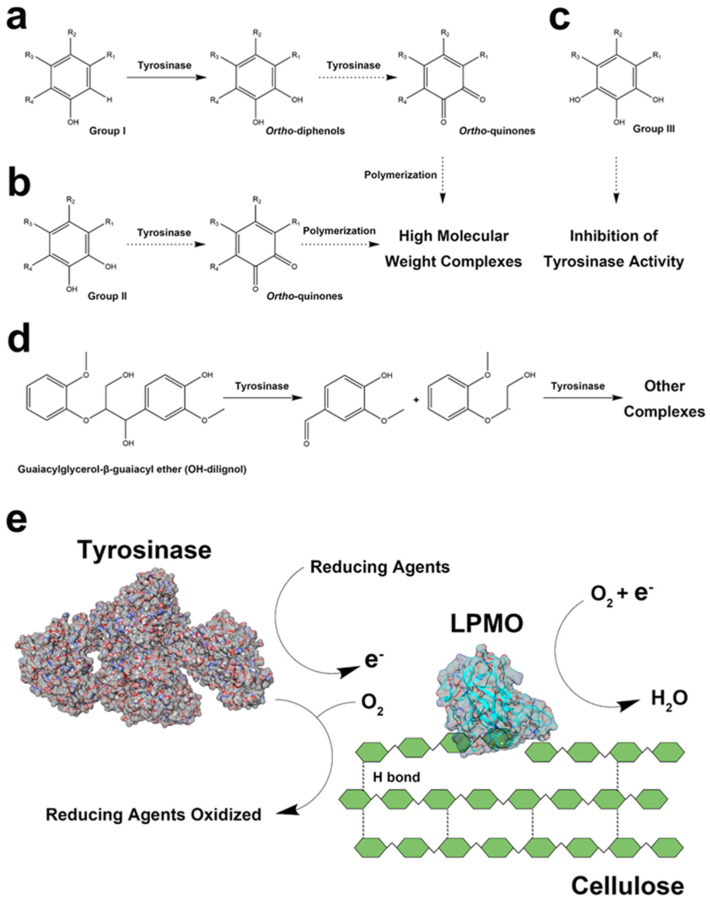
Possible reaction routes whereby tyrosinase oxidates different reducing agents (**a**–**d**) and a proposed schematic representation of tyrosinase-oxidized reducing agents involved in LPMO cellulose degradation (**e**). The dotted arrows indicate a possible route for tyrosinase oxidation.

The prediction of the enzymatic oxidization routes for various reducing agents by laccase were confirmed by previous studies (Figure 3a–d) [37,38,39,40,41,42]. During the laccase oxidization process, phenolic compounds such as 4-hydroxybenzoic acid, 4-hydroxy-3-methoxycinnamic acid, coniferyl alcohol, and caffeic acid are converted to an unstable quinone (Figure 3a,b), resulting in the formation of high-molecular-weights complexes via the repolymerization process [37,38,39,41]. Furthermore, laccase has the ability to oxidize 3-methylcatechol and gallic acid (Figure 3b,c), generating two unstable quinones that can promote the polymerization of high-molecular-weight complexes [37,43]. Nonetheless, the formation of complexes in *p*-coumaric acid, sinapic acid, and guaiacylglycerol-β-guaiacyl ethers via laccase exhibited an alternative pathway, in contrast to other reducing agents (Figure 3a,d) [40]. The figure illustrates the simultaneous interactions between LPMO and laccases (Figure 3e) and highlights both the competitive inhibition and cellulose modification of laccase during the LPMO cellulose degradation process. Moreover, tyrosinase displayed similar traits (Figure 4a,b), which led to the formation of high-molecular-weight compounds via the formation of *ortho*-quinones [4]. In particular, a study recently implemented the methyl hydroquinone as a phenolic reducing agent in the LPMO reaction, resulting in reduced enzyme activity primarily caused by oxidative damage and irreversible inactivation observed in various fungal LPMOs [44]. The inhibitory effect of gallic acid on tyrosinase activity was observed (Figure 4c), and two low-molecular-weight compounds were formed when tyrosinase oxidized the guaiacylglycerol-β-guaiacyl ether (Figure 4d), subsequently forming other complexes [20,45]. The enzymatic reaction mechanism for tyrosinase and LPMO is demonstrated in Figure 4e, indicating the competitive inhibition among enzymes, whereas tyrosinase-oxidizing reductants can possibly stimulate LPMO activity. These findings regarding polyphenol oxidases may account for the decline in LPMO activity observed after the addition of polyphenol oxidases when using lignin degradation products as reducing agents, probably due to the formation of high-molecular-weight complexes by polyphenol oxidases.

The single hydroxyl group in phenolic compounds is not energetically favorable for donating electrons as a result of the energetic disorder of the π-electron aromatic sextet. Compared to laccase, our study hypothesized that there would be an improvement in the LPMO activities of multiple reducing agents after the addition of tyrosinase, on the basis of its outstanding hydroxylating activity towards the hydroxyl group. In previous studies, tyrosinase was found to be particularly effective in various phenolic compounds, especially those featuring a 1,2-benzenediol or 1,2,3-benzenetriol moiety [4]. The tyrosinase hydroxylation of the hydroxyl group of phenolic compounds can form *ortho*-diphenols, which are likely to serve as viable electron donors for LPMOs [4]. In contrast to hydroxyl groups, laccase exhibits limited activity towards these moieties and utilizes oxygen to oxidize phenolic compounds simultaneously, subsequently leading to the weakened degradation capacities of cellulose by LPMOs [18]. Laccase, with its high redox potential, has the capacity to strengthen cellulose via the incorporation of modification moieties on the cellulose surface, such as hydroxyl and methylene groups, negatively affecting the binding affinity of the cellulose substrate [18,46,47]. Although laccase has the ability to produce lignin-derived phenolic compounds to improve LPMO activities, the inhibitory effects of laccases, which include changing the structure of cellulose and improving the binding affinity of substrates between cellulose and enzymatically modified lignin, have been an obstacle to enhancing cellulose oxidation with LPMOs [18,47,48,49,50]. The products of tyrosinase and laccase from organosolv lignin exhibit a close association with lignin-derived small phenolic compounds from the enzymatic degradation of plant biomass, which are believed to be excellent potential reducing agents for LPMOs. This conclusion corresponds with that of a previous study, which showed that using the supernatants from laccase-pretreated native sugarcane bagasse and wheat straw as reducing agents is a feasible approach to improving the LPMO oxidation of cellulose [18].

### 3.4. Optimal Reaction Conditions of Laccase and Tyrosinase

**Figure 5:**
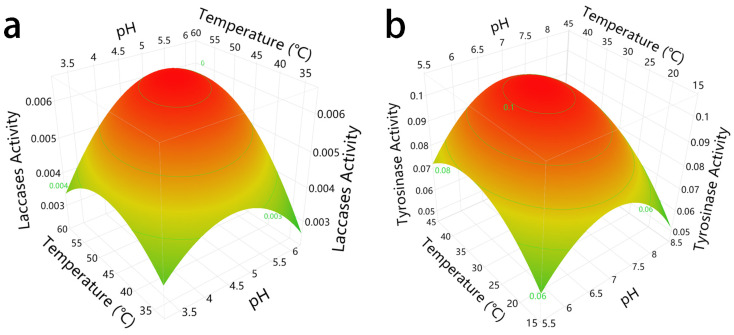
The optimal pH and temperature of laccase and tyrosinase based on response surface methods (RSM) generated using central composite design (CCD). (**a**) The optimal pH and temperature conditions of laccase were 5.19 and 53 °C, respectively (R^2^ = 0.9505, Table S1). (**b**) The optimal pH and temperature conditions of tyrosinase were determined to be 7.01 and 35 °C, respectively (R^2^ = 0.8630, Table S1).

The identification of optimal reaction conditions of polyphenol oxidases is essential for establishing a suitable enzymatic reaction environment for lignin biodegradation, offering opportunities for the multi-enzyme conversion of lignocellulose biomass. The optimal pH and temperature conditions of laccase were determined to be 5.19 and 53 °C, respectively, using 1 mM 2,2′-azinobis (3-ethylbenzothiazoline-6-sulfonic acid) (ABTS) (Figure 5a and Table S1). A previous study established that the optimal pH range of laccase from *Trametes versicolor* (purchased from Sigma-Aldrich, St. Louis, MO, USA) was between pH 5.0 and 7.0 when evaluated at 25 °C using ABTS as the substrate [51]. The parameter estimates for the laccase response surface method (RSM) results indicated that both the pH and temperature exhibited significant effects (*p* < 0.05) on laccase activity. The optimal enzymatic reaction conditions of tyrosinase, meanwhile, were also exhibited at an optimum pH of 7.01 and an optimal temperature of 35 °C on veratryl alcohol (Figure 5b and Table S1). These conclusions are consistent with an earlier study, which revealed that the optimal reaction conditions of tyrosinase from button mushrooms were pH 7.0 and 35 °C when using a potassium phosphate buffer [52]. According to the parameter estimation of the tyrosinase RSM models, the temperature had a significant effect (*p* < 0.05) on tyrosinase activity, whereas the pH exhibited an opposite trend (*p* > 0.05). The statistically insignificant interaction between pH and tyrosinase activity indicates that changes in tyrosinase activity occur slowly within a specific range of pH values, which corresponds to a previous study that found that the tyrosinase from *Agaricus bisporus* shows remarkably stable enzymatic activity across a wide range of pH values [52]. The model evaluations of laccase and tyrosinase exhibited a significant lack of fit (*p* < 0.05) on regression models, suggesting that the generated RSM models were deficient in the prediction of enzymatic activity under optimal conditions, probably owing to the assumption that quadratic polynomials are suitable for representing the interactions between pH and temperature [53]. Nevertheless, this assumption is challenged by other researchers, who argued that the asymmetric bell-shaped curve is obviously not perfectly suited to the evaluation of the effect of pH and temperature on enzyme activity [53]. Yet, despite these disadvantages, the final conclusions regarding the optimal reaction conditions for laccase and tyrosinase merit further analysis. 

### 3.5. Kinetic Parameters of Laccase and Tyrosinase

The effectiveness and cost of enzymatic lignocellulose conversion are greatly influenced by the kinetic constants and thermal stabilities of polyphenol oxidases, making them crucial parameters for industrial applications. The kinetic constants of laccase in the presence of 1 mM ABTS are shown below (Table 2). A previous study revealed that the *K*_m_ value of CotA-laccase from *Bacillus subtilis* was 0.087 mM for ABTS, which is lower than the value found in our study [54]. This conclusion indicates that laccase exhibited a greater affinity for and catalytic efficiency towards ABTS, compared with CotA-laccase. However, the values of V_max_ and *K*_cat_ for CotA-laccase (0.059 mM and 35.64 s^−1^, respectively) were markedly higher than those observed in our study [54]. The commercial laccase used in our study exhibited an extraordinary level of thermal resistance (80 °C with a half-life of 8.27 min) with an ordinary value of *k*_cat_/*K*_m_. Other scientists who tested the thermal stability and catalytic efficiency using ABTS as a substrate concluded that the laccases from *Pycnoporus sanguineus* CS43 had excellent *k*_cat_/*K*_m_ values of 74.816 mM^−1^ s^−1^ in Lac I and 36.746 mM^−1^ s^−1^ in Lac II and also showed poor thermal stabilities at 80 °C, with half-lives ranging from 0.6 to 1.8 min [55].

The kinetic constants of tyrosinase were determined using 10 mM veratryl alcohol (Table 2). A previous study demonstrated that the kinetic constants of tyrosinase from *Agaricus bisporus* with 10 mM veratryl alcohol were 0.31 mM for *K*_m_, 0.12 s^−1^ for *k*_cat_, and 0.39 mM^−1^ s^−1^ for *k*_cat_/*K*_m_ [20]. As anticipated, the *K*_m_ values noted above were basically consistent with our study, while the turnover number (*k*_cat_) and specificity constants (*k*_cat_/*K*_m_) exhibited discrepancies compared with our results. Strangely, a notably low value of V_max_ in tyrosinase was observed, which was coupled with a long reaction time for the kinetic constants (12 h). A contributing factor to this issue is that the tyrosinase oxidation of the veratryl alcohol was induced by oxygen in the air, rather than hydrogen peroxide, resulting in a long enzymatic reaction time due to the absence of a strong oxidant as a reaction mediator. Through an examination of the thermal stability in tyrosinase, it was ascertained that tyrosinase exhibits remarkable thermal tolerance across a broad range of temperatures, especially at 75 °C with a half-life of 2.66 min. The thermal stability of tyrosinase from *Agaricus bisporus* using two substrates (L-tyrosine and 3,4-dihydroxy-L-phenylalanine) was determined by other researchers, who revealed the excellent thermal stability of tyrosinase in a wide temperature range of between 30 and 55 °C, as well as a broad range of half-lives, spanning from 3 days to less than 2 min [56]. Finally, it is challenging to compare the kinetic parameters of the enzymes analyzed in our study with those examined in other studies, primarily because of the potential impact of the experimental conditions on the enzymatic reactions, such as the use of different types of substrates, etc.

## 4. Conclusions

The mutation of single residues in LPMOs is an appropriate means of improving their hydrolytic efficiency; however, mutant types of LPMOs in various reducing agents have not been sufficiently studied in previous research. In the current study, the results indicated that lignin-diverted phenolic compounds generated by the oxidation of polyphenol oxidases can enhance the activity of both wild and mutant types of LPMOs, which might be weakened by the addition of polyphenol oxidases. In the presence of polyphenol oxidases, the activities of LPMOs were greatly enhanced with the addition of gallic acid, especially in mutant-type LPMOs. The efficiency of LPMOs can be further improved by the addition of tyrosinase and reducing agents like 4-hydroxy-3-methoxyphenylacetone or *p*-coumaric acid. Both the wild and mutant types of LPMOs exhibit identical characteristics when utilizing reducing agents as electronic donors, thereby negating the possibility of lignin-diverted phenolic compounds and reducing agents having any negative impact on the single mutation of LPMO. However, the wild-type of LPMO reached its highest activity when combined with laccase and 3-methylcatechol. Although the possible interaction between the mutation residue and the copper ion may have an impact on LPMO cellulose oxidation, it is essential to conduct a comprehensive evaluation of the biochemical and structural components in order to confirm this hypothesis. Furthermore, future studies must reassess the response surface model, reconsider potential modifications or alternative models, and investigate the necessity of accounting for additional factors or characteristics to more accurately illustrate the underlying response surface. Despite the fact that the multi-enzyme lignocellulose oxidization process is still under development, gaining a deeper understanding of the simultaneous interactions between lignin degradation by polyphenol oxidases and LPMO cellulose hydrolyzation could open up rich opportunities for enzymatic lignocellulose valorization, thereby promoting biorefinery processes and the biomass waste utilization.

## Figures and Tables

**Table 1:** Summary of LPMO activities under co-incubations in different reducing agents and polyphenol oxidases. The selection of functional groups is based on a previous study, including Group 1a: monophenols, Group 1b: phenolic compounds with a 1-hydroxy,2-methoxy moiety, Group 1c: phenolic compounds with a 1-hydroxy-2,6-dimethoxy group, Group 2: phenolic compounds with 1,2-benzenediols group, and Group 3: phenolic compounds with a 1,2,3-benzenetriol group [4]. The total release of the reducing sugar concentration from R17L using sodium ascorbate as the reducing regent is equal to 100%. The values highlighted either in *italics* or underlined are significantly (*p* < 0.05) higher than the values for WT or R17L using sodium ascorbate as reducing regents, respectively. Different letters for the same reducing agent indicate statistical significance (*p* < 0.05).

Group	Reducing Agents	Activity of R17L in Sodium Ascorbate Set to 100%					
		WT	WT + Laccase	WT + Tyrosinase	R17L	R17L + Laccase	R17L + Tyrosinase
1a	4-Hydroxybenzoic acid	73.6 ± 4.4 c	16.6 ± 12.6 e	69.5 ± 7.9 c	70.3 ± 5.2 c	42.4 ± 6.1 d	95.9 ± 3.7 ab
1a	*p*-Coumaric acid	51.6 ± 2.2 c	62.8 ± 4.4 bc	79.1 ± 4.2 b	45.5 ± 1.7 c	74.6 ± 9.4 b	*102.7 ± 19.1 a*
1b	Coniferyl alcohol	77.6 ± 0.7 bc	19.6 ± 1.8 d	90.1 ± 1.8 ab	85.2 ± 10.0 b	12.1 ± 5.5 d	63.9 ± 3.5 c
1b	Guaiacol	60.7 ± 1.0 d	69.4 ± 2.7 c	71.1 ± 3.3 c	61.3 ± 6.1 d	65.4 ± 5.0 cd	57.9 ± 1.1 d
1b	Vanillic acid	63.5 ± 0.6 c	8.20 ± 1.2 f	52.0 ± 3.6 d	64.3 ± 0.8 c	7.10 ± 0.3 f	43.2 ± 7.6 e
1b	3-Hydroxy-4-methoxycinnamic acid	66.4 ± 1.8 c	17.3 ± 1.0 e	71.6 ± 6.5 c	70.1 ± 4.9 c	28.4 ± 6.2 d	69.3 ± 1.8 c
1b	4-Hydroxy-3-methoxycinnamic acid	64.7 ± 8.5 c	21.3 ± 5.7 d	60.1 ± 0.5 c	60.3 ± 2.3 c	29.5 ± 5.0 d	69.4 ± 5.3 c
1b	4-Hydroxy-3-methoxyphenylacetone	60.1 ± 0.8 e	10.9 ± 0.1 g	*88.4 ± 4.2 b*	64.3 ± 3.5 e	26.1 ± 1.3 f	71.0 ± 0.4 d
1b	4-Hydroxy-3-methoxyphenylacetic acid	76.0 ± 1.8 bc	31.8 ± 6.0 e	73.7 ± 3.7 bc	66.6 ± 6.7 c	43.2 ± 3.3 d	70.2 ± 6.2 c
1c	Sinapic acid	69.9 ± 2.7 e	86.3 ± 5.3 bc	79.4 ± 7.0 cde	71.6 ± 3.3 de	80.8 ± 1.0 bcd	90.9 ± 5.9 ab
2	3-Methylcatechol	*96.8 ± 6.2 bc*	110.9 ± 0.7 a	*96.0 ± 2.0 bc*	*100.7 ± 5.0 b*	*103.6 ± 4.2 ab*	90.9 ± 4.3 cd
2	Caffeic acid	83.7 ± 3.4 cd	31.8 ± 1.4 e	69.7 ± 4.7 d	*98.7 ± 15.1 ab*	26.6 ± 1.2 e	84.8 ± 6.5 bc
3	Gallic acid	*111.3 ± 1.0 b*	77.1 ± 13.7 c	99.1 ± 5.4 bc	*113.0 ± 1.6 b*	171.3 ± 30.0 a	152.3 ± 5.9 a
other	Guaiacylglycerol-β-guaiacyl ether	59.0 ± 4.4 bc	44.9 ± 3.7 c	60.4 ± 4.4 b	63.4 ± 9.9 b	53.4 ± 2.7 bc	60.4 ± 4.4 b

**Table 2:** Kinetic parameters (Figure S1a,b) and thermal stabilities (Figure S2a,b) of laccase and tyrosinase. The V_max_ values for laccase and tyrosinase are calculated according to the oxidization rates (mM/min) of ABTS and veratryl alcohol, respectively. The *k*_cat_ represents the mM product released per mM enzyme per minute, and the molecular weights for the *k*_cat_ calculation are as follows: 64 kDa for laccases and 119.5 kDa for tyrosinase.

Name	V_max_ (mM/min)	*K*_m_ (mM)	*k*_cat_ (/min)	*k*_cat_/*K*_m_ (mM^−1^ min^−1^)	Temperature (°C)	*K*_d_ (min^−1^)	t_½_ (min)
Laccase	0.003827	0.1004	14.12	140.6	65	0.01300	53.32
					70	0.05032	13.77
					75	0.06771	10.24
					80	0.08381	8.27
					85	0.2227	3.11
Tyrosinase	0.000007632	0.2440	0.04270	0.1750	50	0.01115	62.17
					55	0.01624	42.68
					60	0.04393	15.78
					65	0.05774	12.00
					70	0.1732	4.00
					75	0.2603	2.66

## Data Availability

All the data presented in the study are included in the article or Supplementary Material, further information can be provided directly by the corresponding author, Prof. Bo Wang (wb@sdust.edu.cn).

## Supplementary Materials

The following supporting information can be downloaded at: https://www.mdpi.com/article/10.3390/cimb46040230/s1, Table S1: Summary of pH-temperature optima, parameter estimates and model evaluation of respond surface methods (RSM) calculated by a quadratic regression model by two-factors and three-levels of central composite design (CCD); Table S2: Summary of experimental design matrix featuring coded variables (pH and temperature), independent variables (pH and temperature) and responses (laccase and tyrosinase activities) in relation to RSM; Figure S1: Kinetic parameters (*K*_m_ and V_max_) of laccase and tyrosinase; Figure S2: Thermal stabilities of laccase and tyrosinase; Figure S3. Supplementary figures for LC-MS analysis of products of organosolv lignin after polyphenol oxidases treatment.

## References

[1] Munk L., Andersen M.L., Meyer A.S. (2017). Direct rate assessment of laccase catalysed radical formation in lignin by electron paramagnetic resonance spectroscopy. Enzym. Microb. Technol..

[2] Kim J.Y., Lee H.W., Lee S.M., Jae J., Park Y.K. (2019). Overview of the recent advances in lignocellulose liquefaction for producing biofuels, bio-based materials and chemicals. Bioresour. Technol..

[3] Cheng K. (2021). Industrial scale lignin recovery from pulping liquors. Natural Polyphenols from Wood.

[4] Frommhagen M., Mutte S.K., Westphal A.H., Koetsier M.J., Hinz S.W.A., Visser J., Vincken J.P., Weijers D., Van Berkel W.J.H., Gruppen H. (2017). Boosting LPMO-driven lignocellulose degradation by polyphenol oxidase-activated lignin building blocks. Biotechnol. Biofuels.

[5] Huang C., Jiang X., Shen X., Hu J., Tang W., Wu X., Ragauskas A., Jameel H., Meng X., Yong Q. (2022). Lignin-enzyme interaction: A roadblock for efficient enzymatic hydrolysis of lignocellulosics. Renew. Sustain. Energy Rev..

[6] Ren S., Li C., Jiao X., Jia S., Jiang Y., Bilal M., Cui J. (2019). Recent progress in multienzymes co-immobilization and multienzyme system applications. Chem. Eng. J..

[7] Kim S., Ståhlberg J., Sandgren M., Paton R.S., Beckham G.T. (2014). Quantum mechanical calculations suggest that lytic polysaccharide monooxygenases use a copper-oxyl, oxygen-rebound mechanism. Proc. Natl. Acad. Sci. USA.

[8] Li F., Zhang J., Ma F., Chen Q., Xiao Q., Zhang X., Xie S., Yu H. (2021). Lytic polysaccharide monooxygenases promote oxidative cleavage of lignin and lignin–carbohydrate complexes during fungal degradation of lignocellulose. Environ. Microbiol..

[9] Guo X., An Y., Chai C., Sang J., Jiang L., Lu F., Dai Y., Liu F. (2020). Construction of the R17L mutant of MtC1LPMO for improved lignocellulosic biomass conversion by rational point mutation and investigation of the mechanism by molecular dynamics simulations. Bioresour. Technol..

[10] Levasseur A., Drula E., Lombard V., Coutinho P.M., Henrissat B. (2013). Expansion of the enzymatic repertoire of the CAZy database to integrate auxiliary redox enzymes. Biotechnol. Biofuels.

[11] Wu S., Tian J., Xie N., Adnan M., Wang J., Liu G. (2022). A sensitive, accurate, and high-throughput gluco-oligosaccharide oxidase-based HRP colorimetric method for assaying lytic polysaccharide monooxygenase activity. Biotechnol. Biofuels Bioprod..

[12] Hemsworth G.R., Johnston E.M., Davies G.J., Walton P.H. (2015). Lytic Polysaccharide Monooxygenases in Biomass Conversion. Trends Biotechnol..

[13] Sabbadin F., Hemsworth G.R., Ciano L., Henrissat B., Dupree P., Tryfona T., Marques R.D.S., Sweeney S.T., Besser K., Elias L. (2018). An ancient family of lytic polysaccharide monooxygenases with roles in arthropod development and biomass digestion. Nat. Commun..

[14] Janusz G., Pawlik A., Świderska-Burek U., Polak J., Sulej J., Jarosz-Wilkołazka A., Paszczyński A. (2020). Laccase Properties, Physiological Functions, and Evolution. Int. J. Mol. Sci..

[15] Perna V., Meyer A.S., Holck J., Eltis L.D., Eijsink V.G.H., Wittrup Agger J. (2020). Laccase-Catalyzed Oxidation of Lignin Induces Production of H 2 O 2. ACS Sustain. Chem. Eng..

[16] Long L., Hu Y., Sun F., Gao W., Hao Z., Yin H. (2022). Advances in lytic polysaccharide monooxygenases with the cellulose-degrading auxiliary activity family 9 to facilitate cellulose degradation for biorefinery. Int. J. Biol. Macromol..

[17] Westereng B., Cannella D., Wittrup Agger J., Jørgensen H., Larsen Andersen M., Eijsink V.G.H., Felby C. (2015). Enzymatic cellulose oxidation is linked to lignin by long-range electron transfer. Sci. Rep..

[18] Brenelli L., Squina F.M., Felby C., Cannella D. (2018). Laccase-derived lignin compounds boost cellulose oxidative enzymes AA9. Biotechnol. Biofuels.

[19] Gerbin E., Frapart Y.-M., Marcuello C., Cottyn B., Foulon L., Pernes M., Crônier D., Molinari M., Chabbert B., Ducrot P.-H. (2020). Dual Antioxidant Properties and Organic Radical Stabilization in Cellulose Nanocomposite Films Functionalized by In Situ Polymerization of Coniferyl Alcohol. Biomacromolecules.

[20] Min K., Yum T., Kim J., Woo H.M., Kim Y., Sang B.-I., Yoo Y.J., Kim Y.H., Um Y. (2017). Perspectives for biocatalytic lignin utilization: Cleaving 4-O-5 and Cα–Cβ bonds in dimeric lignin model compounds catalyzed by a promiscuous activity of tyrosinase. Biotechnol. Biofuels.

[21] Muraleedharan M.N., Zouraris D., Karantonis A., Topakas E., Sandgren M., Rova U., Christakopoulos P., Karnaouri A. (2018). Effect of lignin fractions isolated from different biomass sources on cellulose oxidation by fungal lytic polysaccharide monooxygenases. Biotechnol. Biofuels.

[22] Guo X., Sang J., Chai C., An Y., Wei Z., Zhang H., Ma L., Dai Y., Lu F., Liu F. (2020). A lytic polysaccharide monooxygenase from Myceliophthora thermophila C1 and its characterization in cleavage of glycosidic chain of cellulose. Biochem. Eng. J..

[23] Miller G.L. (1959). Use of Dinitrosalicylic Acid Reagent for Determination of Reducing Sugar. Anal. Chem..

[24] Waterhouse A., Bertoni M., Bienert S., Studer G., Tauriello G., Gumienny R., Heer F.T., de Beer T.A.P., Rempfer C., Bordoli L. (2018). SWISS-MODEL: Homology modelling of protein structures and complexes. Nucleic Acids Res..

[25] Tan T.-C., Kracher D., Gandini R., Sygmund C., Kittl R., Haltrich D., Hällberg B.M., Ludwig R., Divne C. (2015). Structural basis for cellobiose dehydrogenase action during oxidative cellulose degradation. Nat. Commun..

[26] Pettersen E.F., Goddard T.D., Huang C.C., Couch G.S., Greenblatt D.M., Meng E.C., Ferrin T.E. (2004). UCSF Chimera? A visualization system for exploratory research and analysis. J. Comput. Chem..

[27] d’Acunzo F., Galli C., Gentili P., Sergi F. (2006). Mechanistic and steric issues in the oxidation of phenolic and non-phenolic compounds by laccase or laccase-mediator systems. The case of bifunctional substrates. New J. Chem..

[28] Frommhagen M., Koetsier M.J., Westphal A.H., Visser J., Hinz S.W.A., Vincken J.-P., van Berkel W.J.H., Kabel M.A., Gruppen H. (2016). Lytic polysaccharide monooxygenases from Myceliophthora thermophila C1 differ in substrate preference and reducing agent specificity. Biotechnol. Biofuels.

[29] de Oliveira Gorgulho Silva C., Vuillemin M., Kabel M.A., van Berkel W.J.H., Meyer A.S., Agger J.W. (2023). Polyphenol Oxidase Products Are Priming Agents for LPMO Peroxygenase Activity. ChemSusChem.

[30] Sun P., Huang Z., Banerjee S., Kadowaki M.A.S., Veersma R.J., Magri S., Hilgers R., Muderspach S.J., Laurent C.V.F.P., Ludwig R. (2023). AA16 Oxidoreductases Boost Cellulose-Active AA9 Lytic Polysaccharide Monooxygenases from Myceliophthora thermophila. ACS Catal..

[31] Kracher D., Scheiblbrandner S., Felice A.K.G., Breslmayr E., Preims M., Ludwicka K., Haltrich D., Eijsink V.G.H., Ludwig R. (2016). Extracellular electron transfer systems fuel cellulose oxidative degradation. Science.

[32] Ingold C.K. (1934). Principles of an Electronic Theory of Organic Reactions. Chem. Rev..

[33] Steenken S., Neta P. (1982). One-electron redox potentials of phenols. Hydroxy- and aminophenols and related compounds of biological interest. J. Phys. Chem..

[34] Hansch C., Leo A., Taft R.W. (1991). A survey of Hammett substituent constants and resonance and field parameters. Chem. Rev..

[35] Braunschmid V., Binder K., Fuerst S., Subagia R., Danner C., Weber H., Schwaiger N., Nyanhongo G.S., Ribitsch D., Guebitz G.M. (2021). Comparison of a fungal and a bacterial laccase for lignosulfonate polymerization. Process Biochem..

[36] Boonchuay P., Techapun C., Seesuriyachan P., Chaiyaso T. (2014). Production of xylooligosaccharides from corncob using a crude thermostable endo-xylanase from Streptomyces thermovulgaris TISTR1948 and prebiotic properties. Food Sci. Biotechnol..

[37] Hahn V., Mikolasch A., Kuhlisch C., Schauer F. (2015). Laccase-mediated multi-step homo- and heteromolecular reactions of ortho-dihydroxylated aromatic compounds and mono- or diaminated substances resulting in C-C, C-O and C-N bonds. J. Mol. Catal. B Enzym..

[38] Tarrago L., Modolo C., Yemloul M., Robert V., Rousselot-Pailley P., Tron T. (2018). Controlling the polymerization of coniferyl alcohol with cyclodextrins. New J. Chem..

[39] Nemadziva B., Le Roes-Hill M., Koorbanally N., Kudanga T. (2018). Small laccase-catalyzed synthesis of a caffeic acid dimer with high antioxidant capacity. Process Biochem..

[40] Perna V., Agger J.W., Holck J., Meyer A.S. (2018). Multiple Reaction Monitoring for quantitative laccase kinetics by LC-MS. Sci. Rep..

[41] Cardullo N., Muccilli V., Tringali C. (2022). Laccase-mediated synthesis of bioactive natural products and their analogues. RSC Chem. Biol..

[42] Wang H., You S., Wang W., Zeng Y., Su R., Qi W., Wang K., He Z. (2022). Laccase-catalyzed soy protein and gallic acid complexation: Effects on conformational structures and antioxidant activity. Food Chem..

[43] Slagman S., Escorihuela J., Zuilhof H., Franssen M.C.R. (2016). Characterization of the laccase-mediated oligomerization of 4-hydroxybenzoic acid. RSC Adv..

[44] Kuusk S., Eijsink V.G.H., Väljamäe P. (2023). The “life-span” of lytic polysaccharide monooxygenases (LPMOs) correlates to the number of turnovers in the reductant peroxidase reaction. J. Biol. Chem..

[45] Yu Q., Fan L., Duan Z. (2019). Five individual polyphenols as tyrosinase inhibitors: Inhibitory activity, synergistic effect, action mechanism, and molecular docking. Food Chem..

[46] Kalia S., Thakur K., Kumar A., Celli A. (2014). Laccase-assisted surface functionalization of lignocellulosics. J. Mol. Catal. B Enzym..

[47] Moilanen U., Kellock M., Galkin S., Viikari L. (2011). The laccase-catalyzed modification of lignin for enzymatic hydrolysis. Enzym. Microb. Technol..

[48] Munk L., Sitarz A.K., Kalyani D.C., Mikkelsen J.D., Meyer A.S. (2015). Can laccases catalyze bond cleavage in lignin?. Biotechnol. Adv..

[49] Moilanen U., Kellock M., Várnai A., Andberg M., Viikari L. (2014). Mechanisms of laccase-mediator treatments improving the enzymatic hydrolysis of pre-treated spruce. Biotechnol. Biofuels.

[50] Oliva-Taravilla A., Tomás-Pejó E., Demuez M., González-Fernández C., Ballesteros M. (2016). Phenols and lignin: Key players in reducing enzymatic hydrolysis yields of steam-pretreated biomass in presence of laccase. J. Biotechnol..

[51] Jin X., Yu X., Zhu G., Zheng Z., Feng F., Zhang Z. (2016). Conditions Optimizing and Application of Laccase-mediator System (LMS) for the Laccase-catalyzed Pesticide Degradation. Sci. Rep..

[52] Zaidi K.U., Ali A.S., Ali S.A. (2014). Purification and Characterization of Melanogenic Enzyme Tyrosinase from Button Mushroom. Enzym. Res..

[53] Zeuner B., Thomsen T.B., Stringer M.A., Krogh K.B.R.M., Meyer A.S., Holck J. (2020). Comparative Characterization of Aspergillus Pectin Lyases by Discriminative Substrate Degradation Profiling. Front. Bioeng. Biotechnol..

[54] Cheng C.-M., Patel A.K., Singhania R.R., Tsai C.-H., Chen S.-Y., Chen C.-W., Dong C. (2021). Di Heterologous expression of bacterial CotA-laccase, characterization and its application for biodegradation of malachite green. Bioresour. Technol..

[55] Ramírez-Cavazos L.I., Junghanns C., Ornelas-Soto N., Cárdenas-Chávez D.L., Hernández-Luna C., Demarche P., Enaud E., García-Morales R., Agathos S.N., Parra R. (2014). Purification and characterization of two thermostable laccases from Pycnoporus sanguineus and potential role in degradation of endocrine disrupting chemicals. J. Mol. Catal. B Enzym..

[56] Zynek K., Bryjak J., Polakovič M. (2010). Effect of separation on thermal stability of tyrosinase from Agaricus bisporus. J. Mol. Catal. B Enzym..

