# Supplementary material for: Effects of Lignin-Diverted Reductant with Polyphenol Oxidases on Cellulose Degradation by Wild and Mutant Types of Lytic Polysaccharide Monooxygenase
Source: Curr Issues Mol Biol. 2024 Apr 21;46(4):3694–712. doi: 10.3390/cimb46040230 (PMC11049000; doi:10.3390/cimb46040230)

Supplementary Information for

Effects of Lignin-Diverted Reductant with Polyphenol Oxidases on Cellulose Degradation by Wild and Mutant Types of Lytic Polysaccharide Monooxygenase

Kai Li, Yuan Wang, Xiao Guo and Bo Wang *

College of Chemical and Biological Engineering, Shandong University of Science and Technology, Qingdao 266590, China

* Correspondence: wb@sdust.edu.cn

Table S1 Summary of pH-temperature optima, parameter estimates and model evaluation of respond surface methods (RSM) calculated by a quadratic regression model by two-factors and three-levels of central composite design (CCD). The quadratic equation of the models is absorbance = intercept + β_1 T + β_2 T^2 + β_3 pH + β_4 pH^2 + pH*T, and the parameters of equation (β-values) are shown above. The model evaluation of R^2 exhibits the correlation between fitting model and results. The *p*-value is used for representing the lack of fit, where $p < 0.05$ means a significant lack of fit. The *italic* value represents an insignificant ($p > 0.05$) factor within the model.

Name	pH	Temperature (℃)	Parameter Estimates						Evaluation	
			Intercept	T	T^2	pH	pH^2	pH*T	R^2	Lack of Fit
Laccase	5.19	54	0.005907	0.0010681	-0.001164	0.0003911	-0.001272	0.0007818	0.9505	<0.0001
Tyrosinase	7.01	35	0.1014321	0.009775	-0.014264	*-0.000575*	-0.022564	*0.0027875*	0.863	0.0325

Table S2 Summary of experimental design matrix featuring coded variables (pH and temperature), independent variables (pH and temperature) and responses (laccase and tyrosinase activities) in relation to RSM.

Run	Coded Variables		Independent Variables		Laccase Activity (ΔAbs/min)		Independent Variables		Tyrosinase Activity (ΔAbs)	
Order	pH	Temperature	pH	Temperature (°C)	Experimental	Predicted	pH	Temperature (°C)	Experimental	Predicted
1	-1	-1	3.2	30	0.002945	0.002794	5.5	15	0.0495	0.0582
2	-1	-1	3.2	30	0.003115	0.002794	5.5	15	0.0560	0.0582
3	0	-1	4.7	30	0.003466	0.003675	7.0	15	0.0781	0.0774
4	0	-1	4.7	30	0.003463	0.003675	7.0	15	0.0909	0.0774
5	1	-1	6.2	30	0.002003	0.002012	8.5	15	0.0482	0.0515
6	1	-1	6.2	30	0.001969	0.002012	8.5	15	0.0515	0.0515
7	-1	0	3.2	45	0.003664	0.004244	5.5	30	0.0751	0.0795
8	-1	0	3.2	45	0.003705	0.004244	5.5	30	0.0907	0.0795
9	0	0	4.7	45	0.006147	0.005907	7.0	30	0.0964	0.1015
10	0	0	4.7	45	0.005908	0.005907	7.0	30	0.0967	0.1015
11	0	0	4.7	45	0.006314	0.005907	7.0	30	0.1068	0.1015
12	0	0	4.7	45	0.006445	0.005907	7.0	30	0.1077	0.1015
13	1	0	6.2	45	0.004989	0.005026	8.5	30	0.0717	0.0783
14	1	0	6.2	45	0.004994	0.005026	8.5	30	0.0763	0.0783
15	-1	1	3.2	60	0.003740	0.003366	5.5	45	0.0710	0.0722
16	-1	1	3.2	60	0.003638	0.003366	5.5	45	0.0775	0.0722
17	0	1	4.7	60	0.005491	0.005811	7.0	45	0.0879	0.0970
18	0	1	4.7	60	0.005365	0.005811	7.0	45	0.0900	0.0970
19	1	1	6.2	60	0.005619	0.005712	8.5	45	0.0818	0.0766
20	1	1	6.2	60	0.005926	0.005712	8.5	45	0.0833	0.0766

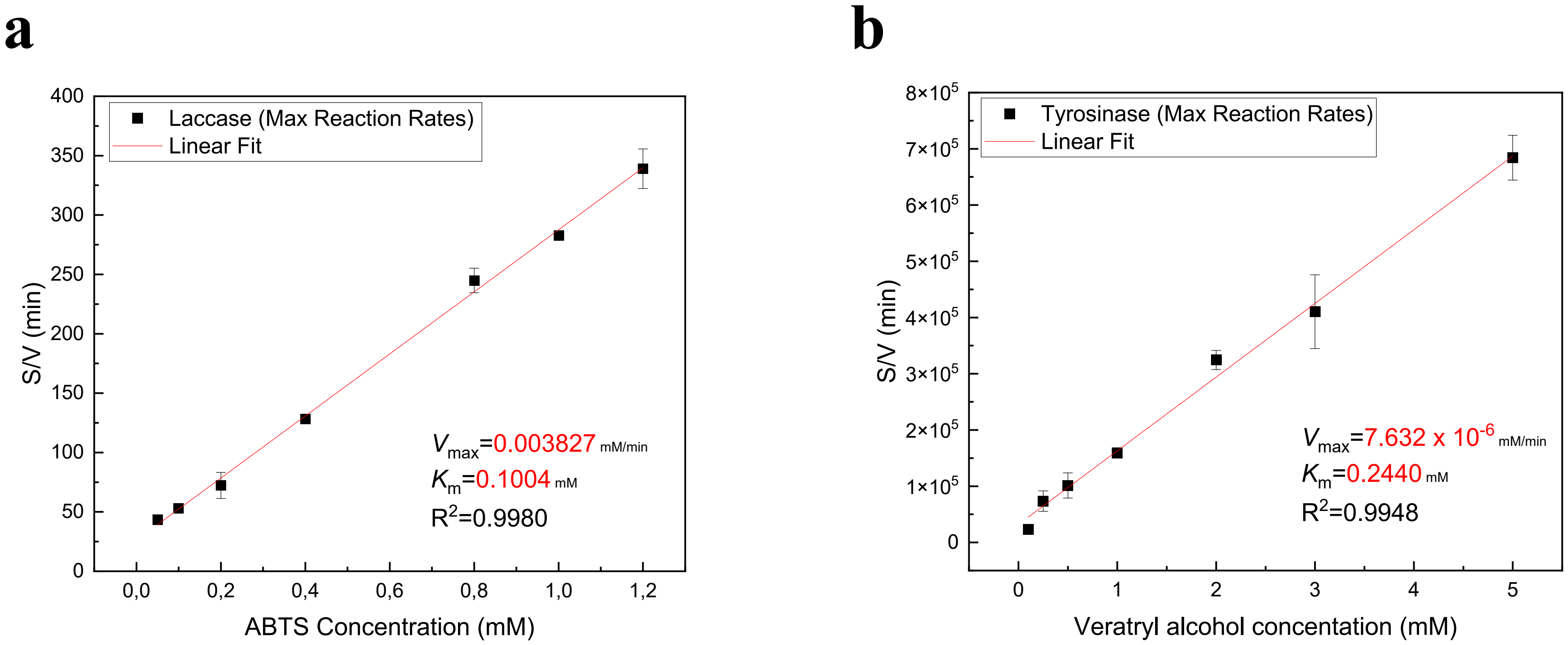

Figure S1 Kinetic parameters (K_m and V_{max}) of (**a**) laccase and (**b**) tyrosinase. The linear integrate of Hanes-Woolf plot is used for the calculation of kinetic constants.

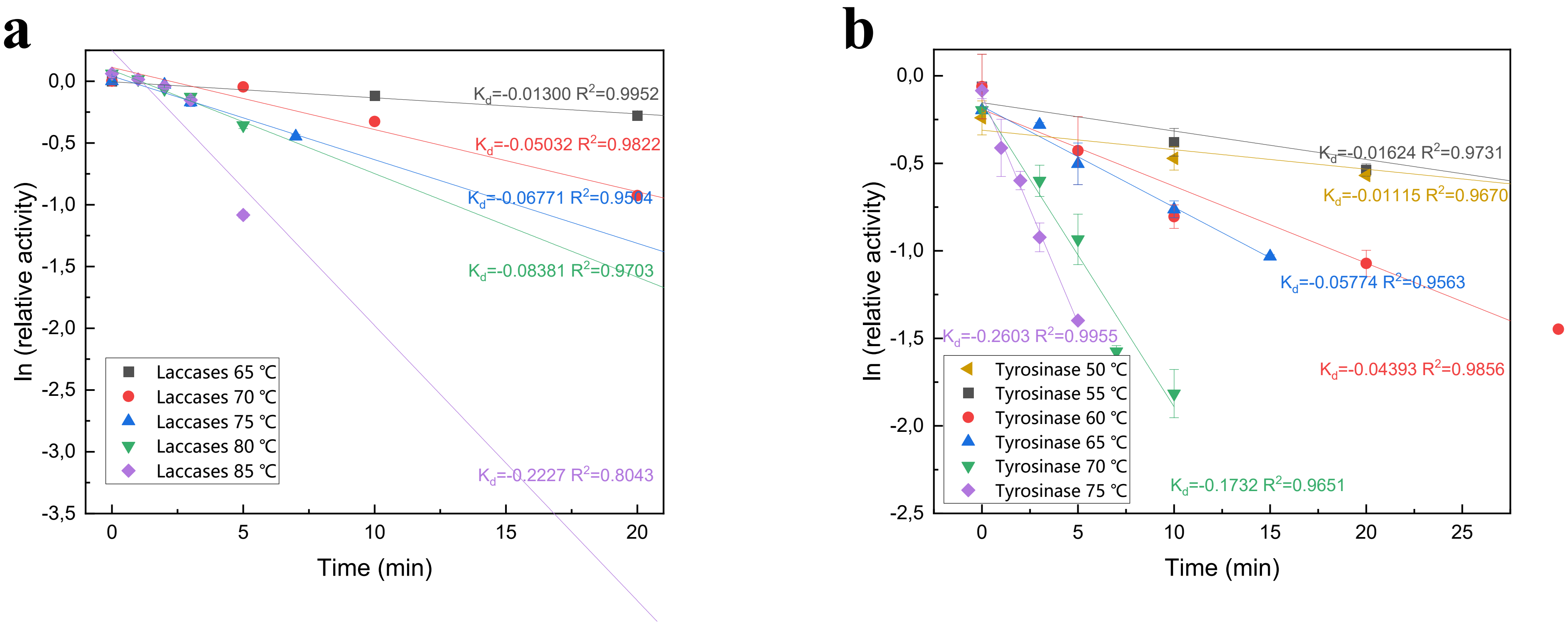

Figure S2 Thermal stabilities of (**a**) laccase and (**b**) tyrosinase. The values of K_d and $t_{1/2}$ are calculated by linear fit of plot, which draws using the ln (relative avtivity) as the Y-axis and the time as the X-axis (minutes). The selection of enzymatic reaction time is based on the experiment results in different temperatures.

Figure S3. Supplementary figures for LC-MS analysis of products of organosolv lignin after polyphenol oxidases treatment (small lignin-derived phenolic compounds): (**a**) LK-1 R: reference without polyphenol oxidases, (**b**) LK-2: the product of organosolv lignin by laccase treatment and (**c**) LK-3: the product of organosolv lignin by tyrosinase treatment. All the documents for the results of LC-MS analysis are attached.

Openlynx Report -

Sample: 642 Vial:1:B,3 ID:
File:LK-1 R Date:03-Aug-2023 Time:12:27:58
Description:Default file

Printed: Thu Aug 03 15:04:15 2023

Sample Report:

Sample 642 Vial 1:B,3 ID File LK-1 R Date 03-Aug-2023 Time 12:27:58 Description Default file

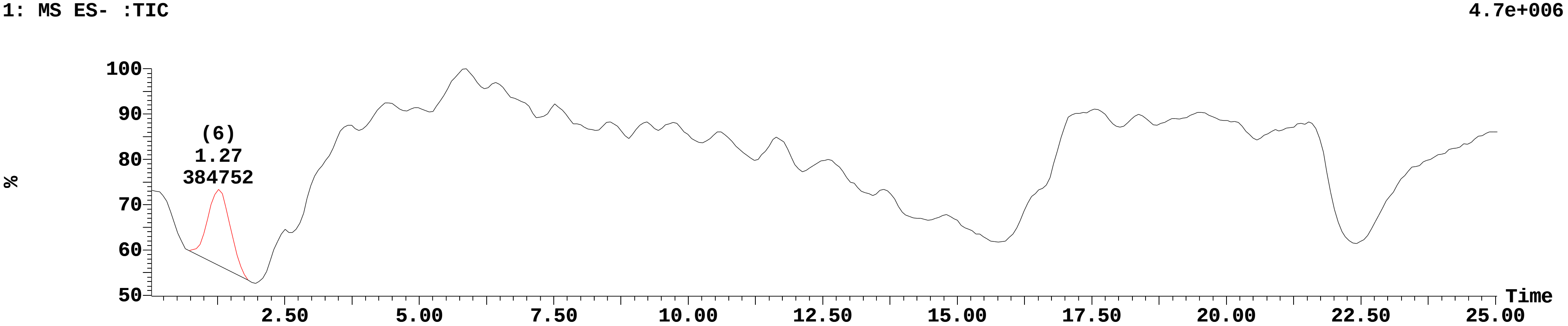

Peak Number	Compound	Time	AreaAbs	Area %Total	Width	Height	Mass Found
6		1.27	4e+005	100.00	1	8e+005	

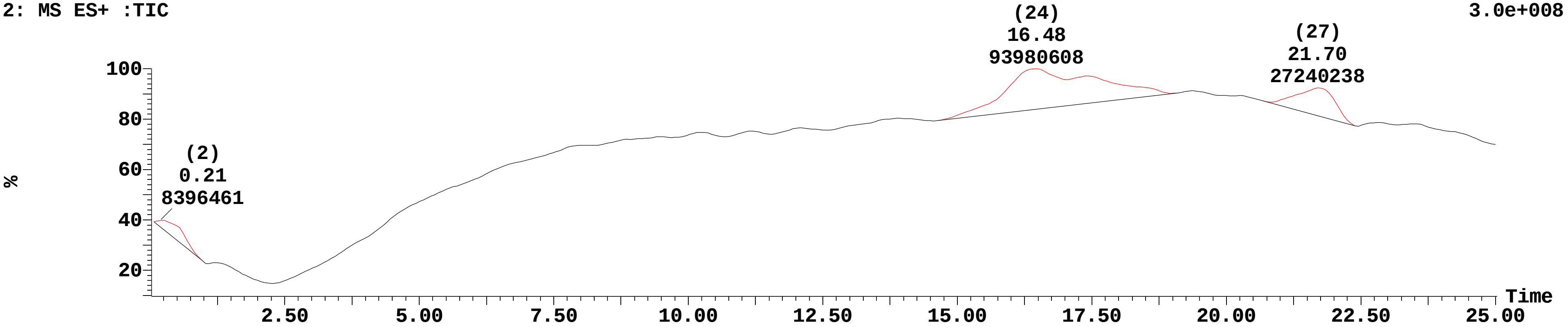

Peak Number	Compound	Time	AreaAbs	Area %Total	Width	Height	Mass Found
2		0.21	8e+006	6.48	1	9e+006	
24		16.48	9e+007	72.51	4	5e+007	
27		21.70	3e+007	21.02	2	3e+007	

Sample: 642 Vial:1:B,3 ID:
File:LK-1 R Date:03-Aug-2023 Time:12:27:58
Description:Default file

Printed: Thu Aug 03 15:04:15 2023

Sample Report (continued):

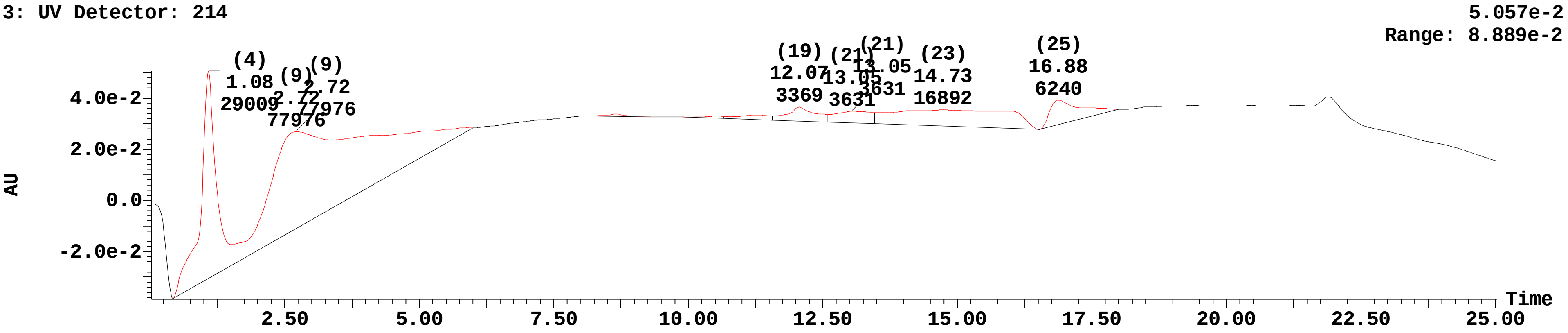

Peak Number	Compound	Time	AreaAbs	Area %Total	Width	Height	Mass Found
4		1.08	3e+004	20.86	1	8e+004	
9		5.90	8e+004	56.06	4	1e+003	
12		8.65	4e+002	0.27	1	1e+003	
15		10.52	3e+002	0.25	1	9e+002	
18		11.27	1e+003	0.91	1	2e+003	
19		12.07	3e+003	2.42	1	5e+003	
21		13.05	4e+003	2.61	1	5e+003	
23		14.73	2e+004	12.14	3	6e+003	
25		16.88	6e+003	4.49	1	1e+004	

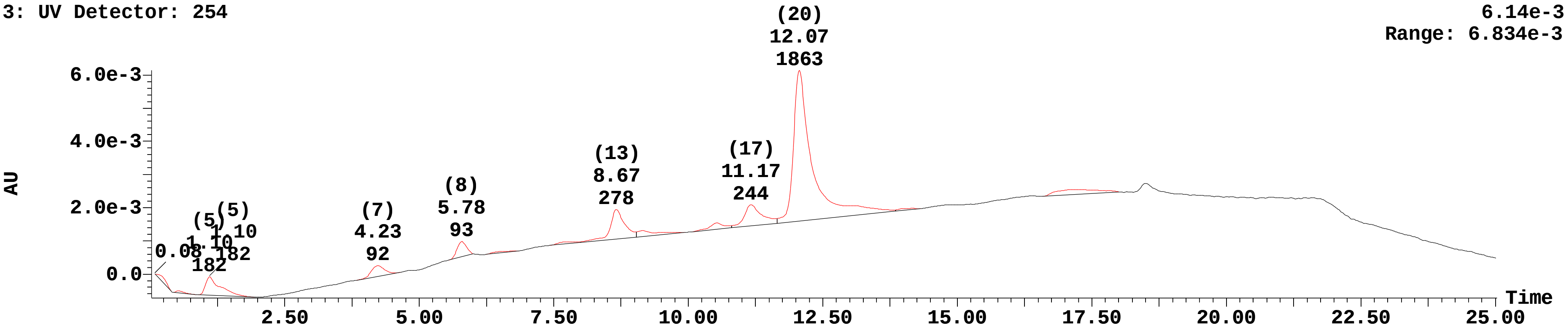

Sample: 642 Vial:1:B,3 ID:
File:LK-1 R Date:03-Aug-2023 Time:12:27:58
Description:Default file

Printed: Thu Aug 03 15:04:15 2023

Sample Report (continued):

Peak Number	Compound	Time	AreaAbs	Area %Total	Width	Height	Mass Found
1		0.08	3e+001	0.94	0		
3		0.53	1e+001	0.35	0	6e+001	
5		1.10	2e+002	5.83	1	6e+002	
7		4.23	9e+001	2.96	1	3e+002	
8		5.78	9e+001	3.00	0	5e+002	
10		6.85	2e+001	0.49	1		
11		7.83	2e+001	0.71	1	5e+001	
13		8.67	3e+002	8.94	1	9e+002	
14		9.15	7e+001	2.26	1	2e+002	
16		10.53	6e+001	1.99	1	2e+002	
17		11.17	2e+002	7.84	1	6e+002	
20		12.07	2e+003	59.82	2	5e+003	
22		14.17	2e+001	0.57	0	4e+001	
26		17.28	1e+002	4.30	1	1e+002	

Peak ID	Compound	Time	Mass Found
1		0.08	

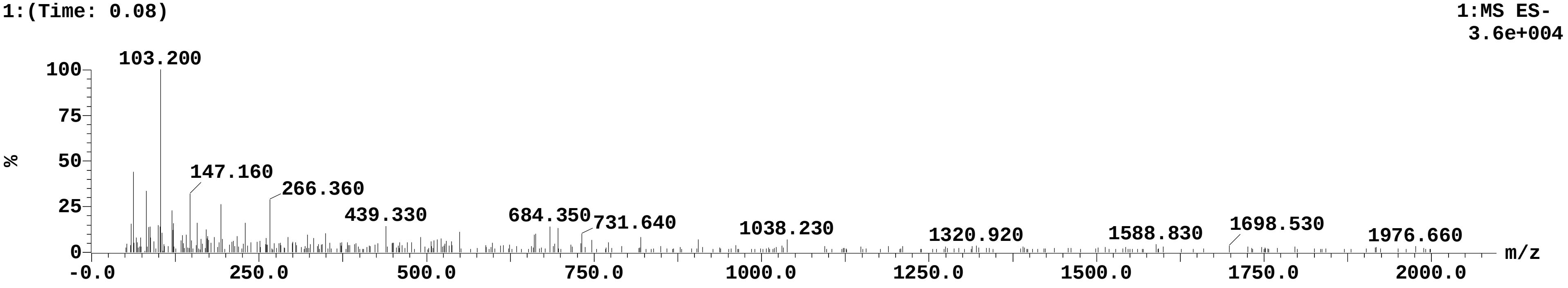

Sample: 642 Vial:1:B,3 ID:
File:LK-1 R Date:03-Aug-2023 Time:12:27:58
Description:Default file

Printed: Thu Aug 03 15:04:15 2023

Sample Report (continued):

Peak ID	Compound	Time	Mass Found
3		0.53	

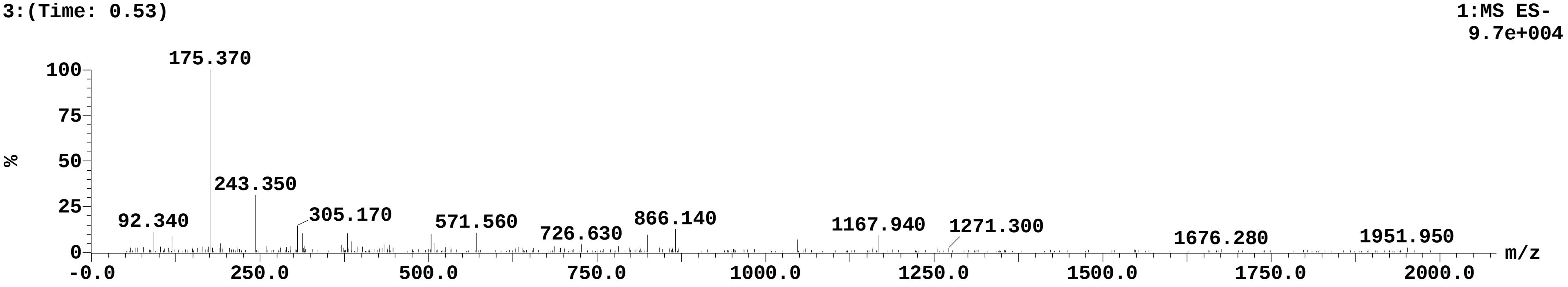

Peak ID	Compound	Time	Mass Found
4		1.08	

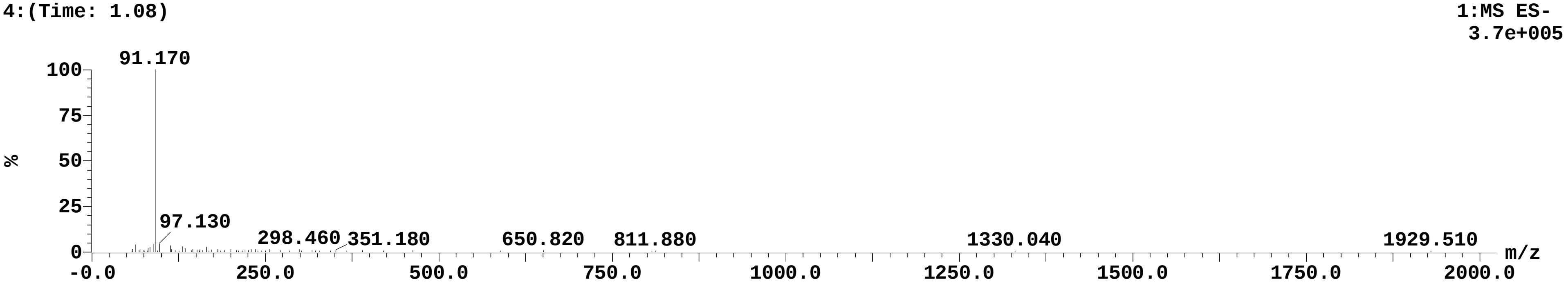

Sample Report (continued):

Peak ID	Compound	Time	Mass Found
5		1.10	

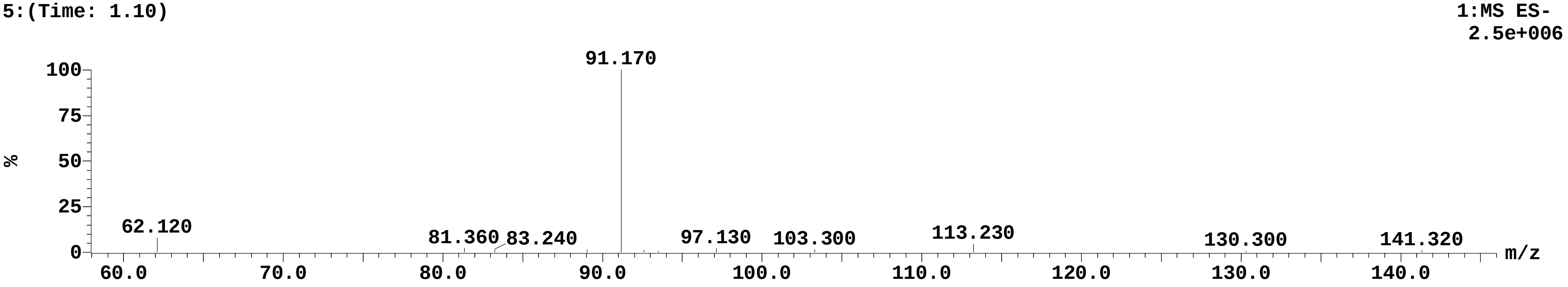

Peak ID	Compound	Time	Mass Found
6		1.27	

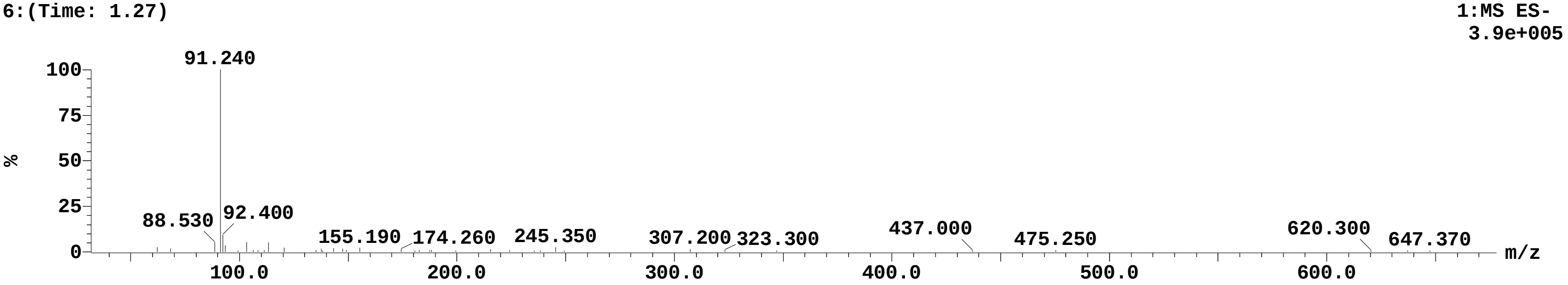

Sample: 642 Vial:1:B,3 ID:
File:LK-1 R Date:03-Aug-2023 Time:12:27:58
Description:Default file

Printed: Thu Aug 03 15:04:15 2023

Sample Report (continued):

Peak ID	Compound	Time	Mass Found
7		4.23	

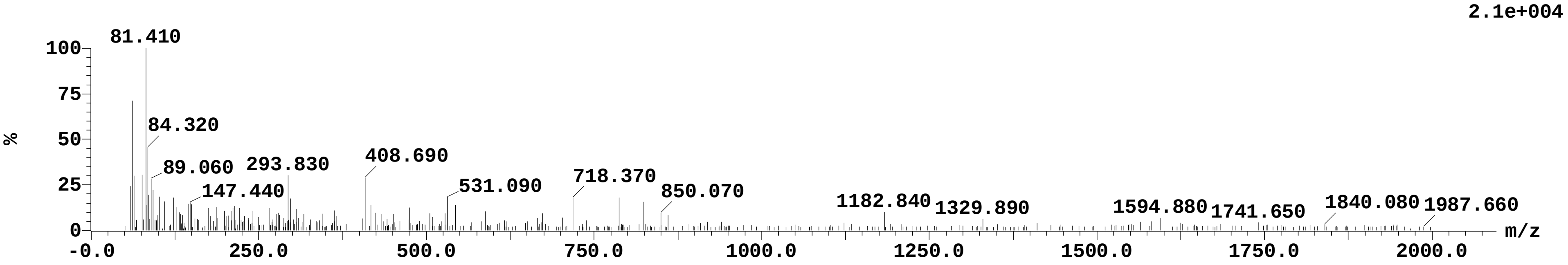

Peak ID	Compound	Time	Mass Found
8		5.78	

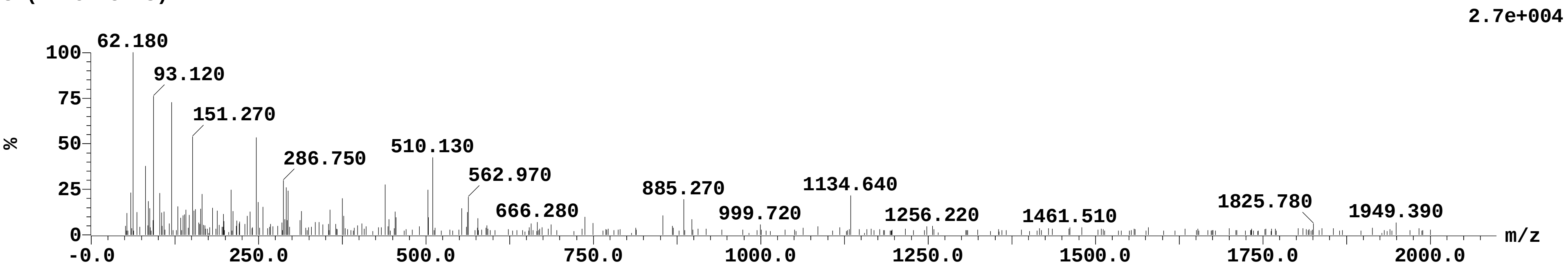

Sample: 642 | Vial:1:B,3 | ID:
File:LK-1 R | Date:03-Aug-2023 | Time:12:27:58
Description:Default file

Printed: Thu Aug 03 15:04:15 2023

Sample Report (continued):

Peak ID	Compound	Time	Mass Found
9		5.90	

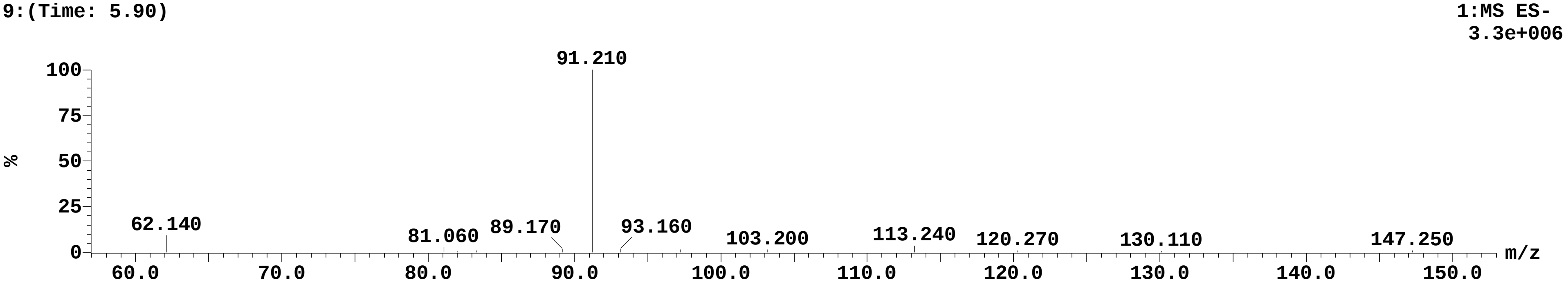

Peak ID	Compound	Time	Mass Found
10		6.85	

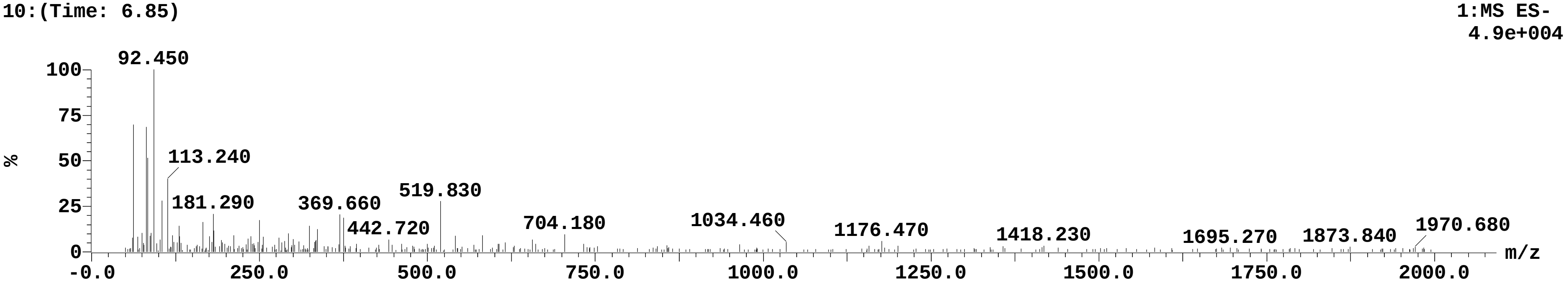

Sample: 642 Vial:1:B,3 ID:
File:LK-1 R Date:03-Aug-2023 Time:12:27:58
Description:Default file

Printed: Thu Aug 03 15:04:15 2023

Sample Report (continued):

Peak ID	Compound	Time	Mass Found
11		7.83	

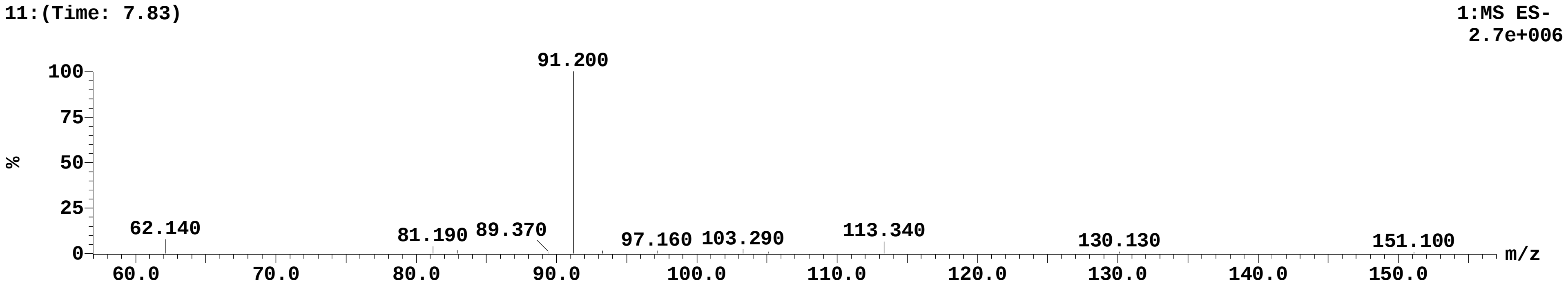

Peak ID	Compound	Time	Mass Found
12		8.65	

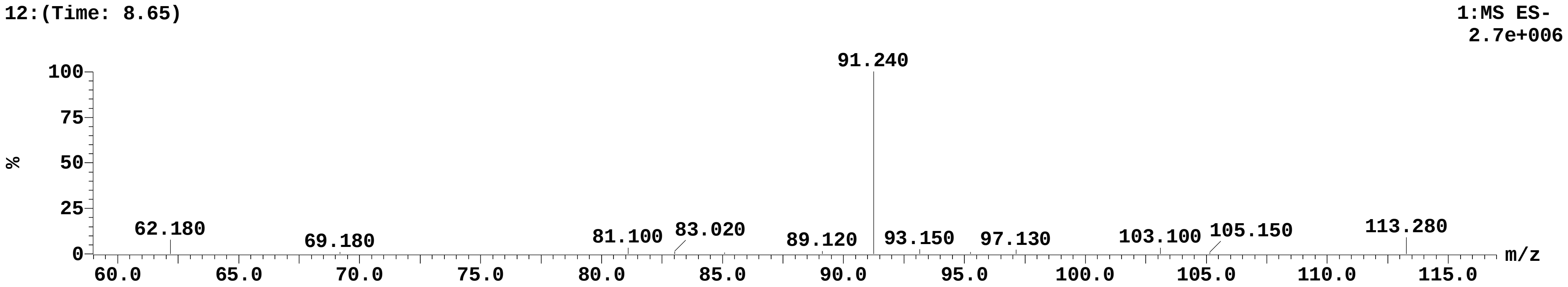

Sample: 642 Vial:1:B,3 ID:
File:LK-1 R Date:03-Aug-2023 Time:12:27:58
Description:Default file

Printed: Thu Aug 03 15:04:15 2023

Sample Report (continued):

Peak ID	Compound	Time	Mass Found
13		8.67	

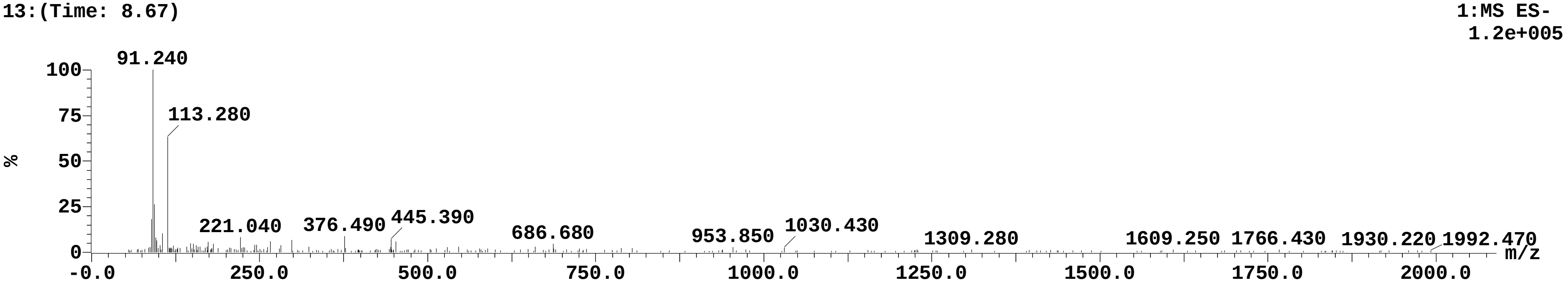

Peak ID	Compound	Time	Mass Found
14		9.15	

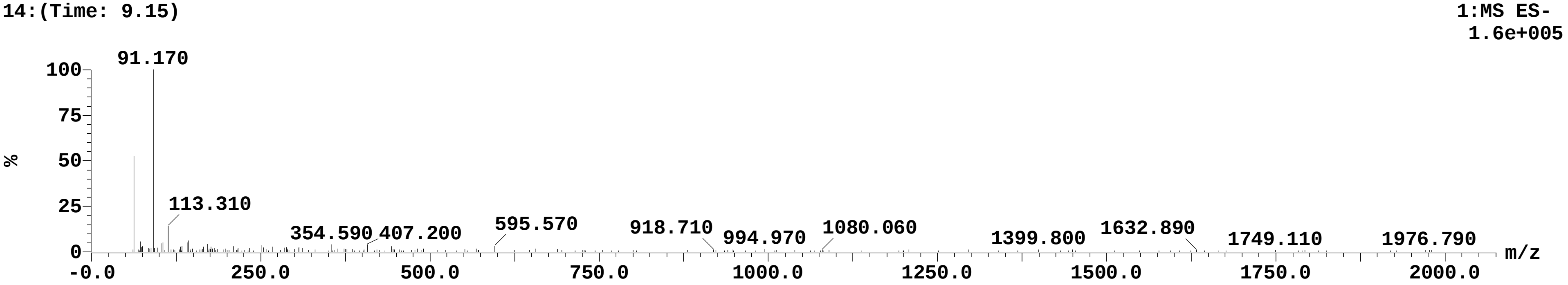

Sample: 642 Vial:1:B,3 ID:
File:LK-1 R Date:03-Aug-2023 Time:12:27:58
Description:Default file

Printed: Thu Aug 03 15:04:15 2023

Sample Report (continued):

Peak ID	Compound	Time	Mass Found
15		10.52	

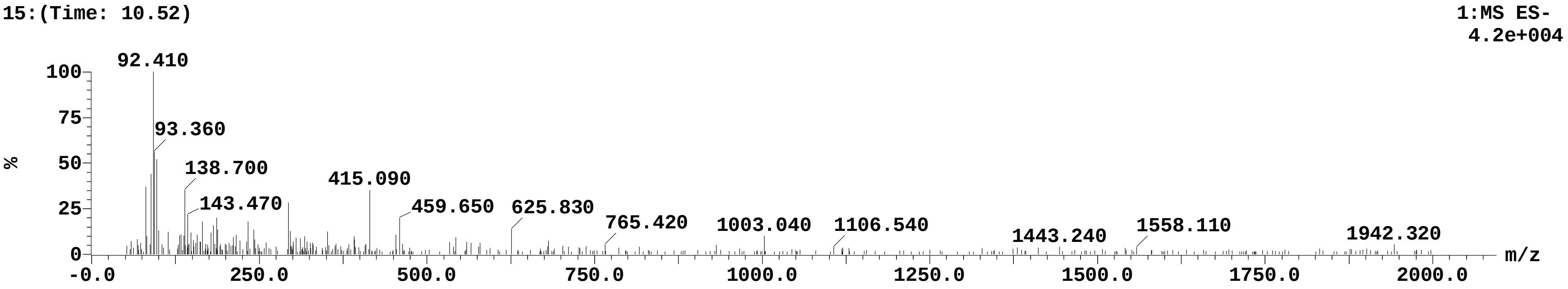

Peak ID	Compound	Time	Mass Found
16		10.53	

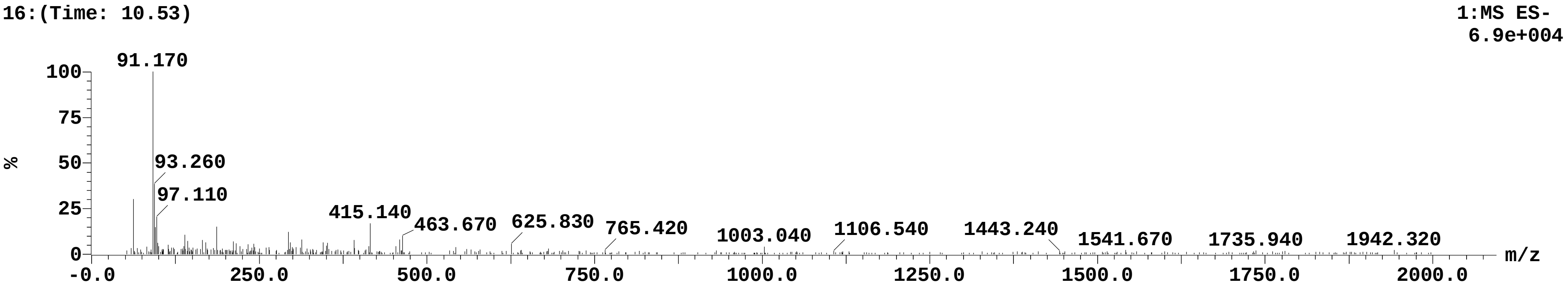

Sample: 642 Vial:1:B,3 ID:
File:LK-1 R Date:03-Aug-2023 Time:12:27:58
Description:Default file

Printed: Thu Aug 03 15:04:15 2023

Sample Report (continued):

Peak ID	Compound	Time	Mass Found
17		11.17	

17:(Time: 11.17)

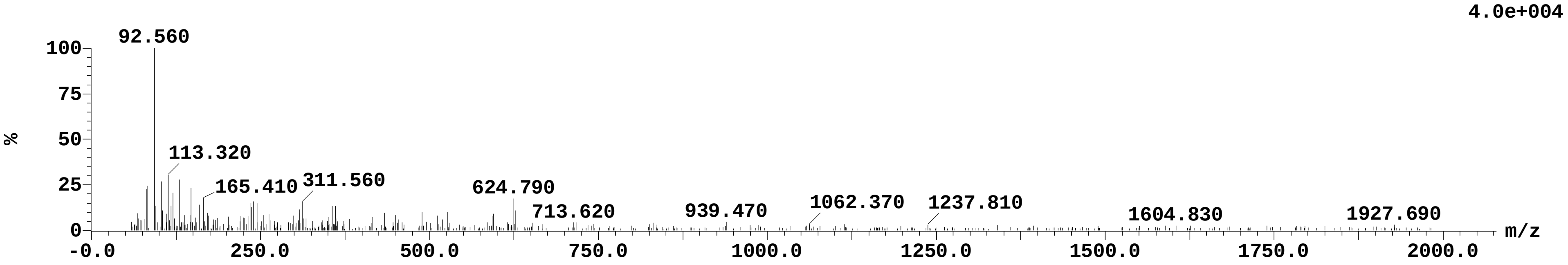

Peak ID	Compound	Time	Mass Found
18		11.27	

18:(Time: 11.27)

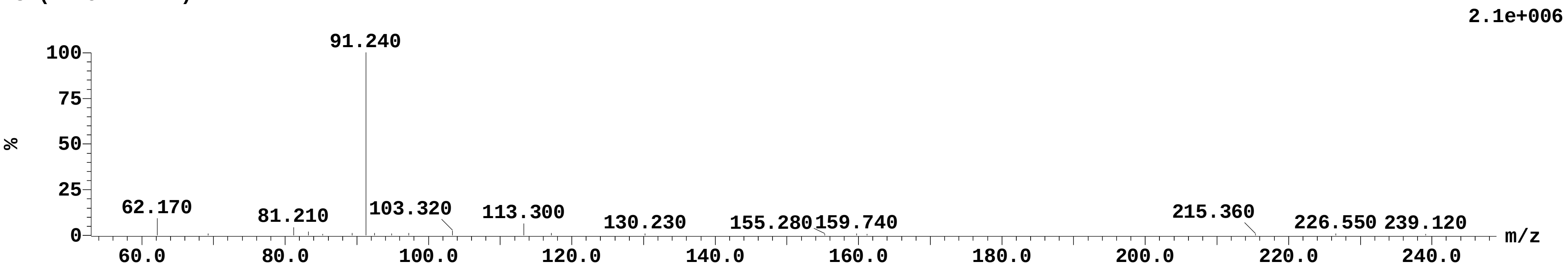

Sample Report (continued):

Peak ID	Compound	Time	Mass Found
19		12.07	

19:(Time: 12.07) 1:MS ES- 6.5e+004

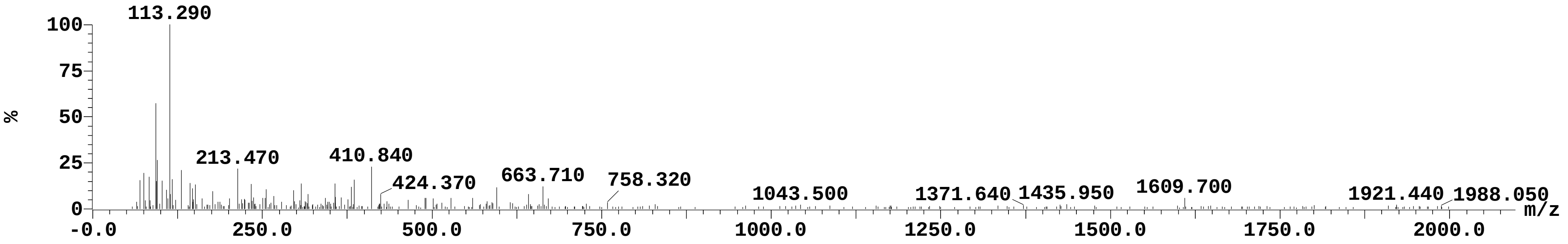

Peak ID	Compound	Time	Mass Found
20		12.07	

20:(Time: 12.07) 1:MS ES- 6.5e+004

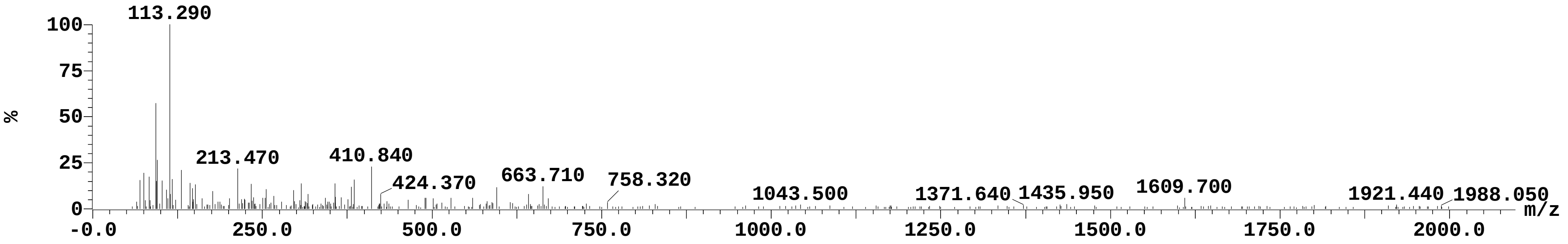

Sample: 642 Vial:1:B,3 ID:
File:LK-1 R Date:03-Aug-2023 Time:12:27:58
Description:Default file

Printed: Thu Aug 03 15:04:15 2023

Sample Report (continued):

Peak ID	Compound	Time	Mass Found
21		13.05	

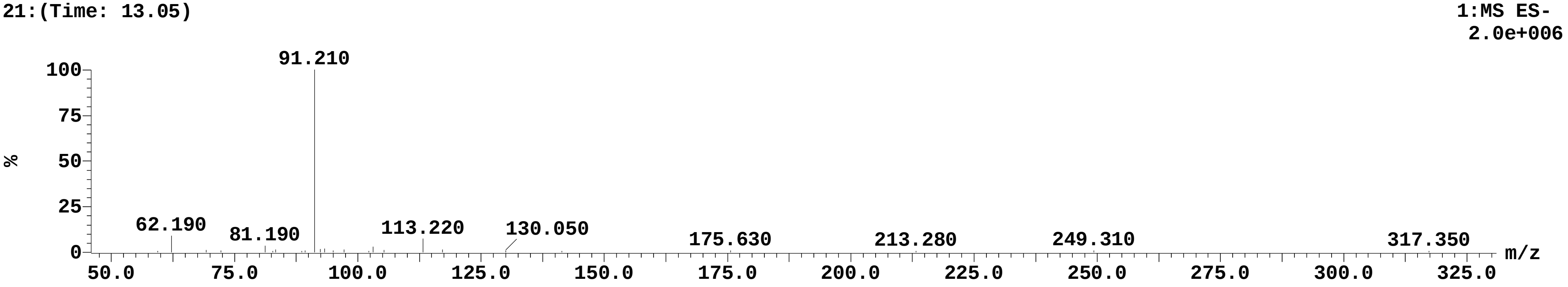

Peak ID	Compound	Time	Mass Found
22		14.17	

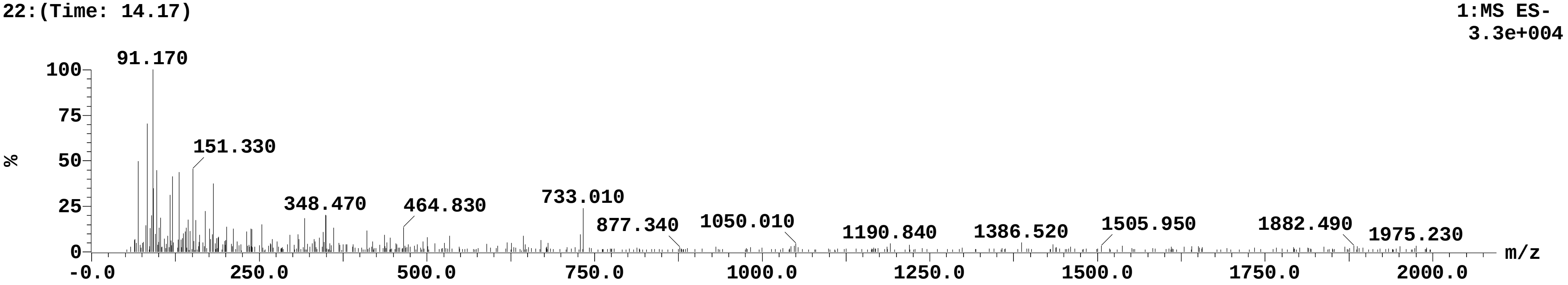

Sample: 642 Vial:1:B,3 ID:
File:LK-1 R Date:03-Aug-2023 Time:12:27:58
Description:Default file

Printed: Thu Aug 03 15:04:15 2023

Sample Report (continued):

Peak ID	Compound	Time	Mass Found
23		14.73	

23:(Time: 14.73) 1:MS ES- 2.0e+005

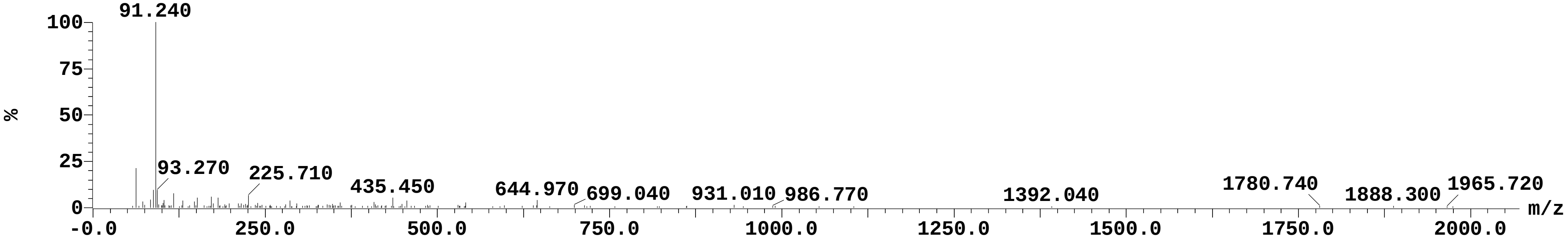

Peak ID	Compound	Time	Mass Found
25		16.88	

25:(Time: 16.88) 1:MS ES- 2.0e+005

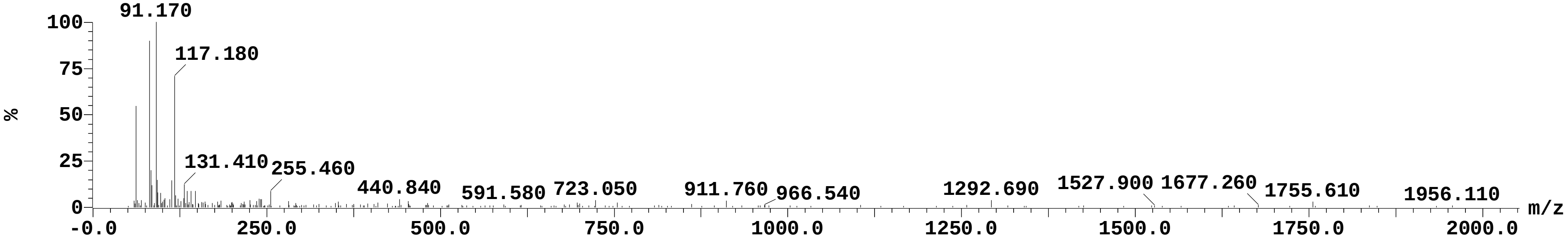

Sample: 642 Vial:1:B,3 ID:
File:LK-1 R Date:03-Aug-2023 Time:12:27:58
Description:Default file

Printed: Thu Aug 03 15:04:15 2023

Sample Report (continued):

Peak ID	Compound	Time	Mass Found
26		17.28	

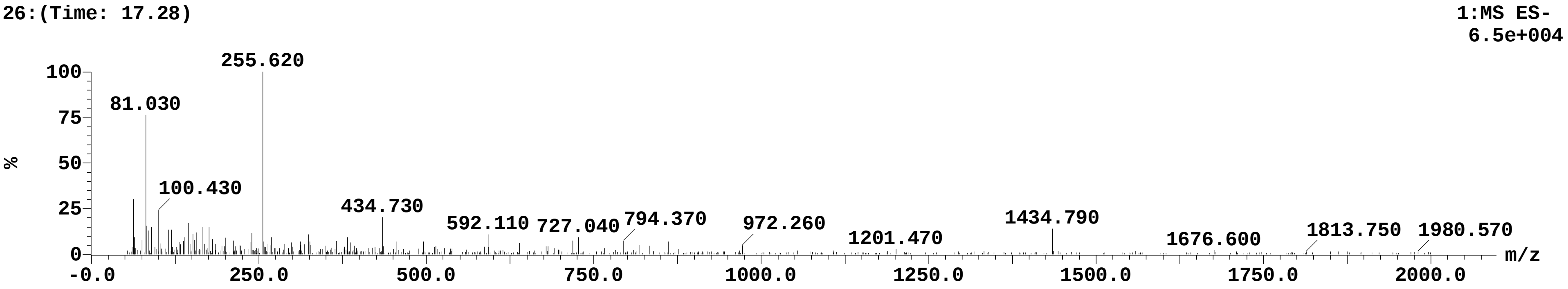

Peak ID	Compound	Time	Mass Found
1		0.08	

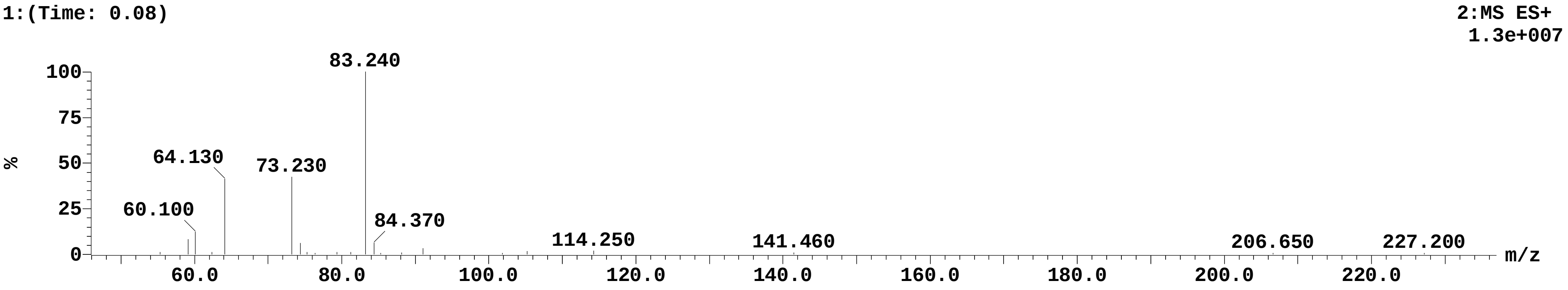

Sample Report (continued):

Peak ID	Compound	Time	Mass Found
2		0.21	

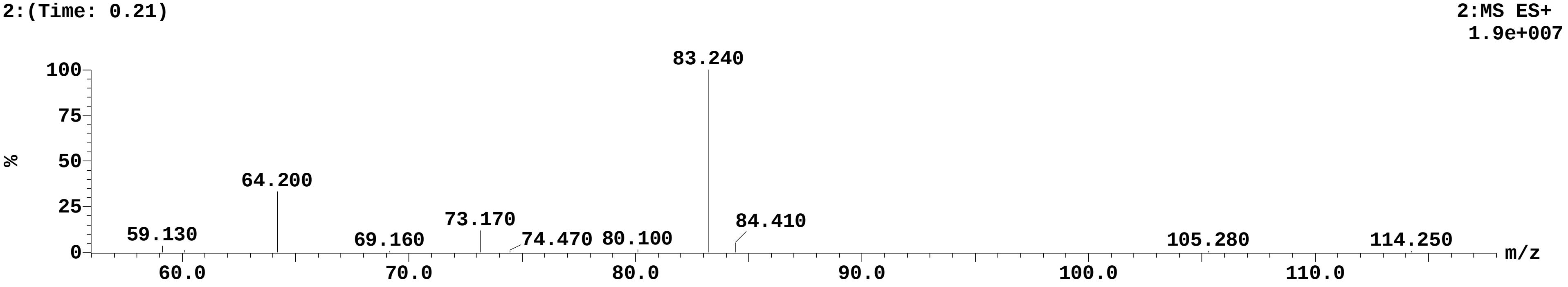

Peak ID	Compound	Time	Mass Found
3		0.53	

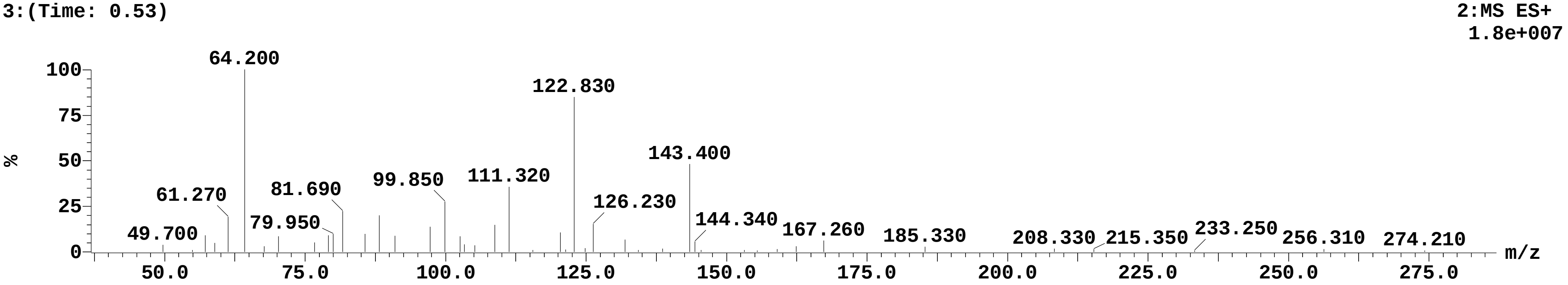

Sample Report (continued):

Peak ID	Compound	Time	Mass Found
4		1.08	

4:(Time: 1.08)

2:MS ES+
3.0e+006

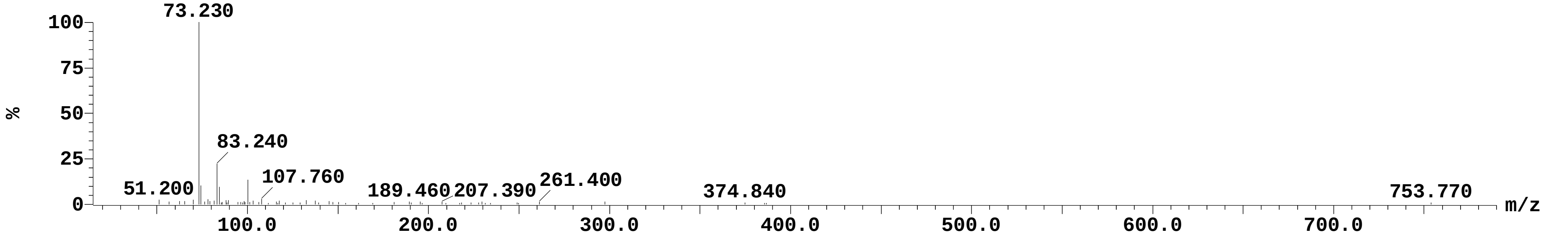

Peak ID	Compound	Time	Mass Found
5		1.10	

5:(Time: 1.10)

2:MS ES+
3.0e+006

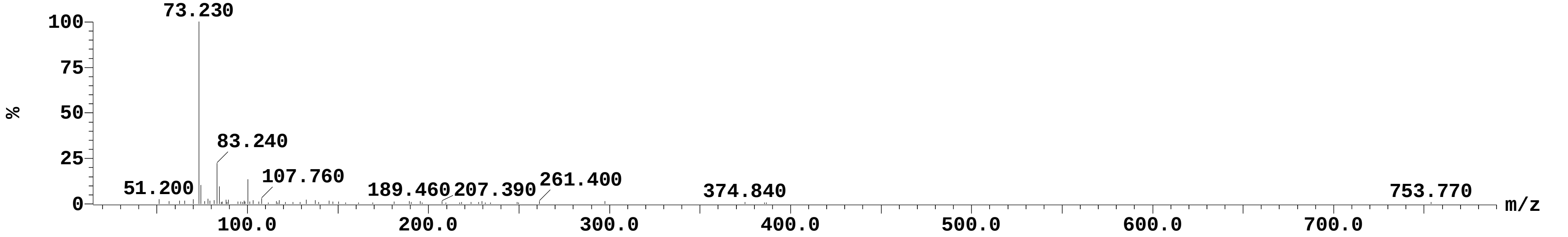

Sample: 642 Vial:1:B,3 ID:
File:LK-1 R Date:03-Aug-2023 Time:12:27:58
Description:Default file

Printed: Thu Aug 03 15:04:15 2023

Sample Report (continued):

Peak ID	Compound	Time	Mass Found
7		4.23	

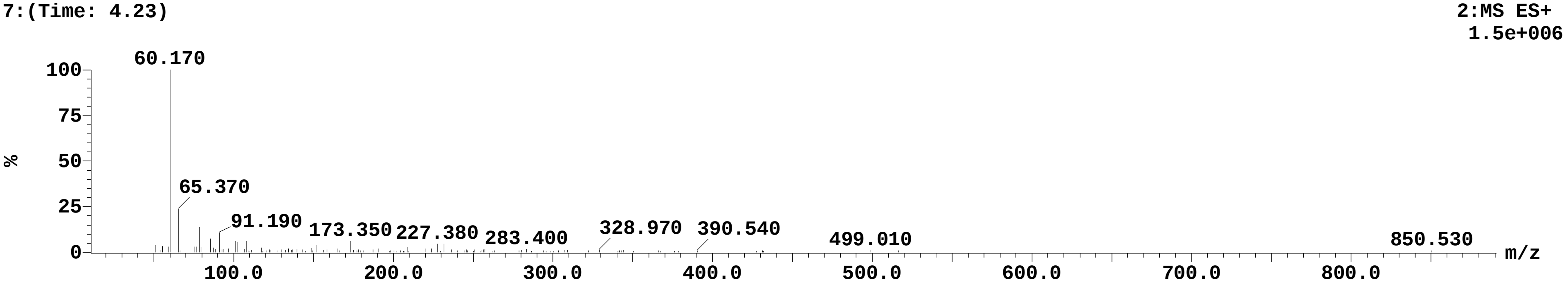

Peak ID	Compound	Time	Mass Found
8		5.78	

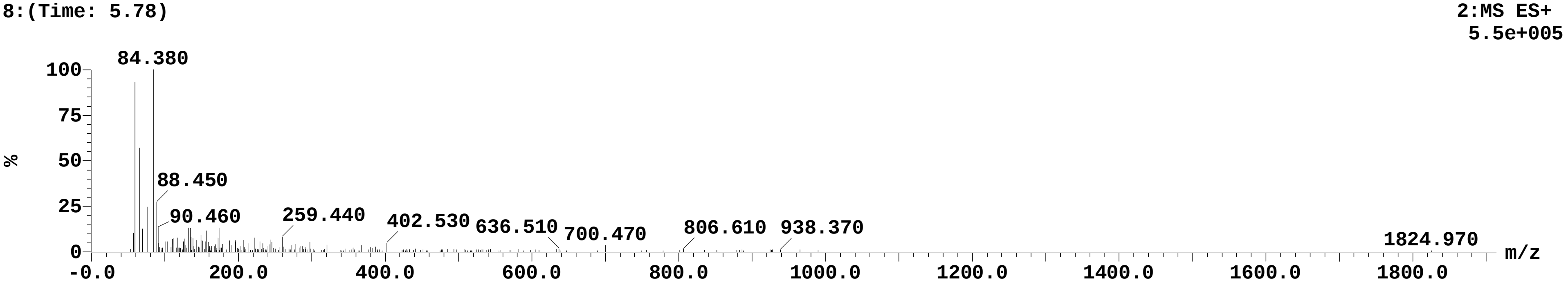

Sample: 642 | Vial:1:B,3 | ID:
File:LK-1 R | Date:03-Aug-2023 | Time:12:27:58
Description:Default file

Printed: Thu Aug 03 15:04:15 2023

Sample Report (continued):

Peak ID	Compound	Time	Mass Found
9		5.90	

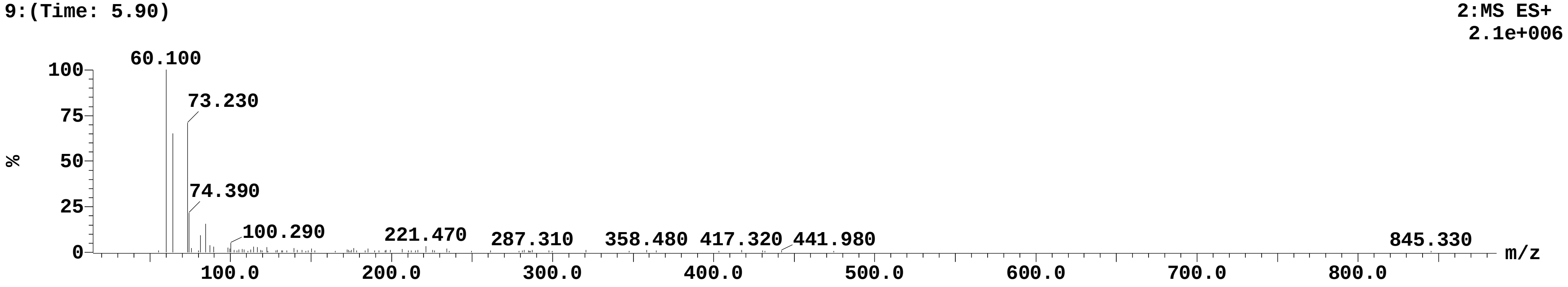

Peak ID	Compound	Time	Mass Found
10		6.85	

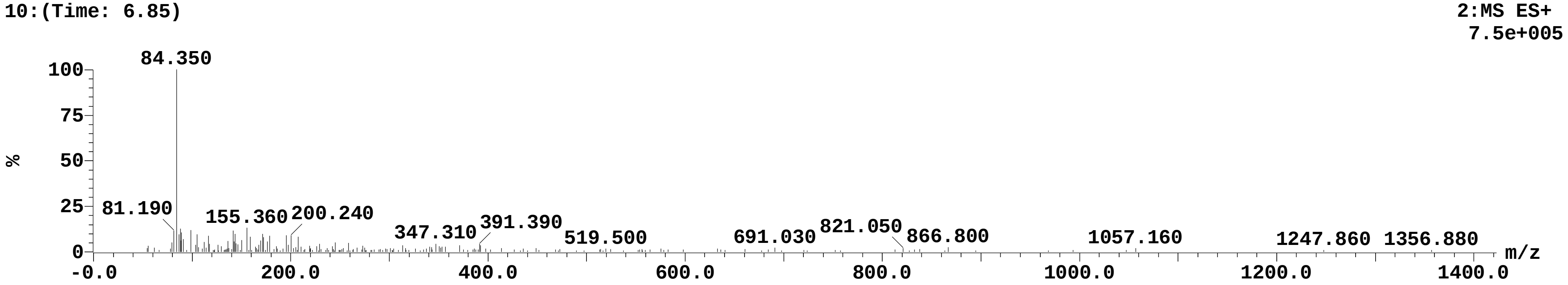

Sample: 642 Vial:1:B,3 ID:
File:LK-1 R Date:03-Aug-2023 Time:12:27:58
Description:Default file

Printed: Thu Aug 03 15:04:15 2023

Sample Report (continued):

Peak ID	Compound	Time	Mass Found
11		7.83	

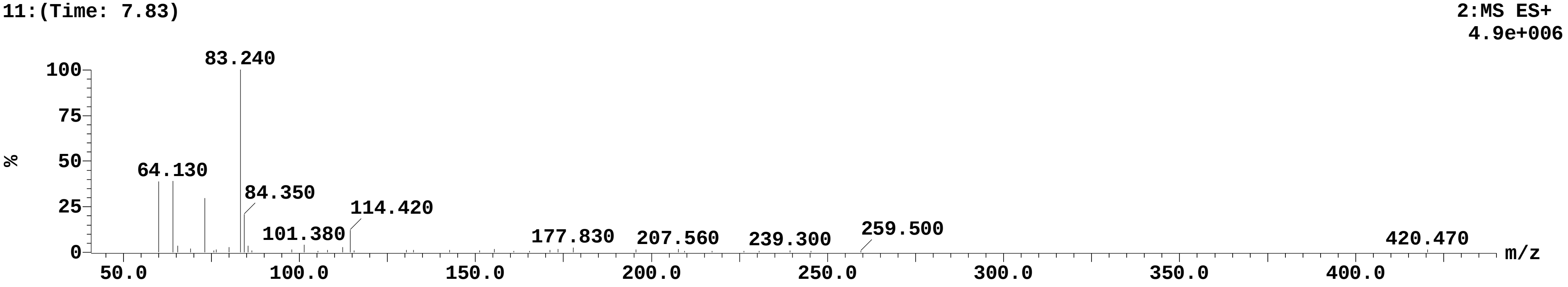

Peak ID	Compound	Time	Mass Found
12		8.65	

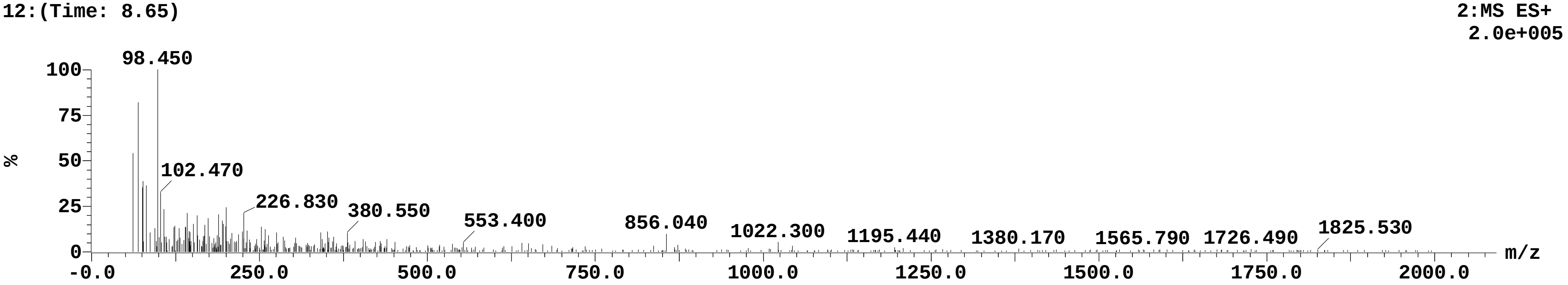

Sample: 642 Vial:1:B,3 ID:
File:LK-1 R Date:03-Aug-2023 Time:12:27:58
Description:Default file

Printed: Thu Aug 03 15:04:15 2023

Sample Report (continued):

Peak ID	Compound	Time	Mass Found
13		8.67	

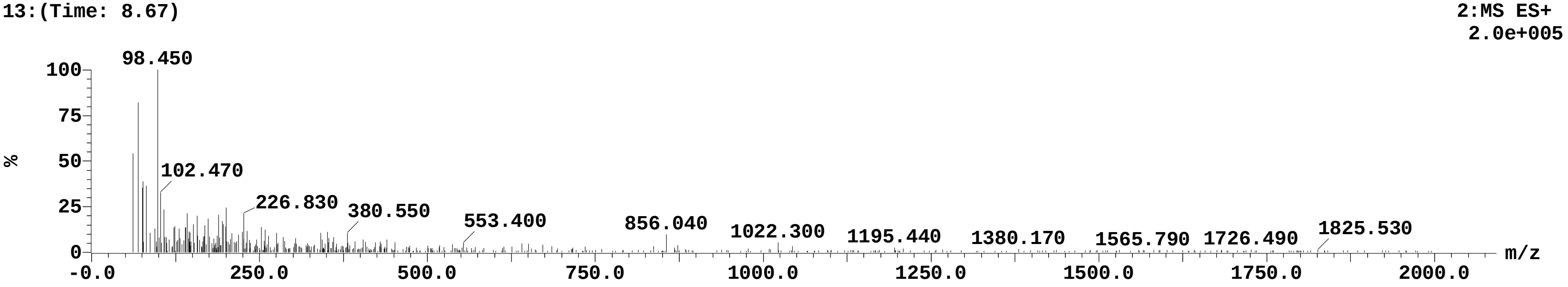

Peak ID	Compound	Time	Mass Found
14		9.15	

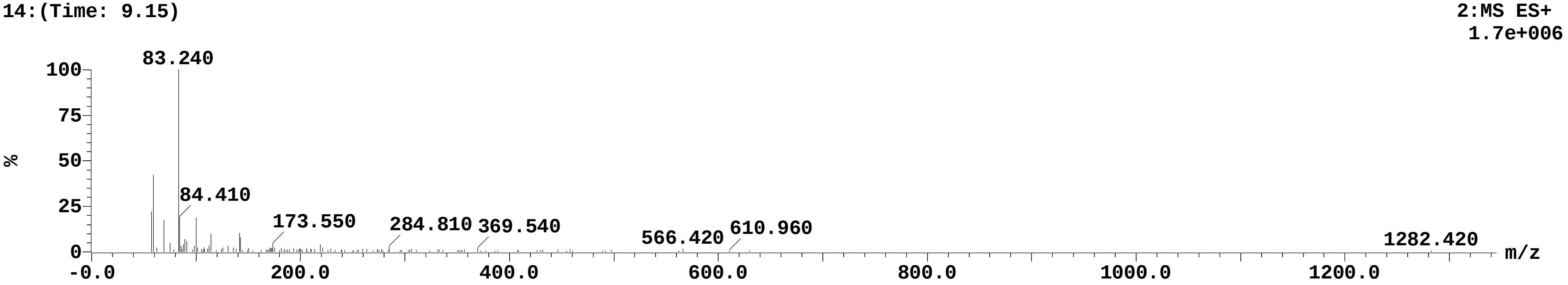

Sample: 642 Vial:1:B,3 ID:
File:LK-1 R Date:03-Aug-2023 Time:12:27:58
Description:Default file

Printed: Thu Aug 03 15:04:15 2023

Sample Report (continued):

Peak ID	Compound	Time	Mass Found
15		10.52	

15:(Time: 10.52)

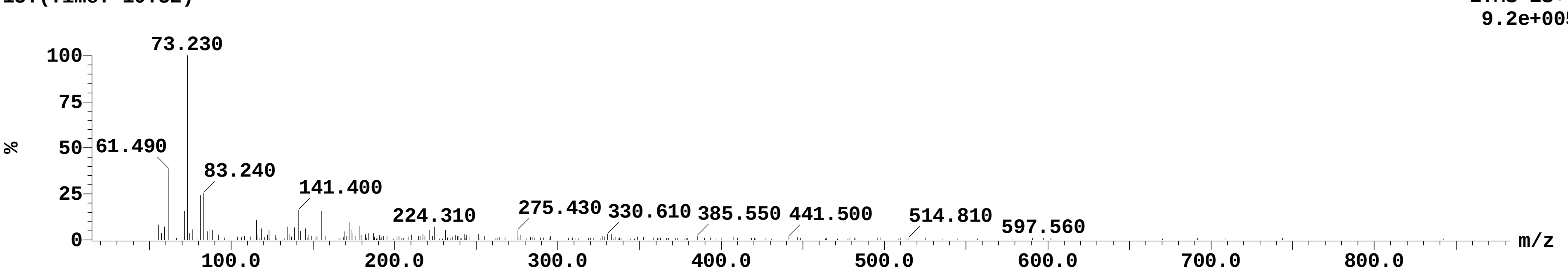

Peak ID	Compound	Time	Mass Found
16		10.53	

16:(Time: 10.53)

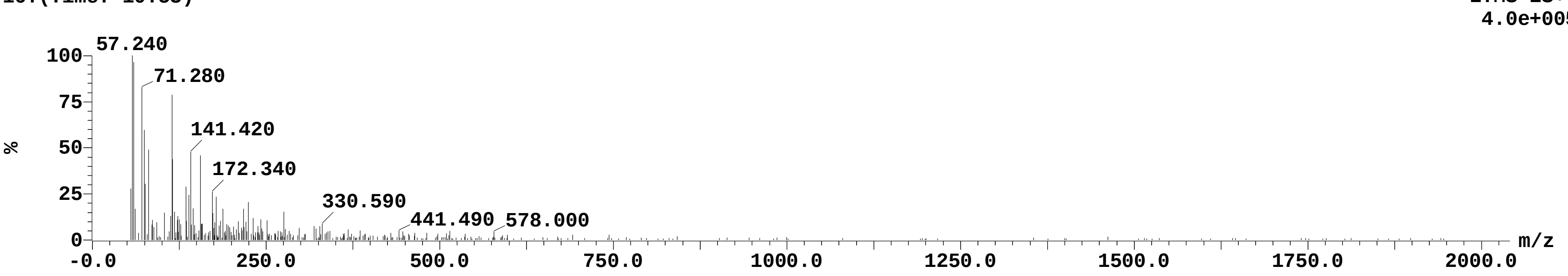

Sample Report (continued):

Peak ID	Compound	Time	Mass Found
17		11.17	

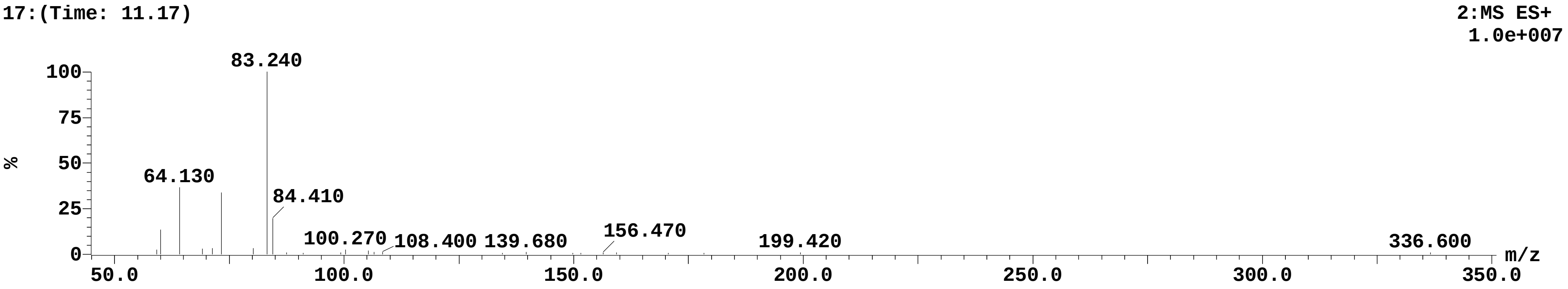

Peak ID	Compound	Time	Mass Found
18		11.27	

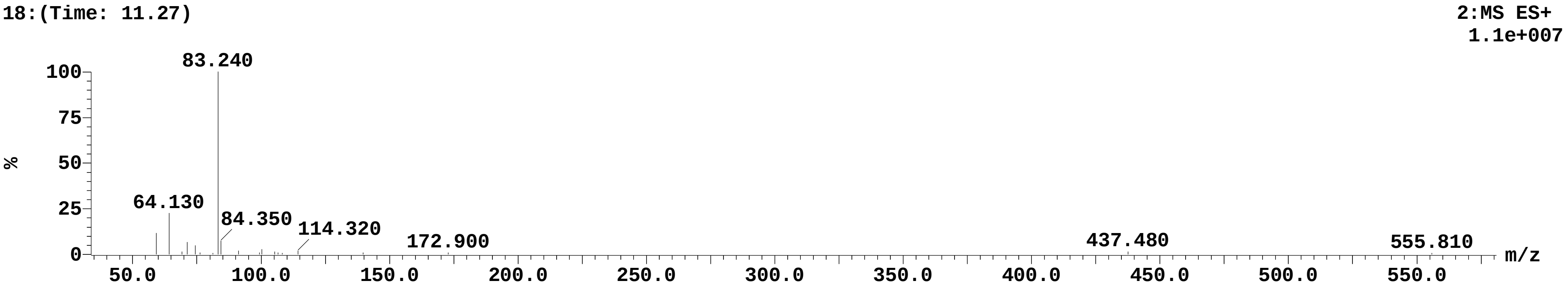

Sample Report (continued):

Peak ID	Compound	Time	Mass Found
19		12.07	

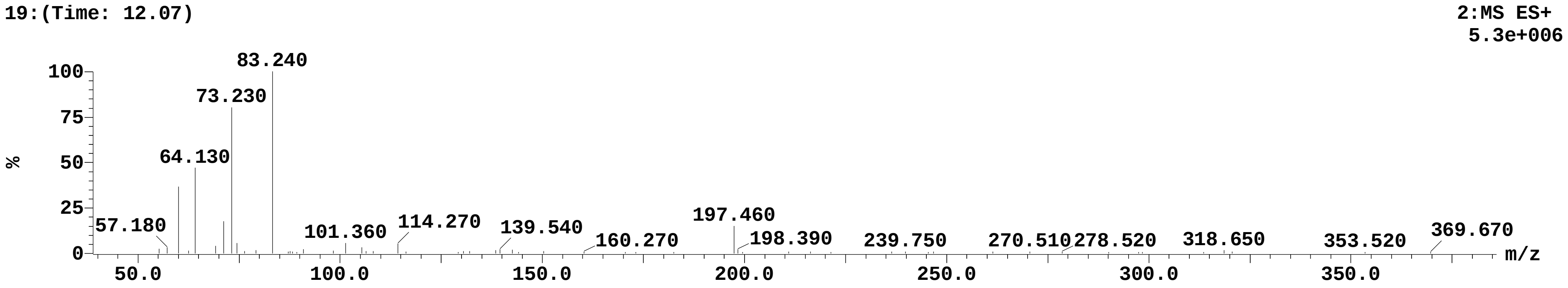

Peak ID	Compound	Time	Mass Found
20		12.07	

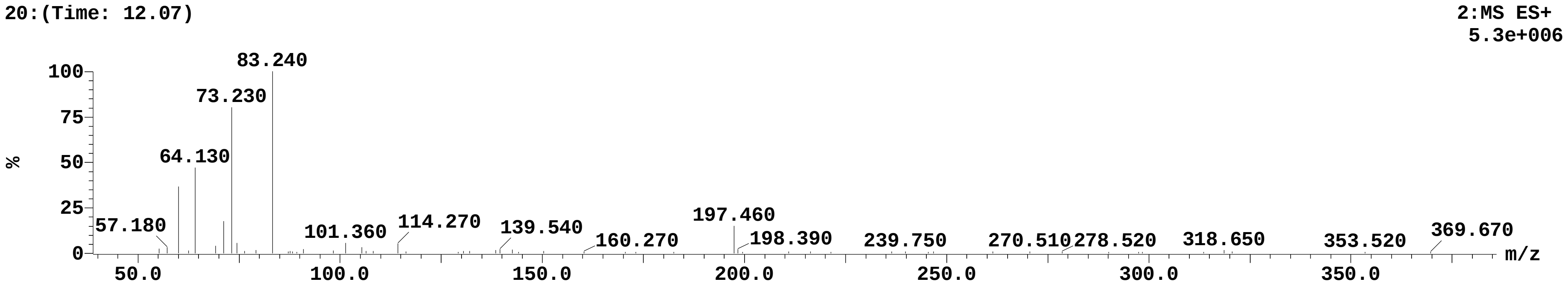

Sample: 642 Vial:1:B,3 ID:
File:LK-1 R Date:03-Aug-2023 Time:12:27:58
Description:Default file

Printed: Thu Aug 03 15:04:15 2023

Sample Report (continued):

Peak ID	Compound	Time	Mass Found
21		13.05	

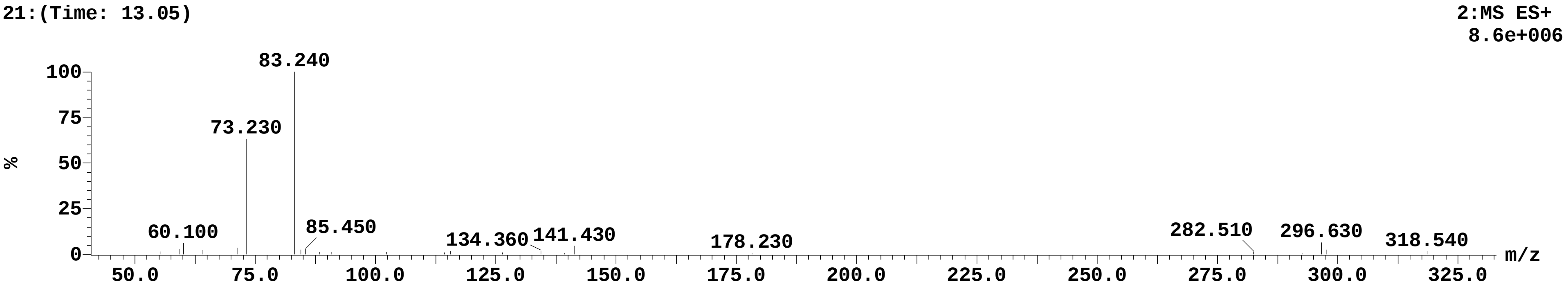

Peak ID	Compound	Time	Mass Found
22		14.17	

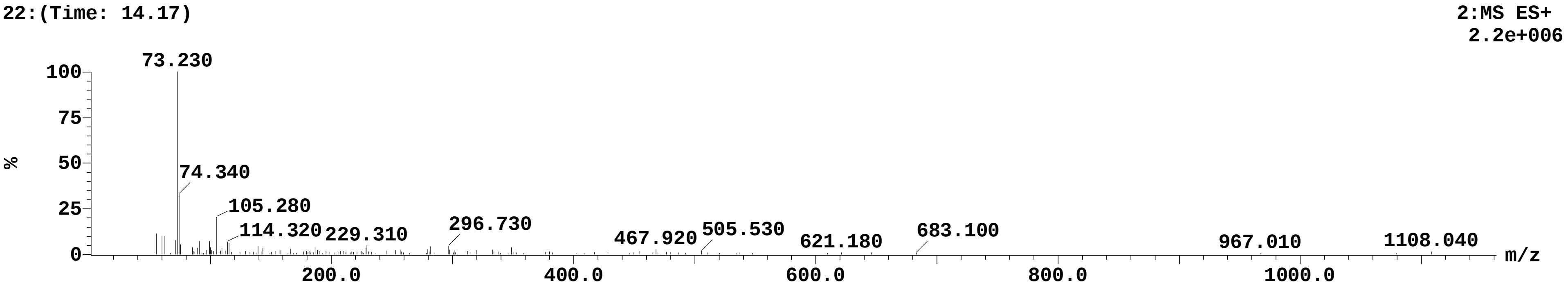

Sample: 642 Vial:1:B,3 ID:
File:LK-1 R Date:03-Aug-2023 Time:12:27:58
Description:Default file

Printed: Thu Aug 03 15:04:15 2023

Sample Report (continued):

Peak ID	Compound	Time	Mass Found
23		14.73	

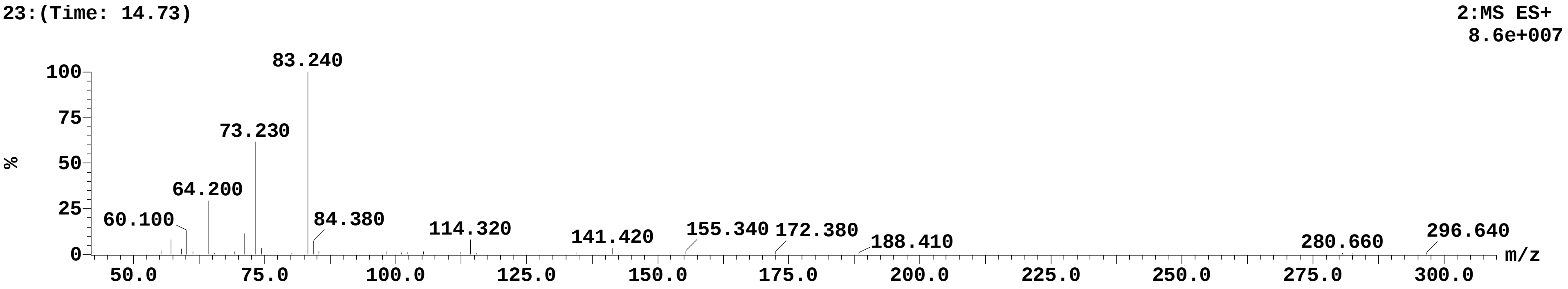

Peak ID	Compound	Time	Mass Found
24		16.48	

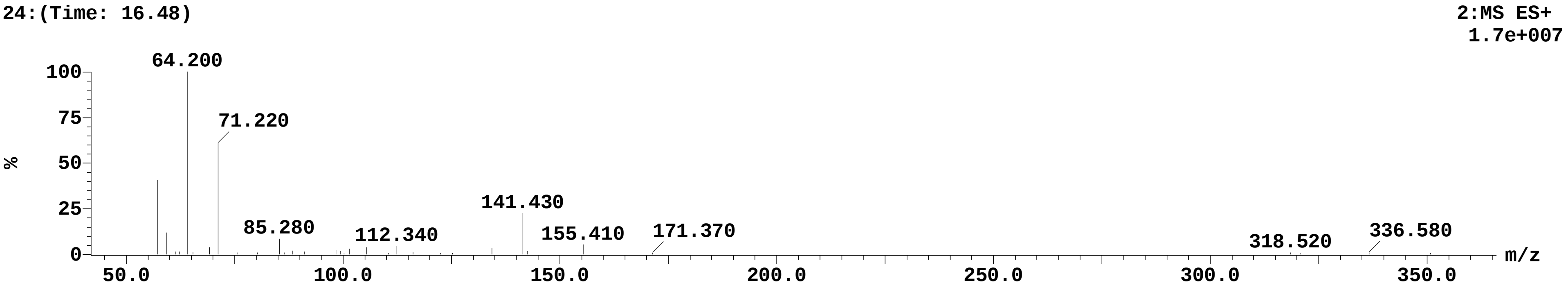

Sample Report (continued):

Peak ID	Compound	Time	Mass Found
25		16.88	

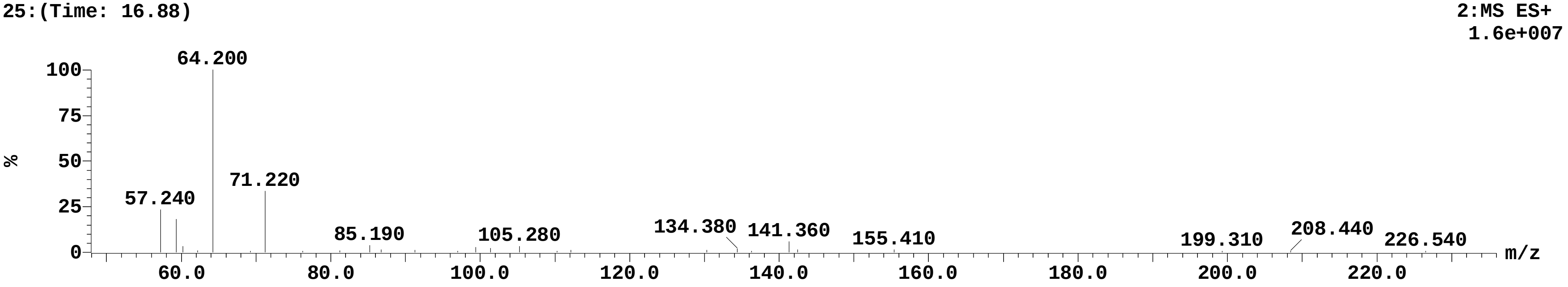

Peak ID	Compound	Time	Mass Found
26		17.28	

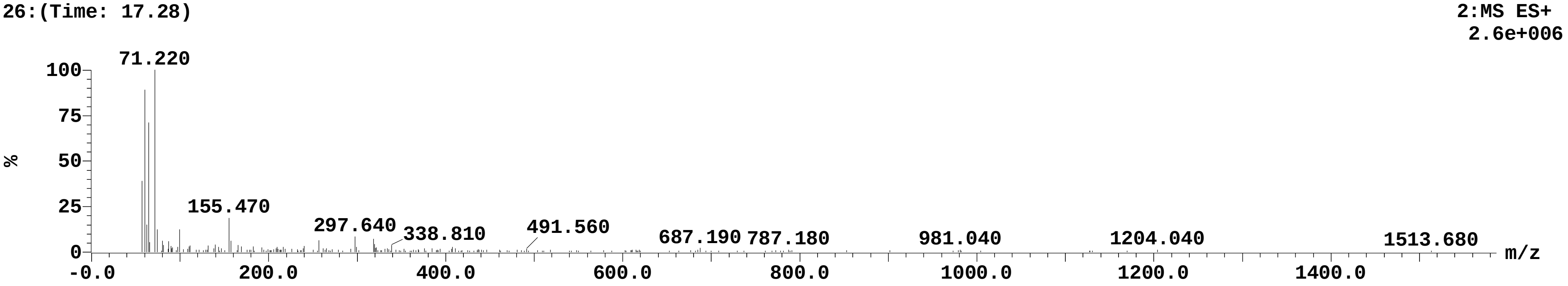

Sample: 642 | Vial:1:B,3 | ID:
File:LK-1 R | Date:03-Aug-2023 | Time:12:27:58
Description:Default file

Printed: Thu Aug 03 15:04:15 2023

Sample Report (continued):

Peak ID	Compound	Time	Mass Found
27		21.70	

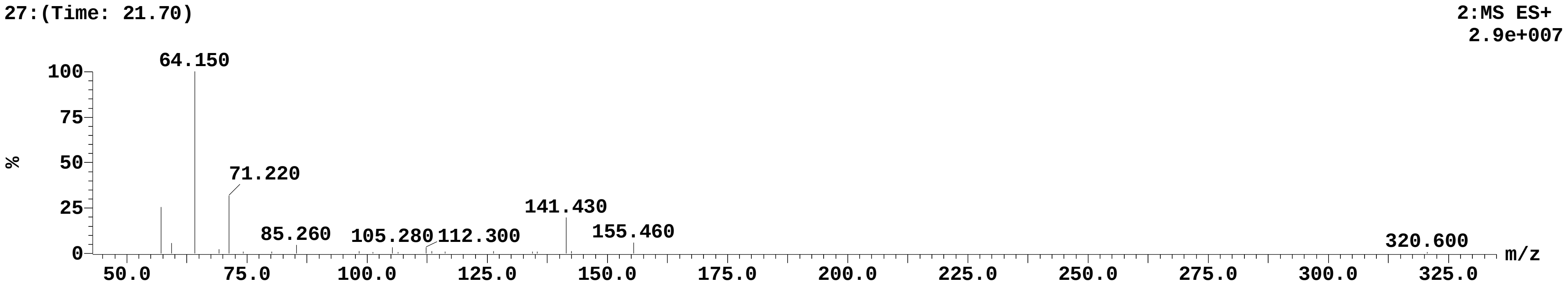

Openlynx Report -
Sample: 643 Vial:1:B,4 ID:
File:LK-2 Date:03-Aug-2023 Time:12:54:05
Description:Default file

Printed: Thu Aug 03 15:05:03 2023

Sample Report:

Sample 643 Vial 1:B,4 ID File LK-2 Date 03-Aug-2023 Time 12:54:05 Description Default file

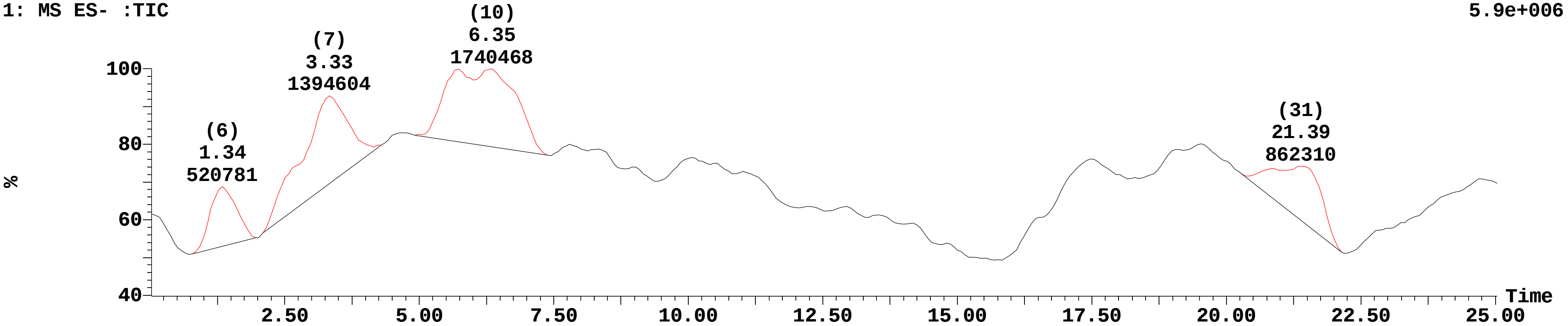

Peak Number	Compound	Time	AreaAbs	Area %Total	Width	Height	Mass Found
6		1.34	5e+005	11.53	1	9e+005	
7		3.33	1e+006	30.87	2	1e+006	
10		6.35	2e+006	38.52	2	1e+006	
31		21.39	9e+005	19.09	2	8e+005	

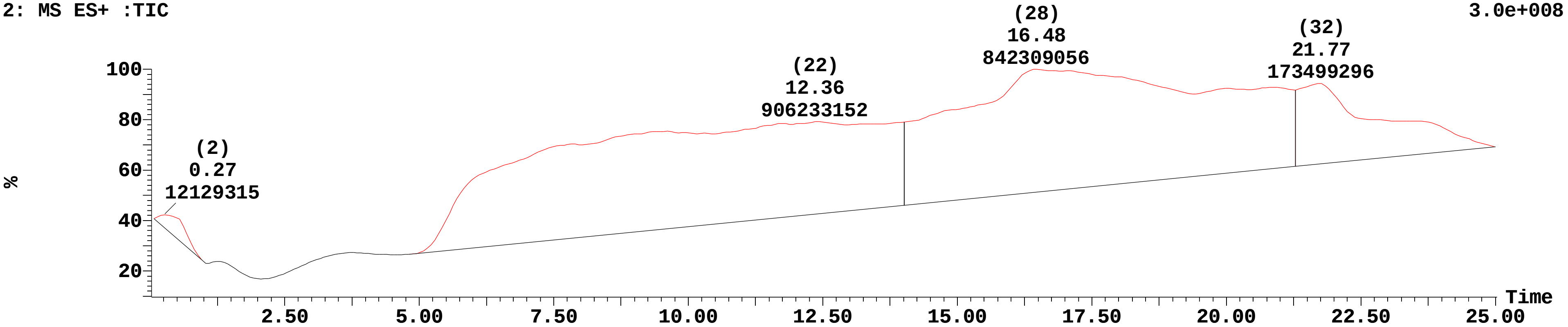

Peak Number	Compound	Time	AreaAbs	Area %Total	Width	Height	Mass Found
2		0.27	1e+007	0.63	1	2e+007	
22		12.36	9e+008	46.85	9	1e+008	

Sample: 643 Vial:1:B,4 ID:
File:LK-2 Date:03-Aug-2023 Time:12:54:05
Description:Default file

Printed: Thu Aug 03 15:05:03 2023

Sample Report (continued):

28	16.48	8e+008	43.55	7	1e+008
32	21.77	2e+008	8.97	4	1e+008

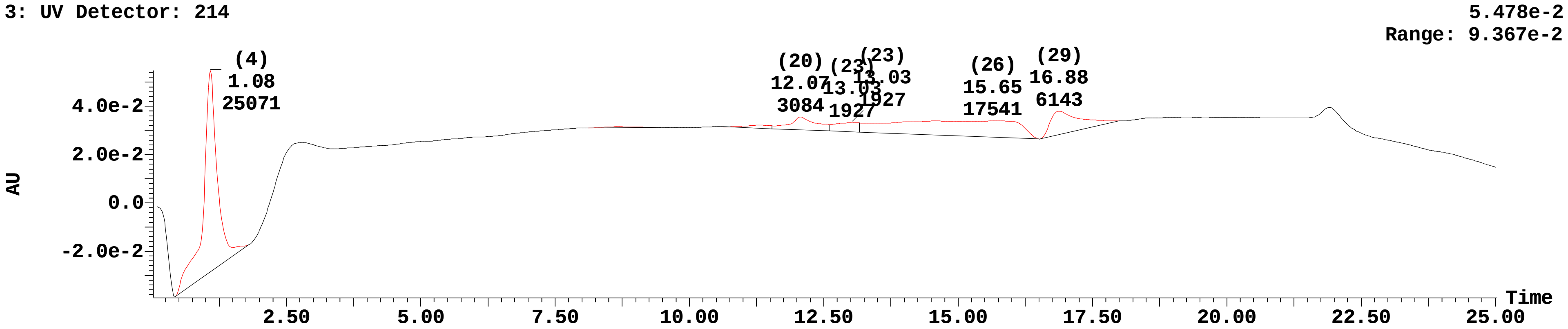

Peak Number	Compound	Time	AreaAbs	Area %Total	Width	Height	Mass Found
4		1.08	3e+004	45.80	1	8e+004	
13		8.67	3e+002	0.59	1	6e+002	
19		11.30	6e+002	1.18	1	1e+003	
20		12.07	3e+003	5.63	1	5e+003	
23		13.03	2e+003	3.52	1	4e+003	
26		15.65	2e+004	32.05	3	7e+003	
29		16.88	6e+003	11.22	1	1e+004	

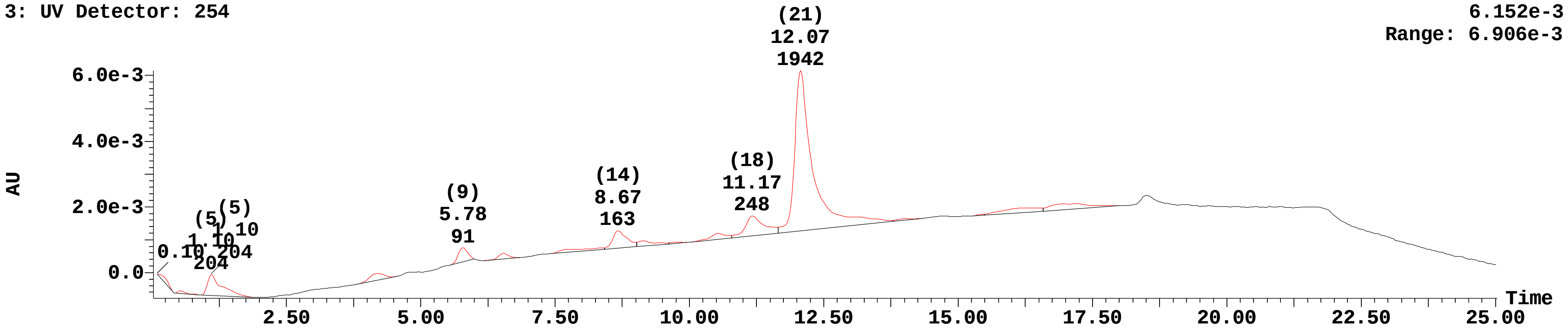

Sample: 643 Vial:1:B,4 ID:
File:LK-2 Date:03-Aug-2023 Time:12:54:05
Description:Default file

Printed: Thu Aug 03 15:05:03 2023

Sample Report (continued):

Peak Number	Compound	Time	AreaAbs	Area %Total	Width	Height	Mass Found
1		0.10	3e+001	0.80	0		
3		0.53	1e+001	0.41	0	8e+001	
5		1.10	2e+002	6.29	1	6e+002	
8		4.22	7e+001	2.02	1	2e+002	
9		5.78	9e+001	2.78	0	4e+002	
11		6.53	4e+001	1.17	1	2e+002	
12		8.42	6e+001	1.79	1	4e+001	
14		8.67	2e+002	5.03	1	5e+002	
15		9.13	5e+001	1.67	1	2e+002	
16		9.77	9e+000	0.28	0	4e+001	
17		10.53	6e+001	1.83	1	2e+002	
18		11.17	2e+002	7.64	1	6e+002	
21		12.07	2e+003	59.74	2	5e+003	
24		14.35	1e+001	0.42	1		
25		15.48	5e+000	0.16	0	3e+001	
27		16.37	1e+002	3.58	1	1e+002	
30		17.20	1e+002	4.39	1	2e+002	

Peak ID	Compound	Time	Mass Found
1		0.10	

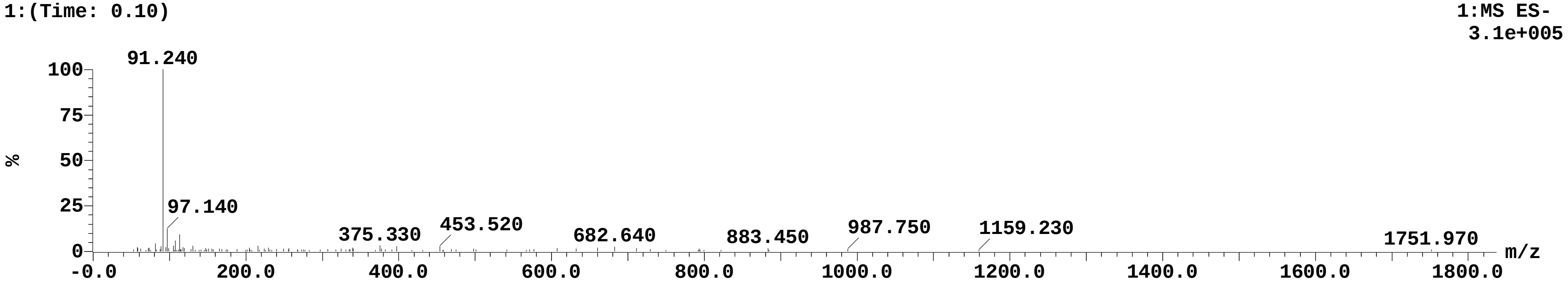

Sample: 643 Vial:1:B,4 ID:

File:LK-2 Date:03-Aug-2023 Time:12:54:05

Description:Default file

Printed: Thu Aug 03 15:05:03 2023

Sample Report (continued):

Peak ID	Compound	Time	Mass Found
3		0.53	

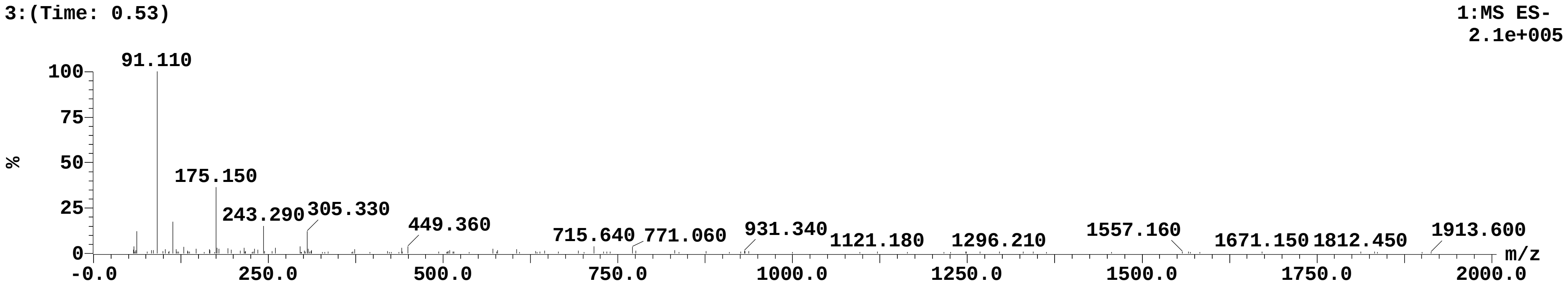

Peak ID	Compound	Time	Mass Found
4		1.08	

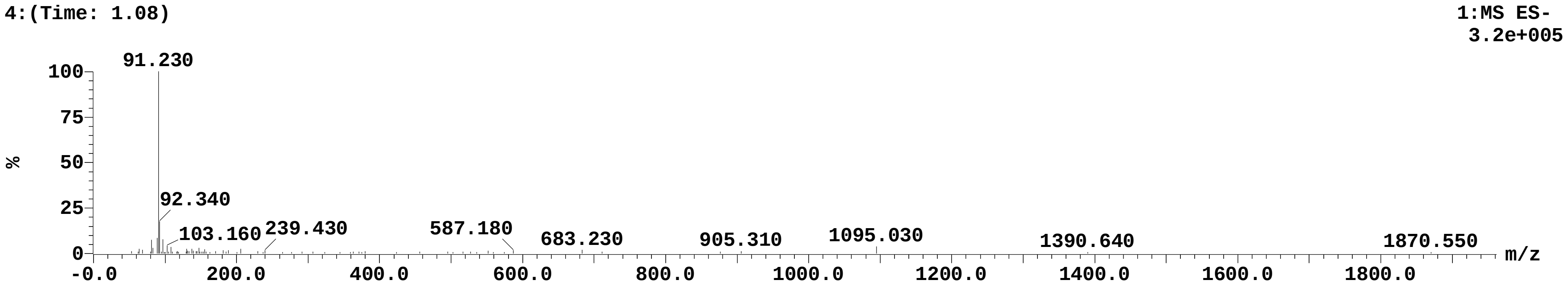

Sample: 643 Vial:1:B,4 ID:
File:LK-2 Date:03-Aug-2023 Time:12:54:05
Description:Default file

Printed: Thu Aug 03 15:05:03 2023

Sample Report (continued):

Peak ID	Compound	Time	Mass Found
5		1.10	

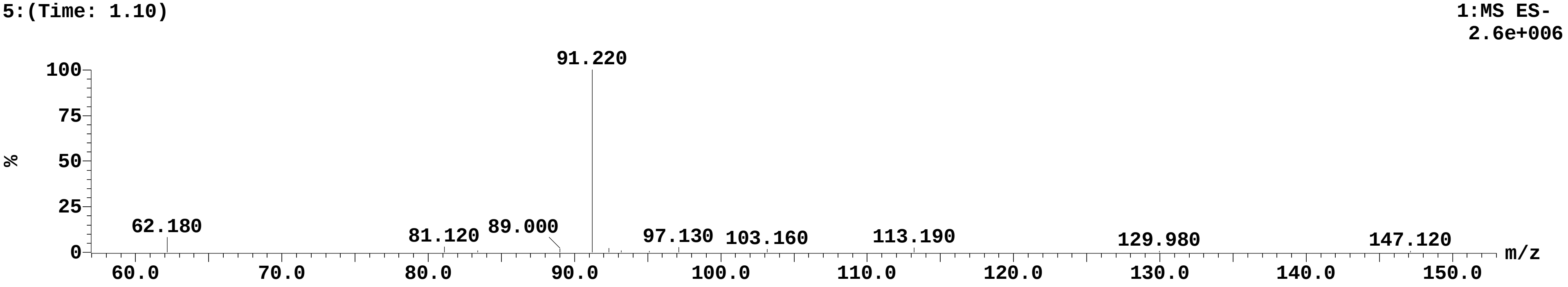

Peak ID	Compound	Time	Mass Found
6		1.34	

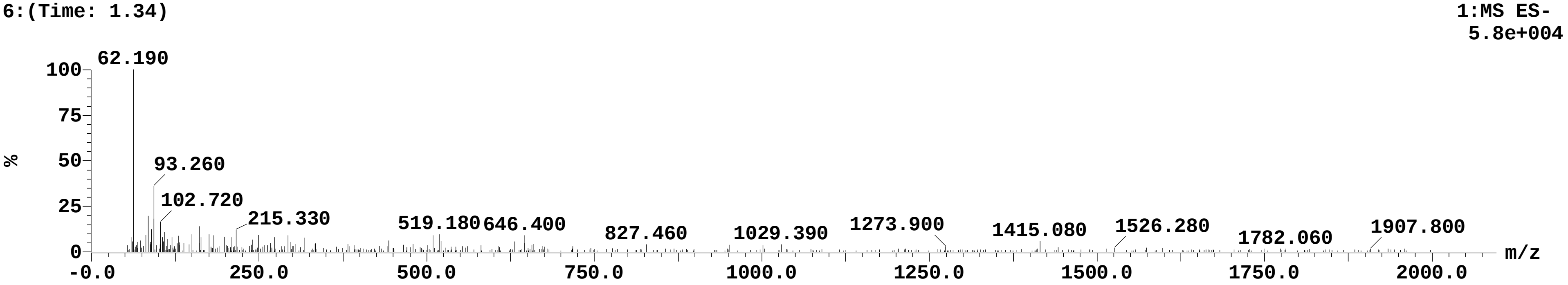

Sample Report (continued):

Peak ID	Compound	Time	Mass Found
7		3.33	

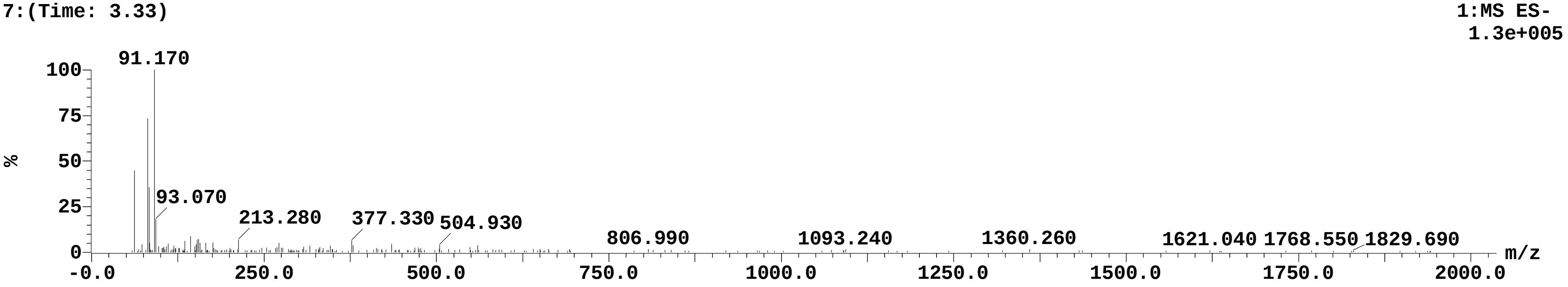

Peak ID	Compound	Time	Mass Found
8		4.22	

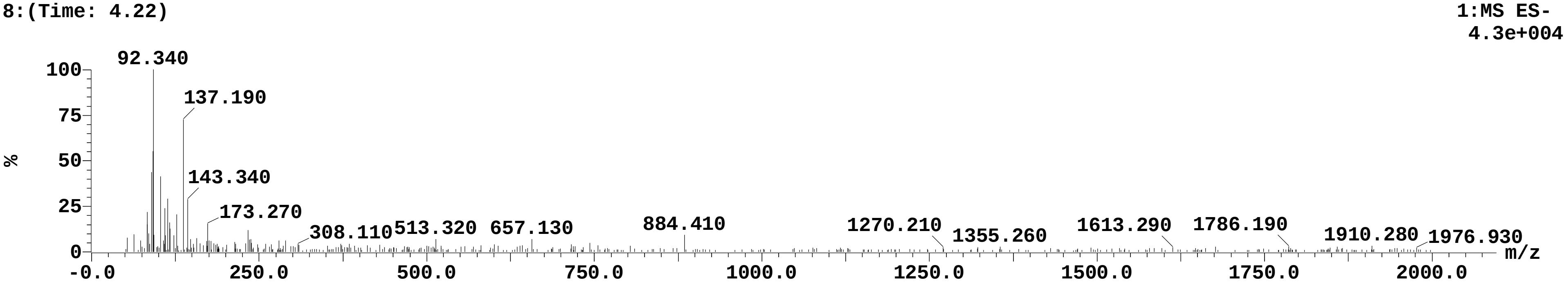

Sample: 643 Vial:1:B,4 ID:
File:LK-2 Date:03-Aug-2023 Time:12:54:05
Description:Default file

Printed: Thu Aug 03 15:05:03 2023

Sample Report (continued):

Peak ID	Compound	Time	Mass Found
9		5.78	

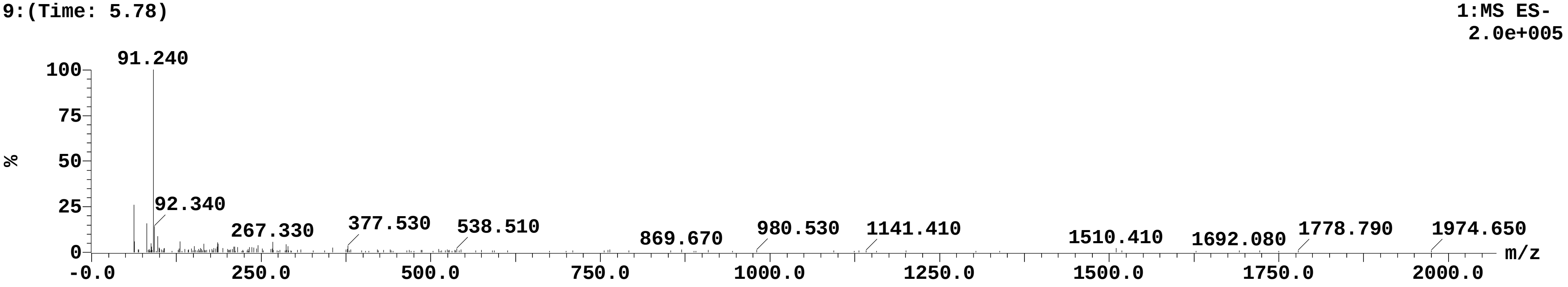

Peak ID	Compound	Time	Mass Found
10		6.35	

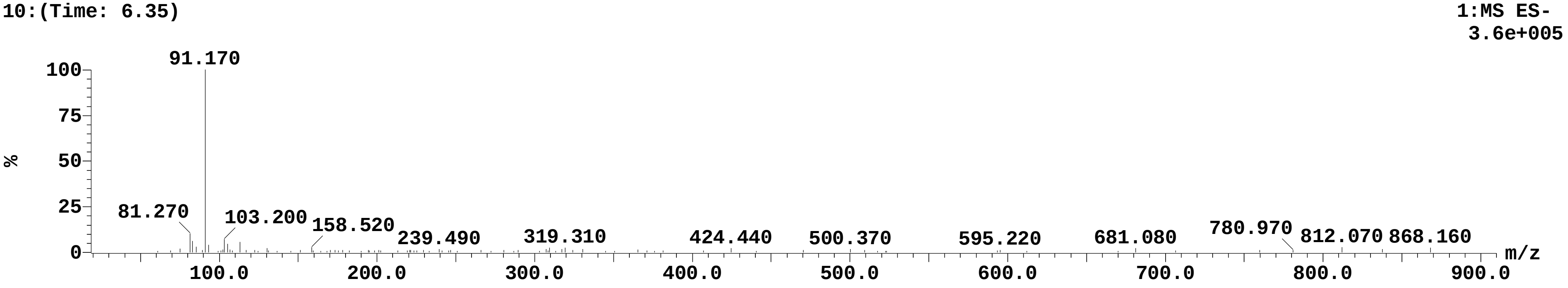

Sample: 643 Vial:1:B,4 ID:
File:LK-2 Date:03-Aug-2023 Time:12:54:05
Description:Default file

Printed: Thu Aug 03 15:05:03 2023

Sample Report (continued):

Peak ID	Compound	Time	Mass Found
11		6.53	

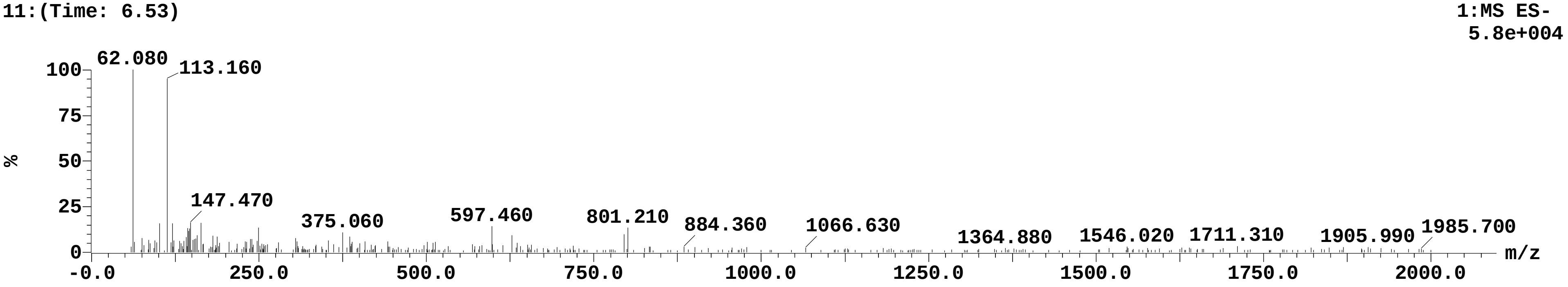

Peak ID	Compound	Time	Mass Found
12		8.42	

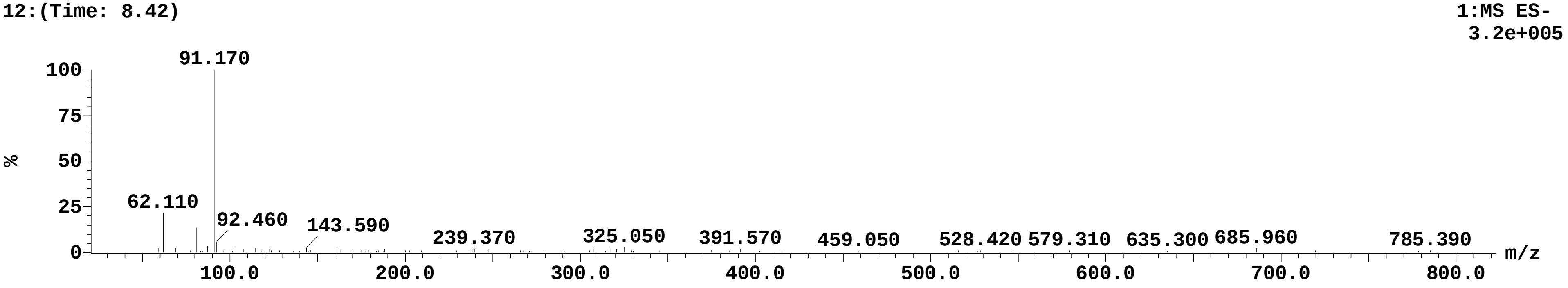

Sample Report (continued):

Peak ID	Compound	Time	Mass Found
13		8.67	

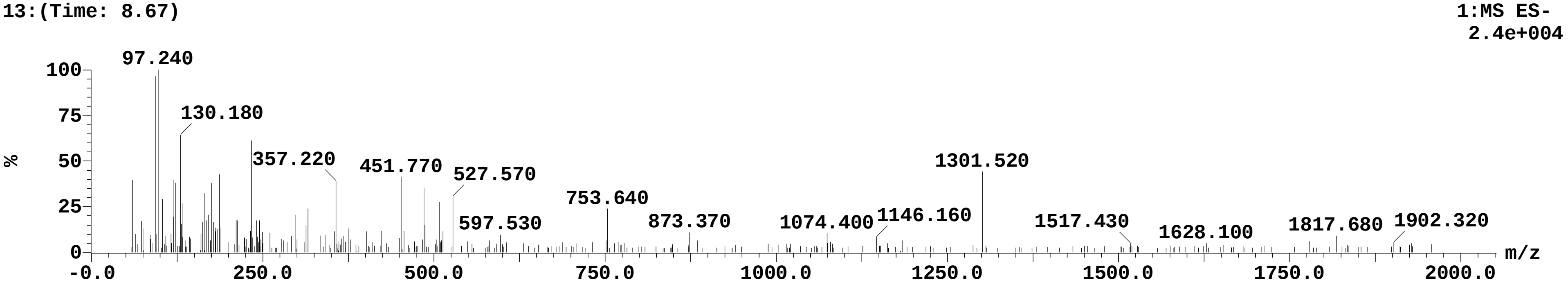

Peak ID	Compound	Time	Mass Found
14		8.67	

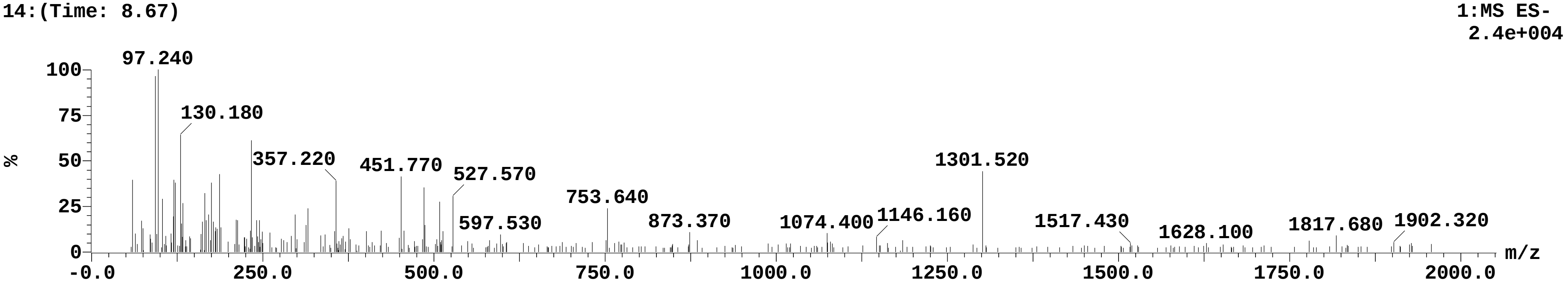

Sample: 643 Vial:1:B,4 ID:
File:LK-2 Date:03-Aug-2023 Time:12:54:05
Description:Default file

Printed: Thu Aug 03 15:05:03 2023

Sample Report (continued):

Peak ID	Compound	Time	Mass Found
15		9.13	

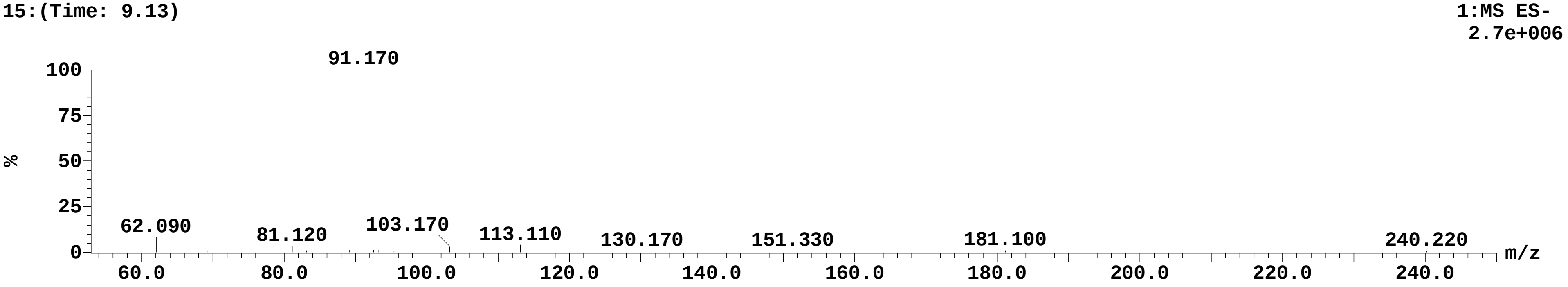

Peak ID	Compound	Time	Mass Found
16		9.77	

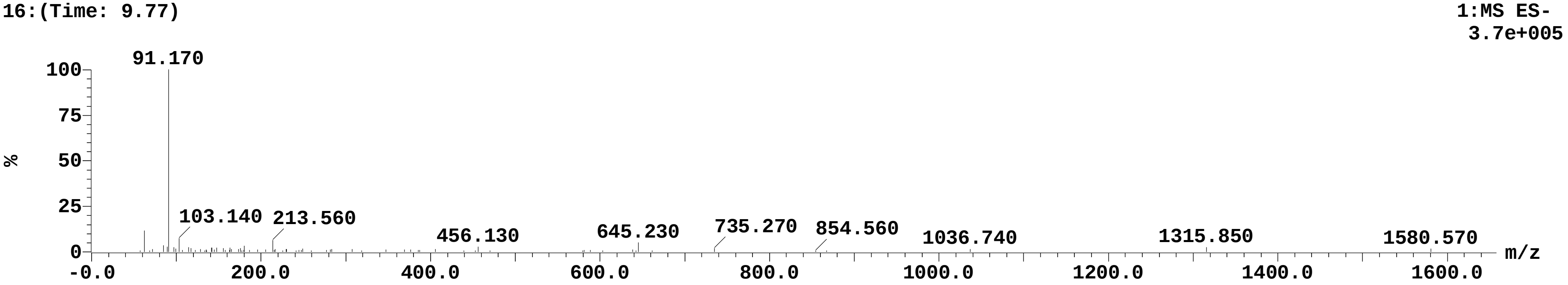

Sample Report (continued):

Peak ID	Compound	Time	Mass Found
17		10.53	

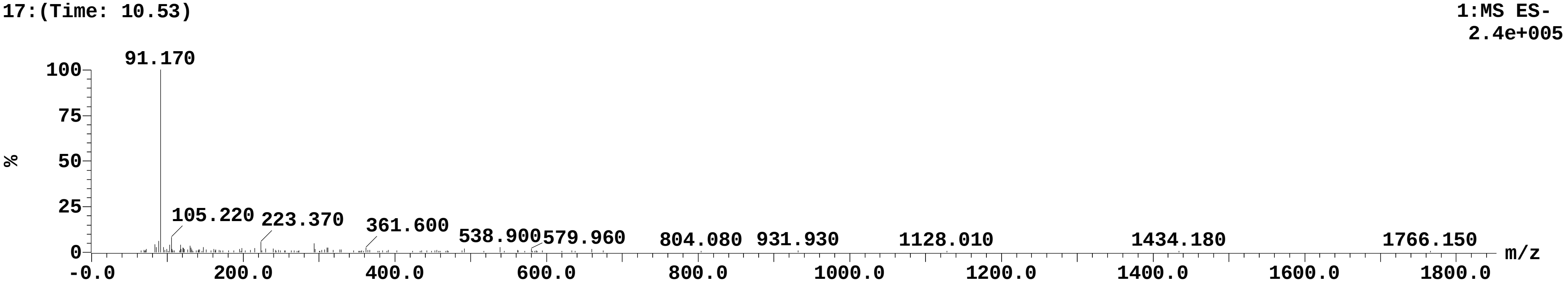

Peak ID	Compound	Time	Mass Found
18		11.17	

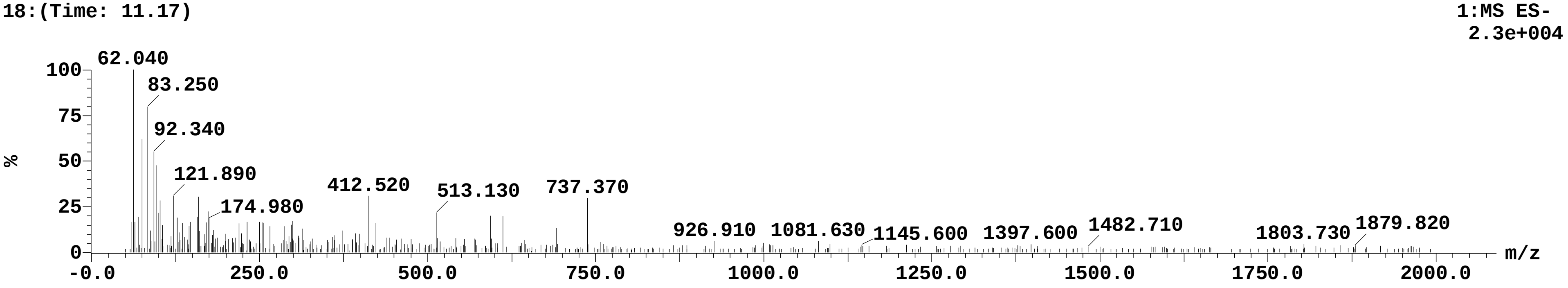

Sample: 643 Vial:1:B,4 ID:
File:LK-2 Date:03-Aug-2023 Time:12:54:05
Description:Default file

Printed: Thu Aug 03 15:05:03 2023

Sample Report (continued):

Peak ID	Compound	Time	Mass Found
19		11.30	

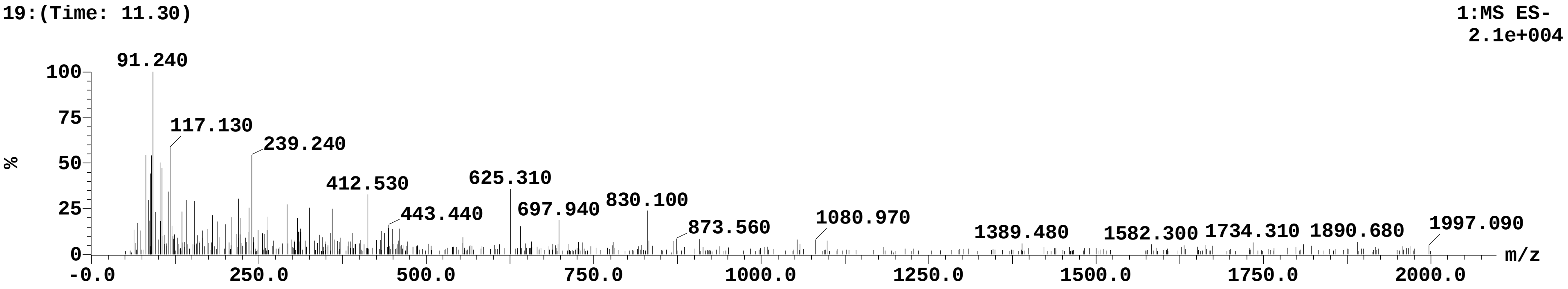

Peak ID	Compound	Time	Mass Found
20		12.07	

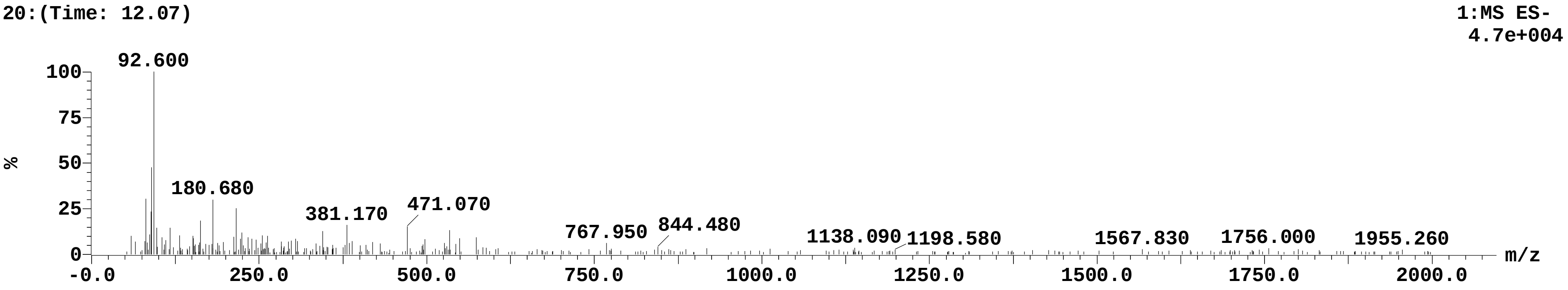

Sample Report (continued):

Peak ID	Compound	Time	Mass Found
21		12.07	

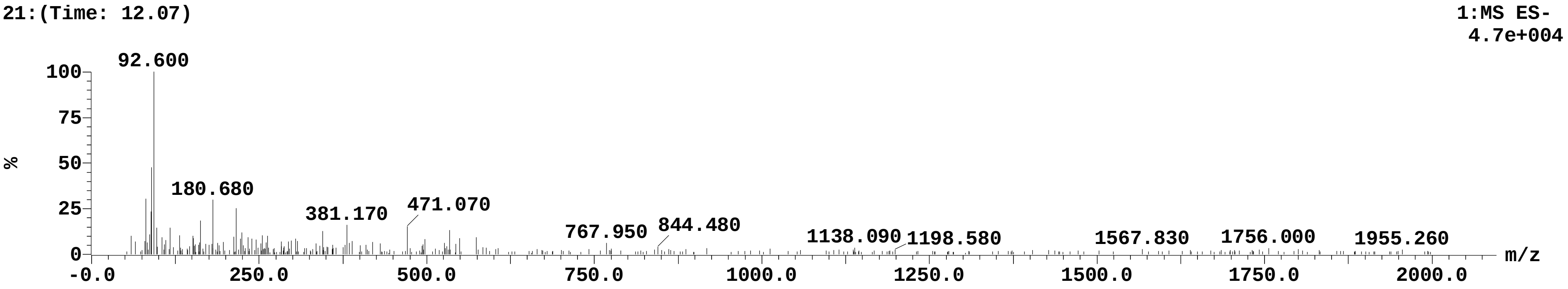

Peak ID	Compound	Time	Mass Found
23		13.03	

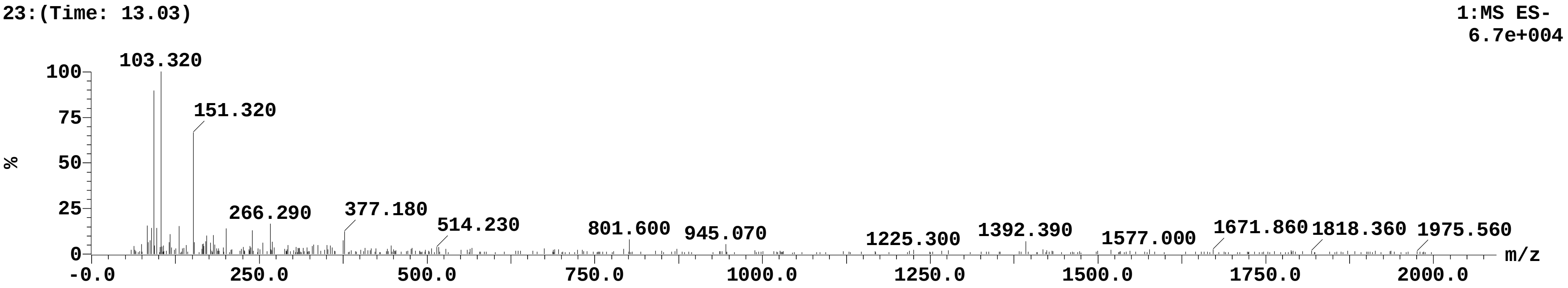

Sample: 643 Vial:1:B,4 ID:
File:LK-2 Date:03-Aug-2023 Time:12:54:05
Description:Default file

Printed: Thu Aug 03 15:05:03 2023

Sample Report (continued):

Peak ID	Compound	Time	Mass Found
24		14.35	

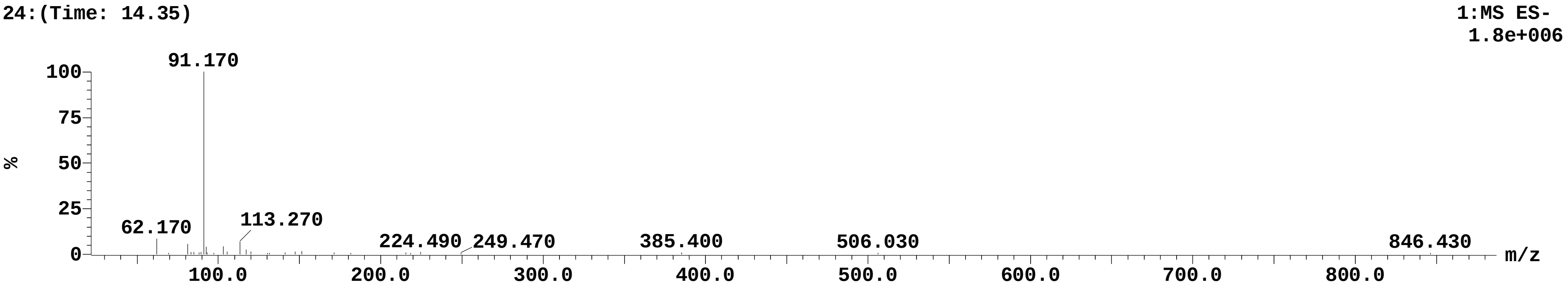

Peak ID	Compound	Time	Mass Found
25		15.48	

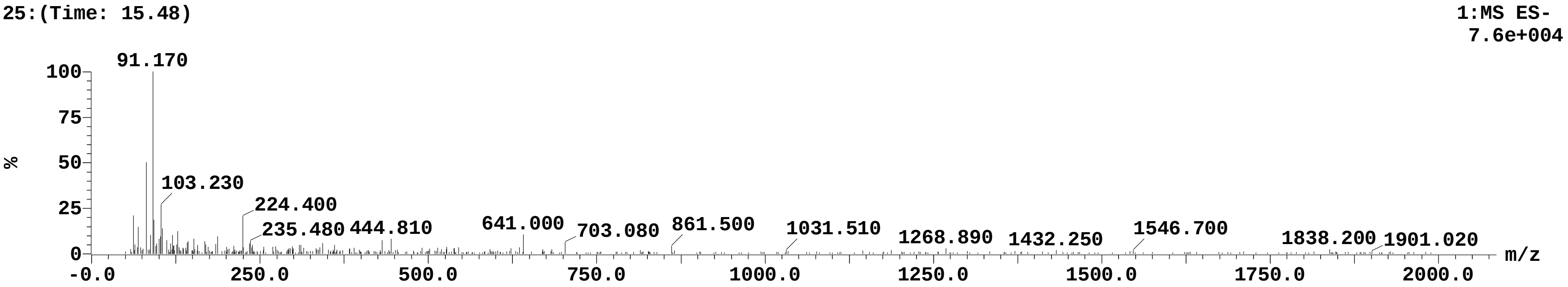

Sample: 643 Vial:1:B,4 ID:
File:LK-2 Date:03-Aug-2023 Time:12:54:05
Description:Default file

Printed: Thu Aug 03 15:05:03 2023

Sample Report (continued):

Peak ID	Compound	Time	Mass Found
26		15.65	

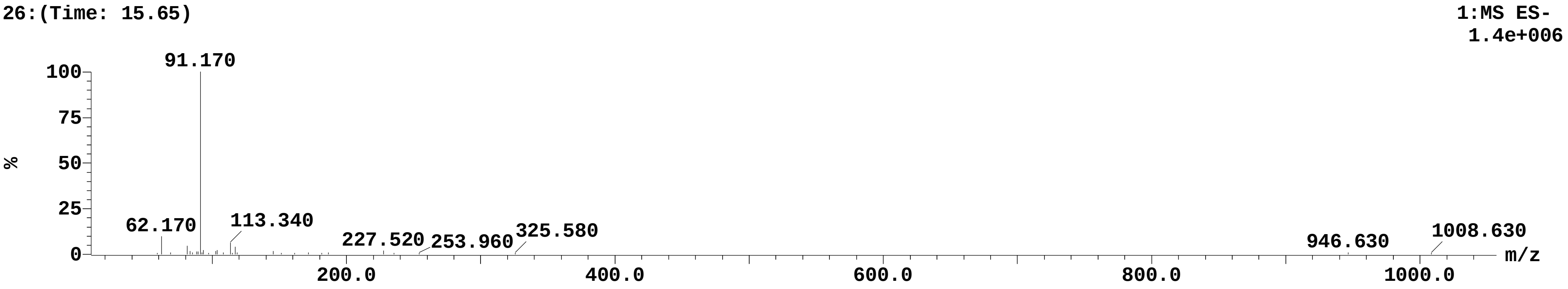

Peak ID	Compound	Time	Mass Found
27		16.37	

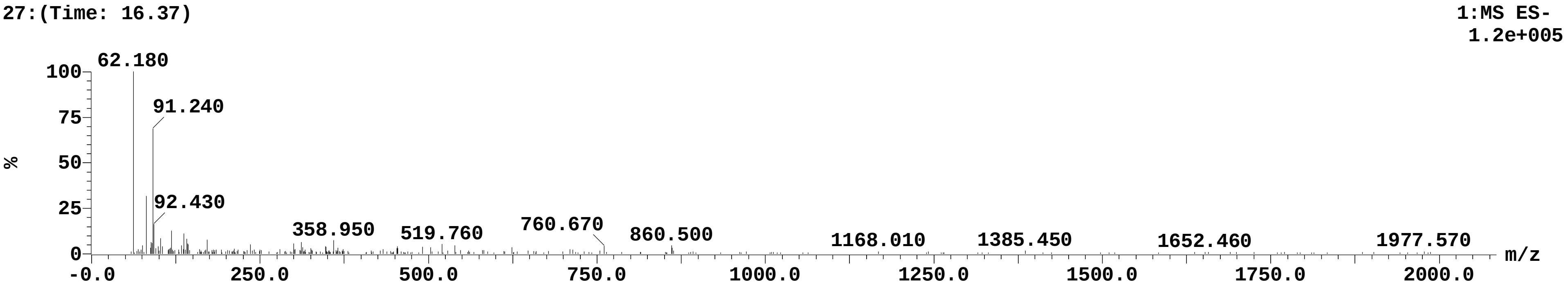

Sample Report (continued):

Peak ID	Compound	Time	Mass Found
29		16.88	

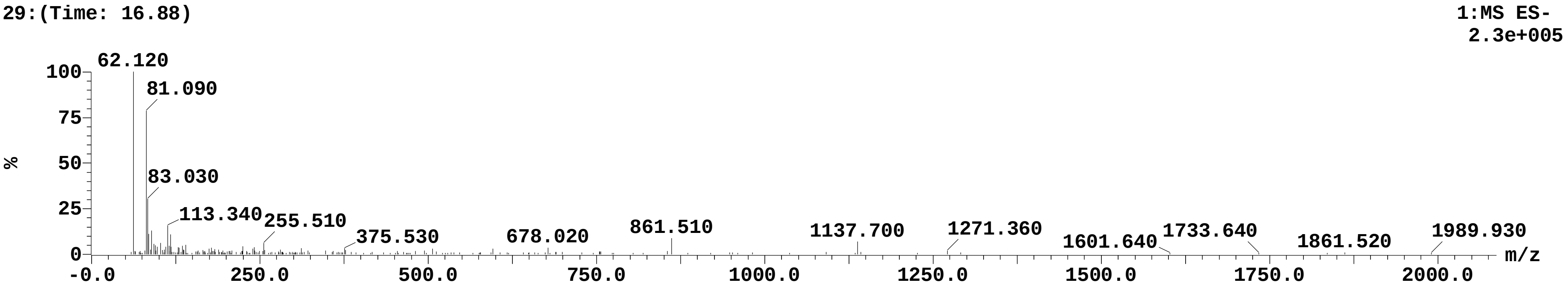

Peak ID	Compound	Time	Mass Found
30		17.20	

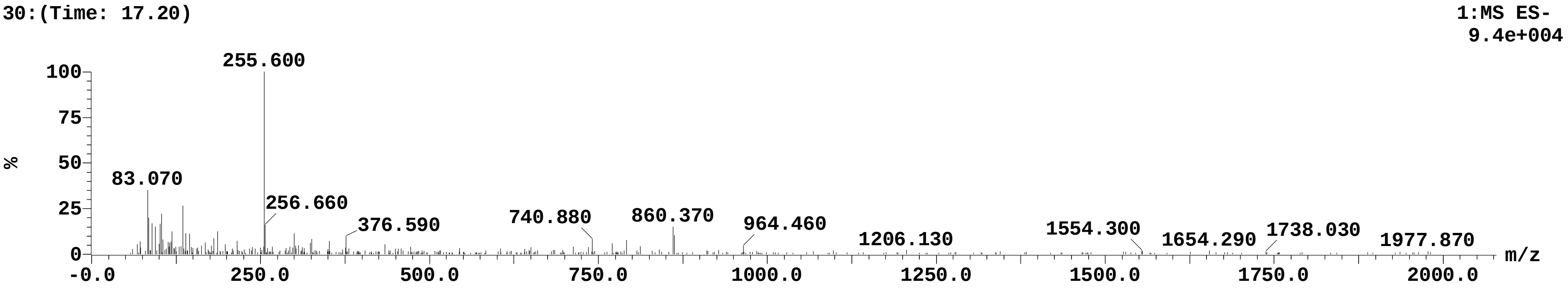

Sample Report (continued):

Peak ID	Compound	Time	Mass Found
31		21.39	

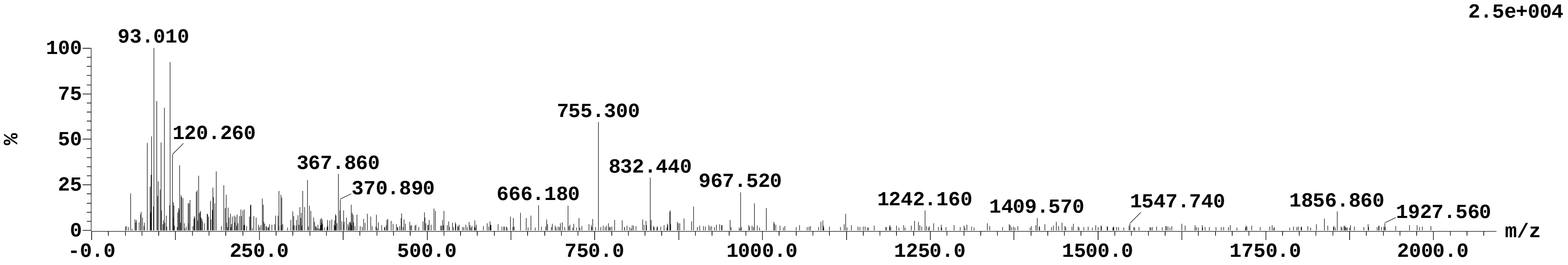

Peak ID	Compound	Time	Mass Found
1		0.10	

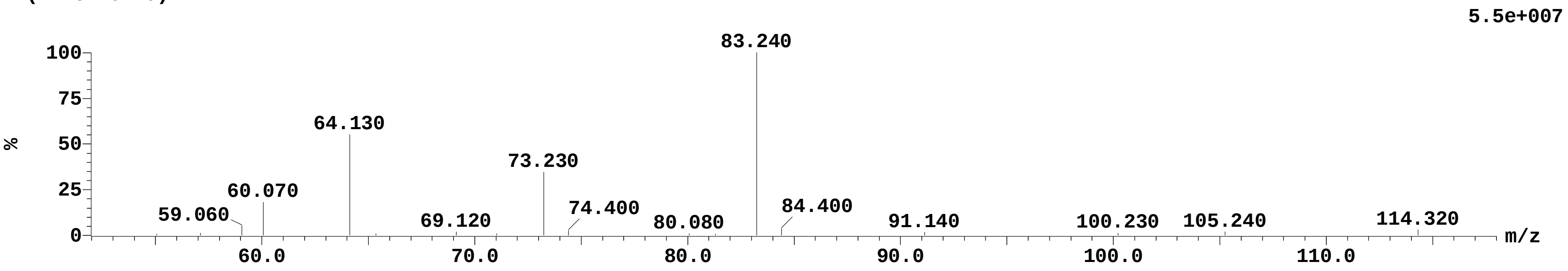

Sample Report (continued):

Peak ID	Compound	Time	Mass Found
2		0.27	

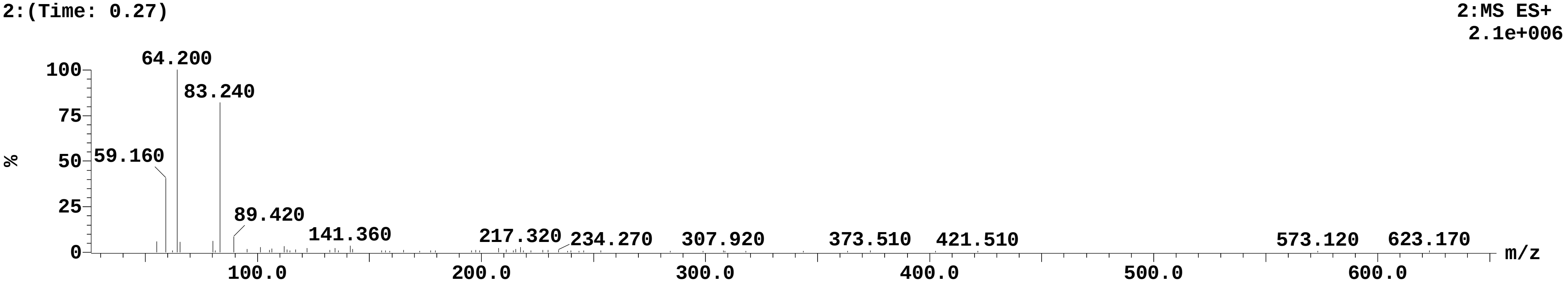

Peak ID	Compound	Time	Mass Found
3		0.53	

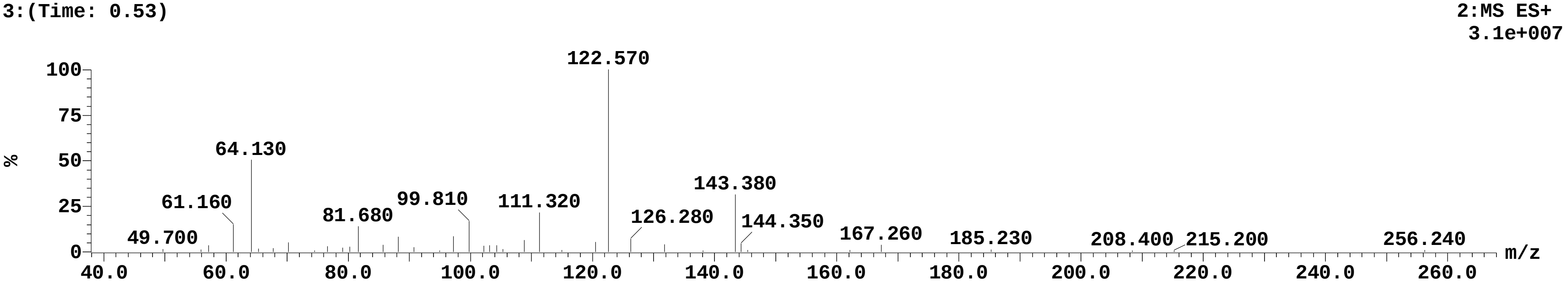

Sample: 643 Vial:1:B,4 ID:
File:LK-2 Date:03-Aug-2023 Time:12:54:05
Description:Default file

Printed: Thu Aug 03 15:05:03 2023

Sample Report (continued):

Peak ID	Compound	Time	Mass Found
4		1.08	

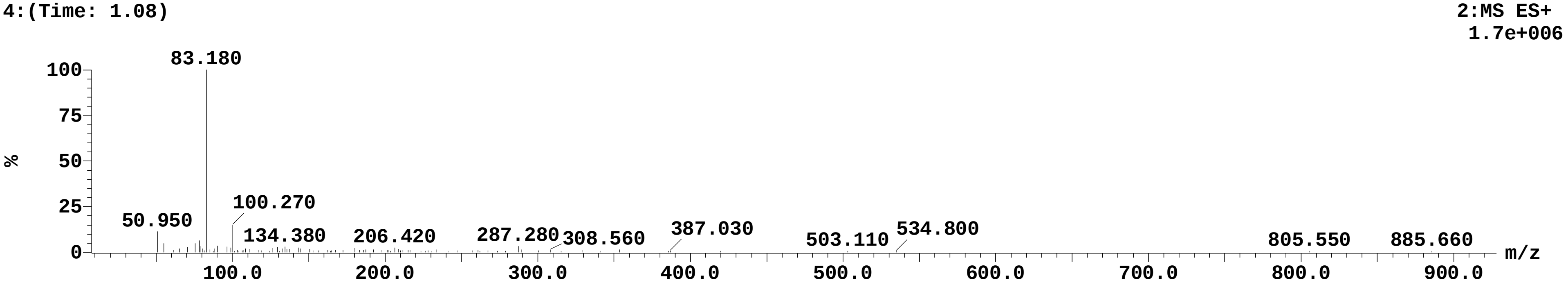

Peak ID	Compound	Time	Mass Found
5		1.10	

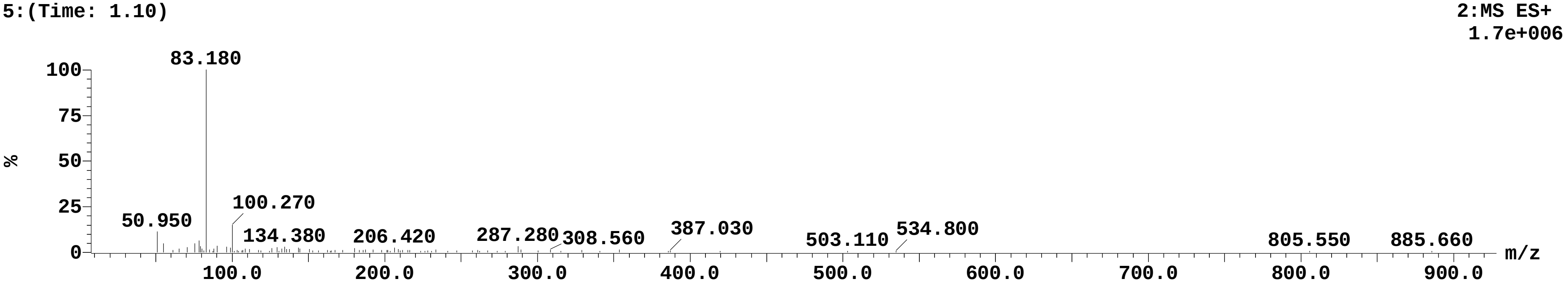

Sample: 643 Vial:1:B,4 ID:
File:LK-2 Date:03-Aug-2023 Time:12:54:05
Description:Default file

Printed: Thu Aug 03 15:05:03 2023

Sample Report (continued):

Peak ID	Compound	Time	Mass Found
8		4.22	

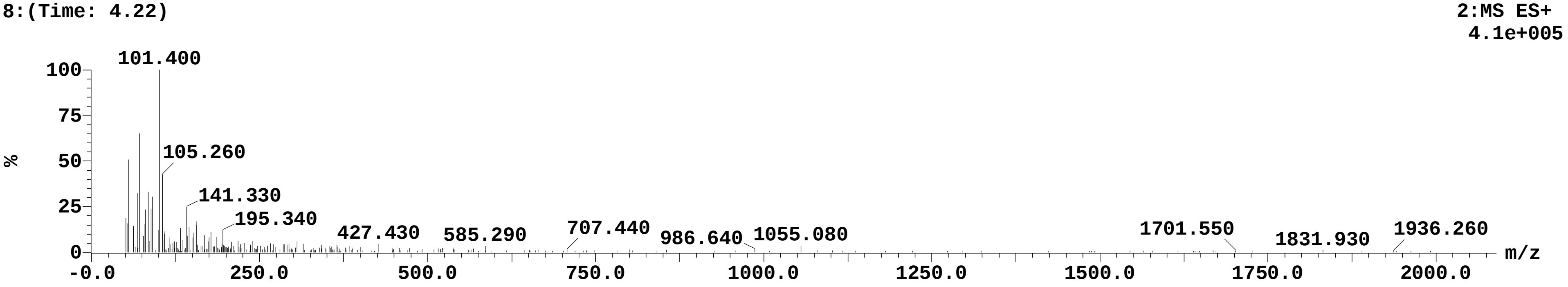

Peak ID	Compound	Time	Mass Found
9		5.78	

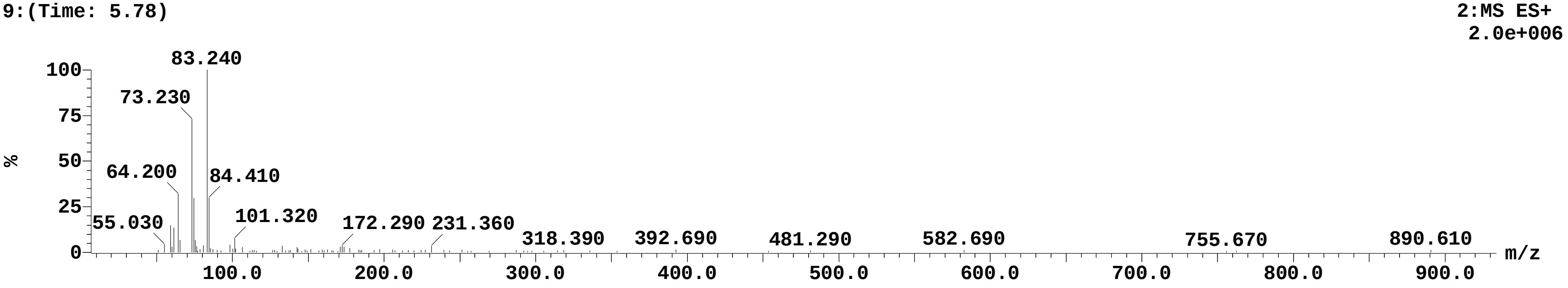

Sample Report (continued):

Peak ID	Compound	Time	Mass Found
11		6.53	

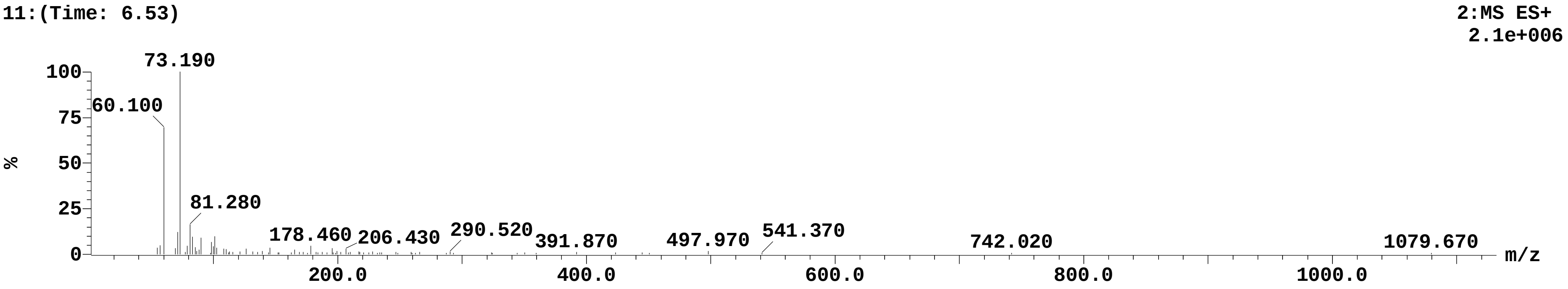

Peak ID	Compound	Time	Mass Found
12		8.42	

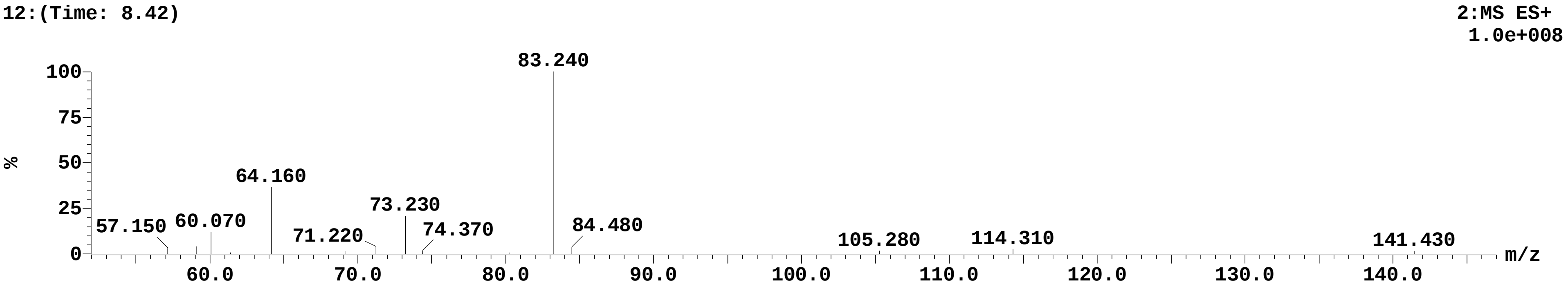

Sample: 643 Vial:1:B,4 ID:
File:LK-2 Date:03-Aug-2023 Time:12:54:05
Description:Default file

Printed: Thu Aug 03 15:05:03 2023

Sample Report (continued):

Peak ID	Compound	Time	Mass Found
13		8.67	

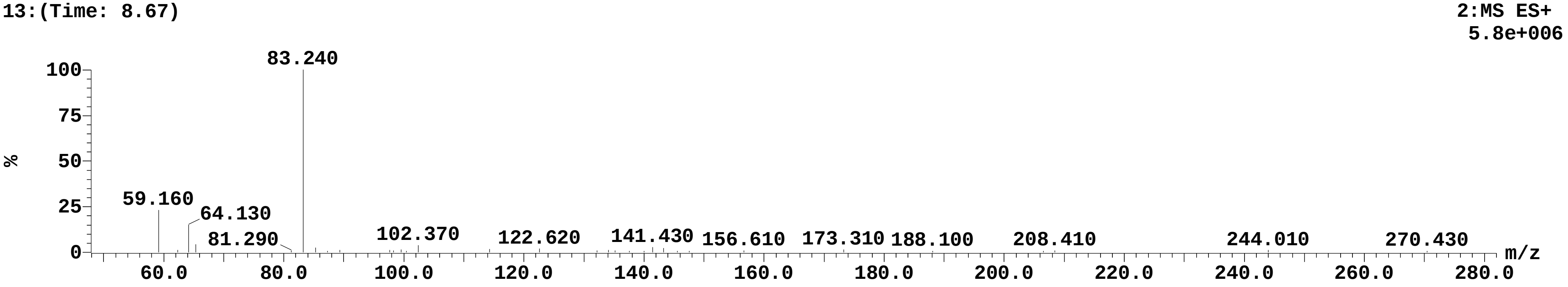

Peak ID	Compound	Time	Mass Found
14		8.67	

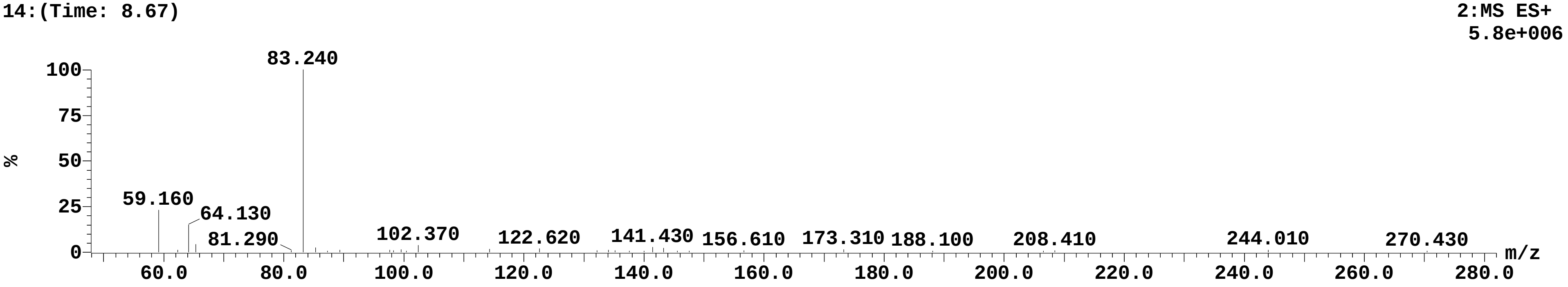

Sample: 643 Vial:1:B,4 ID:
File:LK-2 Date:03-Aug-2023 Time:12:54:05
Description:Default file

Printed: Thu Aug 03 15:05:03 2023

Sample Report (continued):

Peak ID	Compound	Time	Mass Found
15		9.13	

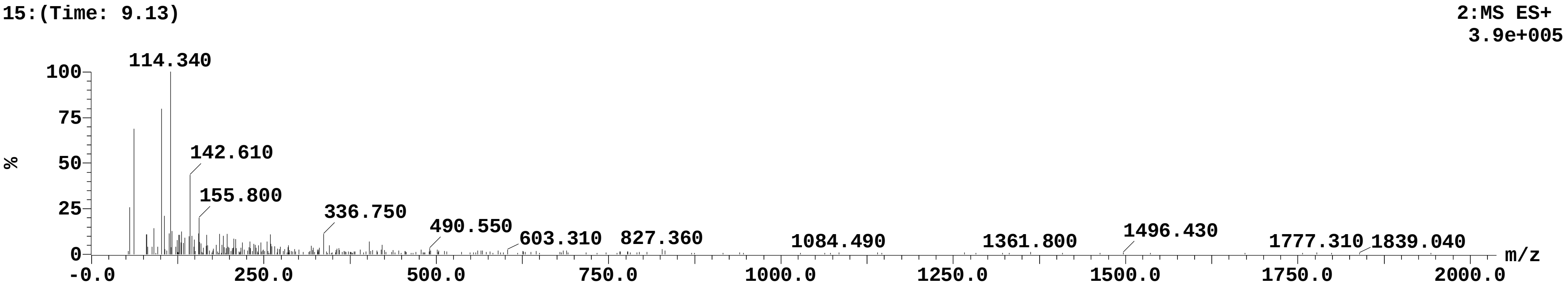

Peak ID	Compound	Time	Mass Found
16		9.77	

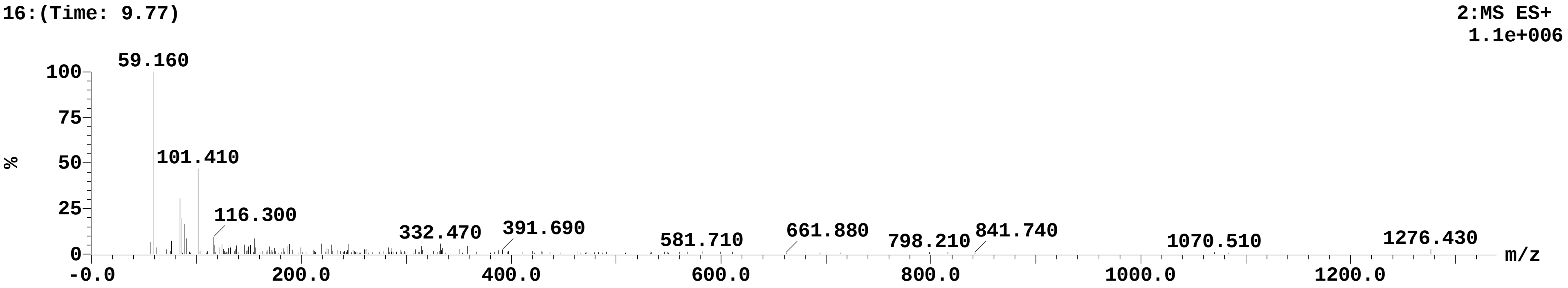

Sample Report (continued):

Peak ID	Compound	Time	Mass Found
17		10.53	

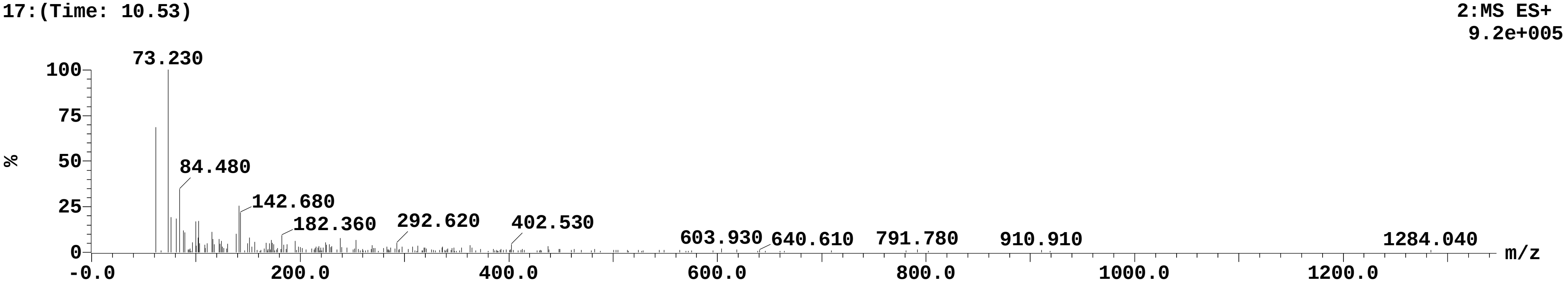

Peak ID	Compound	Time	Mass Found
18		11.17	

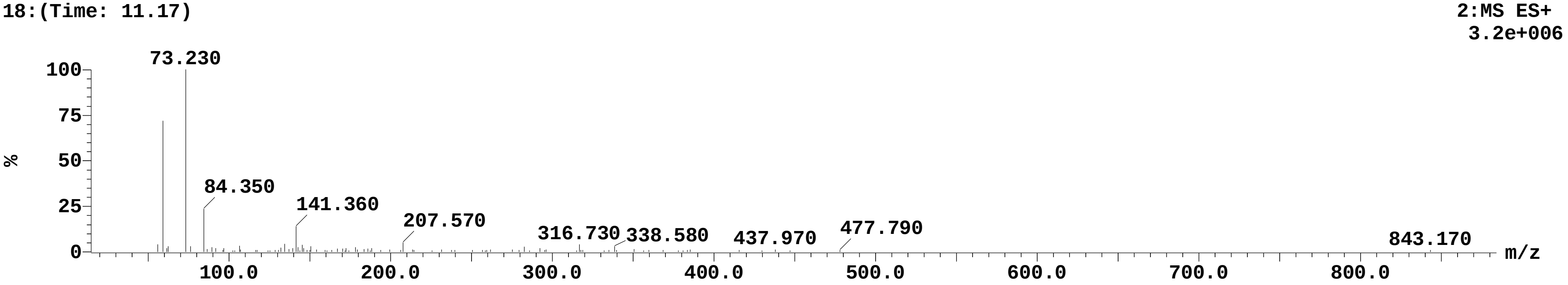

Sample: 643 Vial:1:B,4 ID:
File:LK-2 Date:03-Aug-2023 Time:12:54:05
Description:Default file

Printed: Thu Aug 03 15:05:03 2023

Sample Report (continued):

Peak ID	Compound	Time	Mass Found
19		11.30	

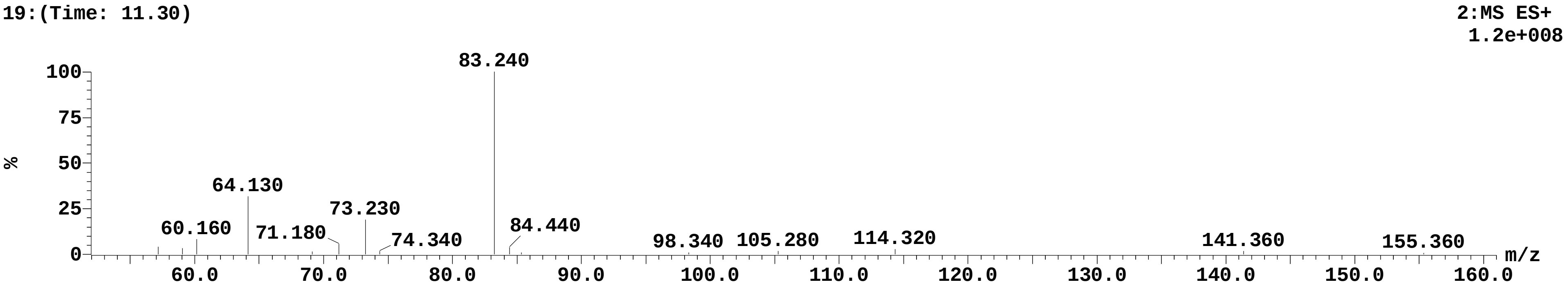

Peak ID	Compound	Time	Mass Found
20		12.07	

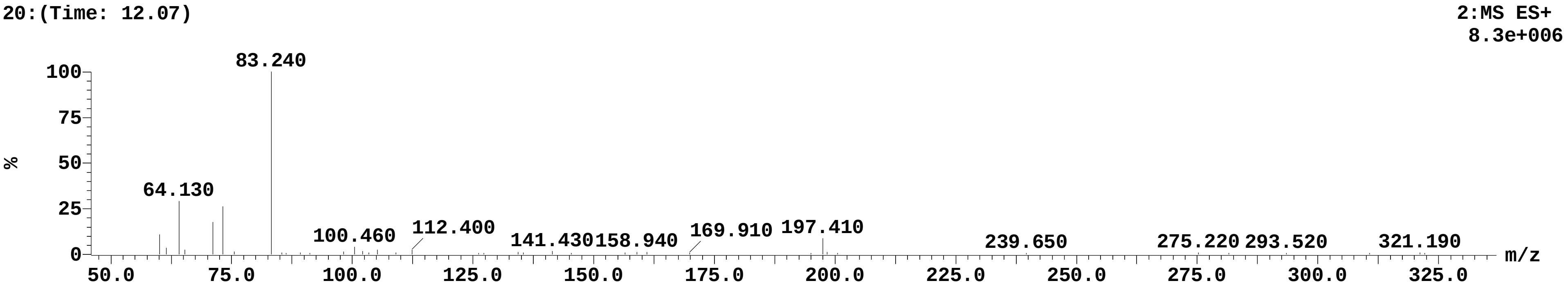

Sample Report (continued):

Peak ID	Compound	Time	Mass Found
21		12.07	

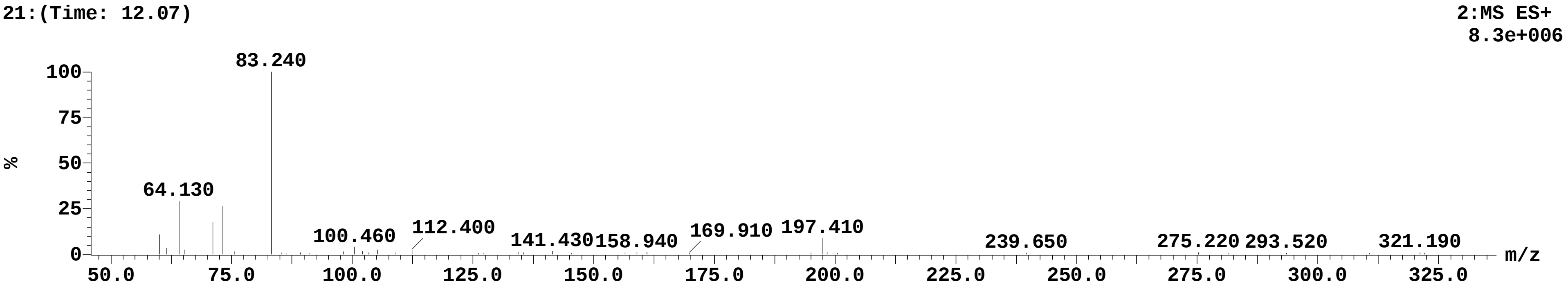

Peak ID	Compound	Time	Mass Found
22		12.36	

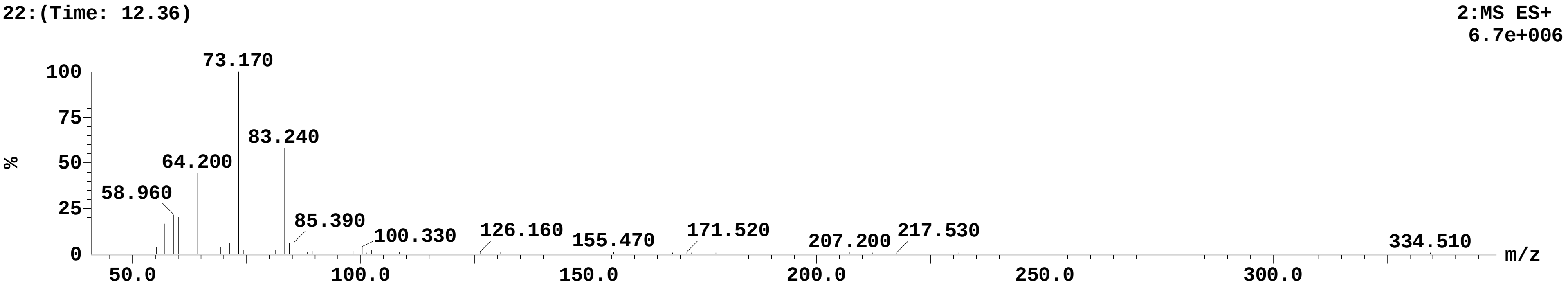

Sample: 643 Vial:1:B,4 ID:
File:LK-2 Date:03-Aug-2023 Time:12:54:05
Description:Default file

Printed: Thu Aug 03 15:05:03 2023

Sample Report (continued):

Peak ID	Compound	Time	Mass Found
23		13.03	

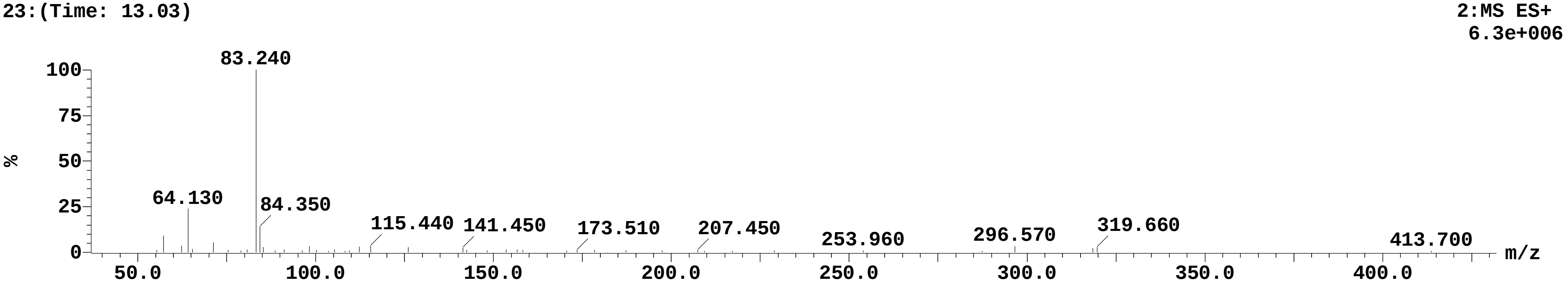

Peak ID	Compound	Time	Mass Found
24		14.35	

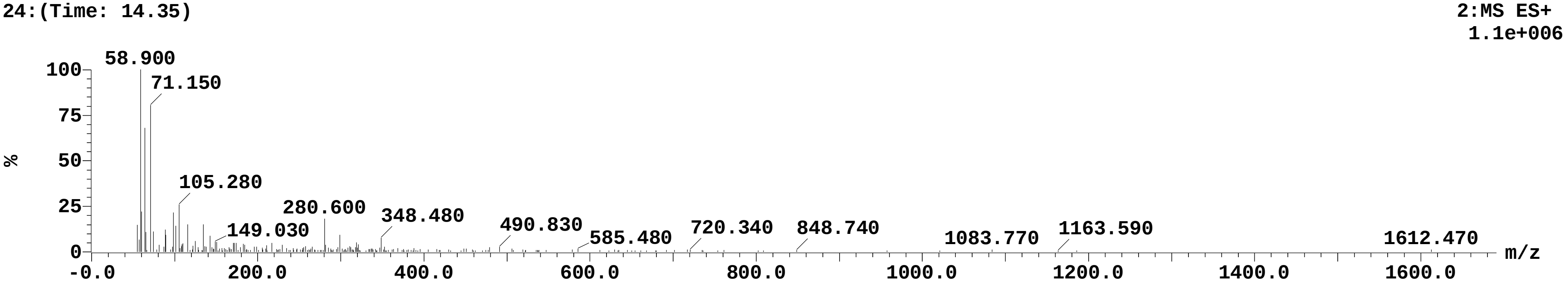

Sample: 643 Vial:1:B,4 ID:
File:LK-2 Date:03-Aug-2023 Time:12:54:05
Description:Default file

Printed: Thu Aug 03 15:05:03 2023

Sample Report (continued):

Peak ID	Compound	Time	Mass Found
25		15.48	

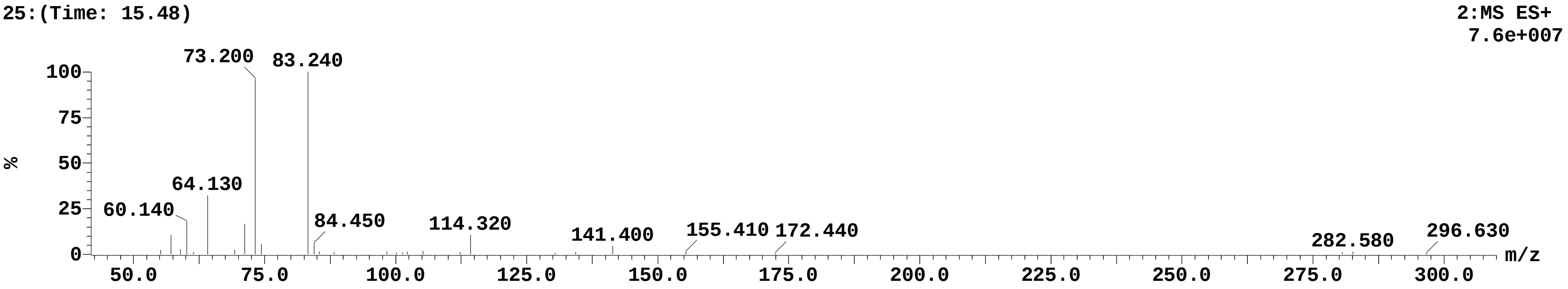

Peak ID	Compound	Time	Mass Found
26		15.65	

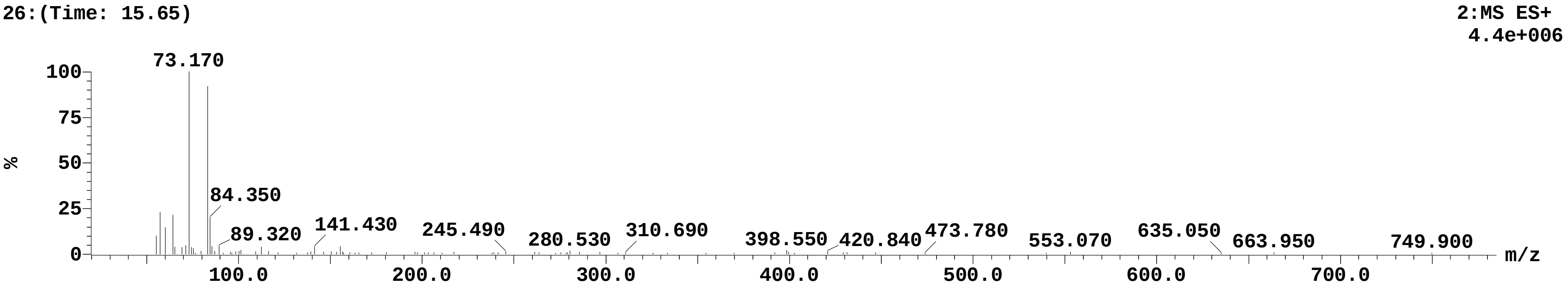

Sample: 643 Vial:1:B,4 ID:
File:LK-2 Date:03-Aug-2023 Time:12:54:05
Description:Default file

Printed: Thu Aug 03 15:05:03 2023

Sample Report (continued):

Peak ID	Compound	Time	Mass Found
27		16.37	

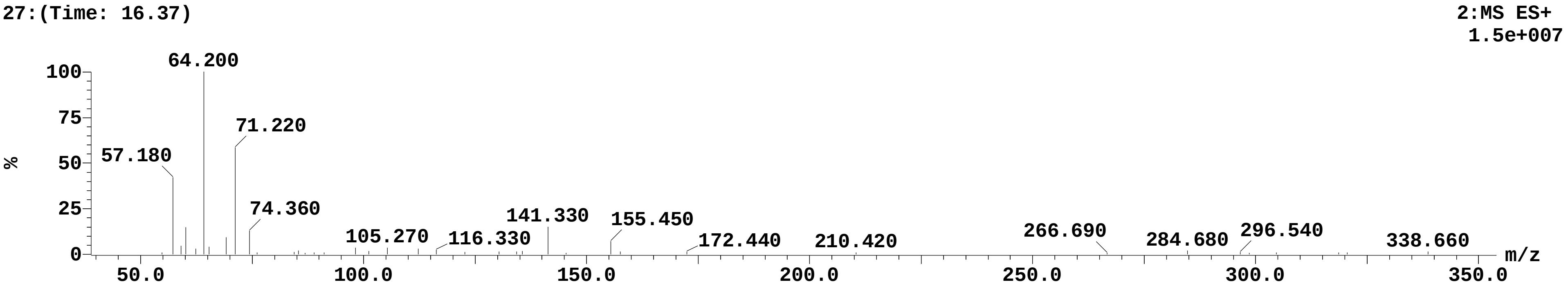

Peak ID	Compound	Time	Mass Found
28		16.48	

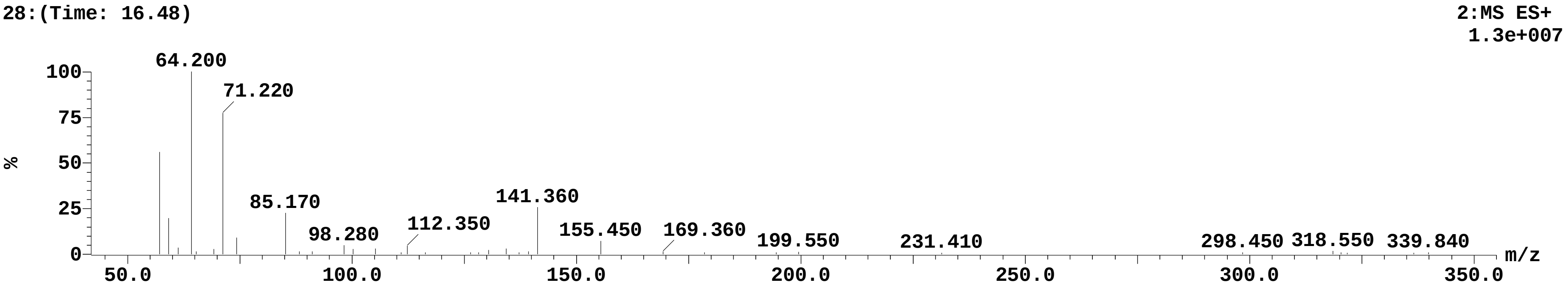

Sample Report (continued):

Peak ID	Compound	Time	Mass Found
29		16.88	

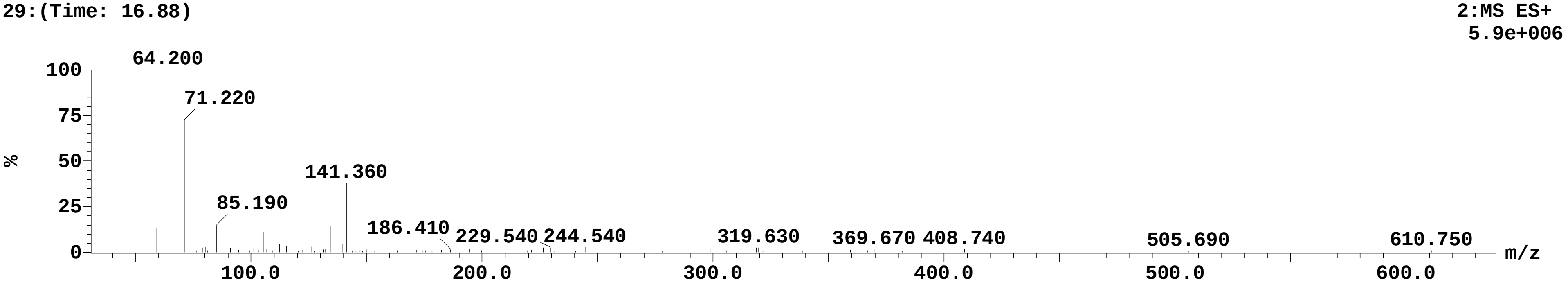

Peak ID	Compound	Time	Mass Found
30		17.20	

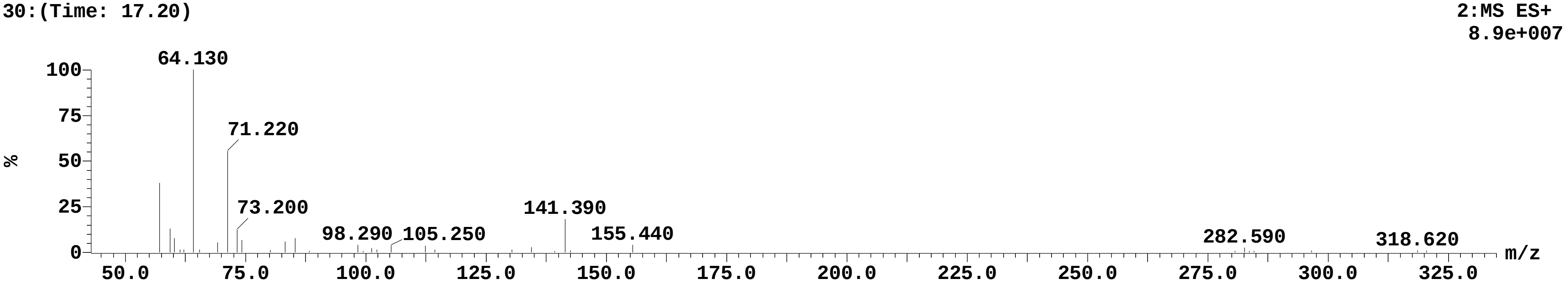

Sample: 643 Vial:1:B,4 ID:
File:LK-2 Date:03-Aug-2023 Time:12:54:05
Description:Default file

Printed: Thu Aug 03 15:05:03 2023

Sample Report (continued):

Peak ID	Compound	Time	Mass Found
32		21.77	

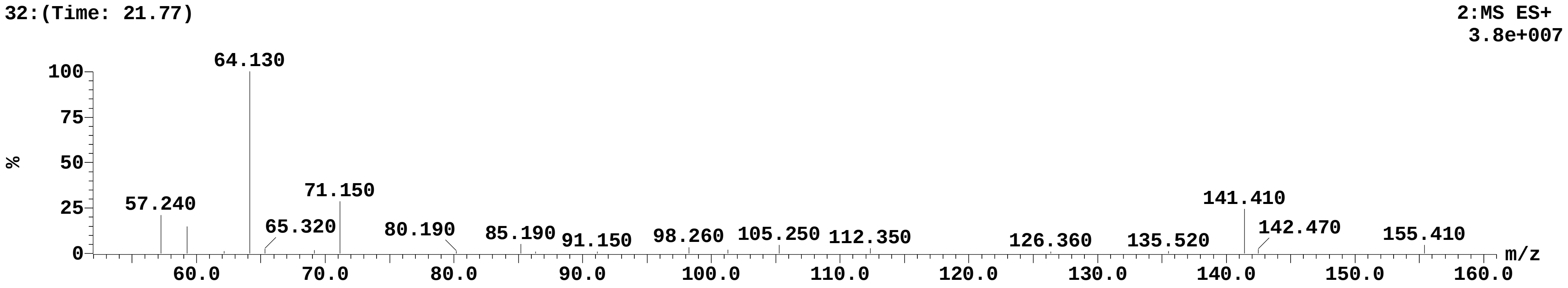

Sample: 644 Vial:1:B,5 ID:
File:LK-3 Date:03-Aug-2023 Time:13:20:09
Description:Default file

Printed: Thu Aug 03 15:05:46 2023

Sample Report:

Sample 644 Vial 1:B,5 ID File LK-3 Date 03-Aug-2023 Time 13:20:09 Description Default file

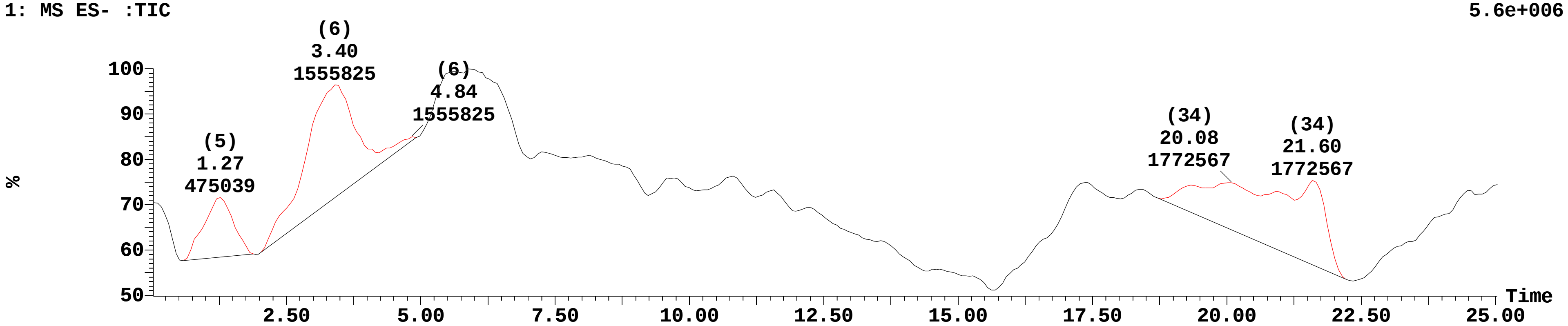

Peak Number	Compound	Time	AreaAbs	Area %Total	Width	Height	Mass Found
5		1.27	5e+005	12.49	1	7e+005	
6		3.40	2e+006	40.91	3	1e+006	
34		21.60	2e+006	46.60	4	1e+006	

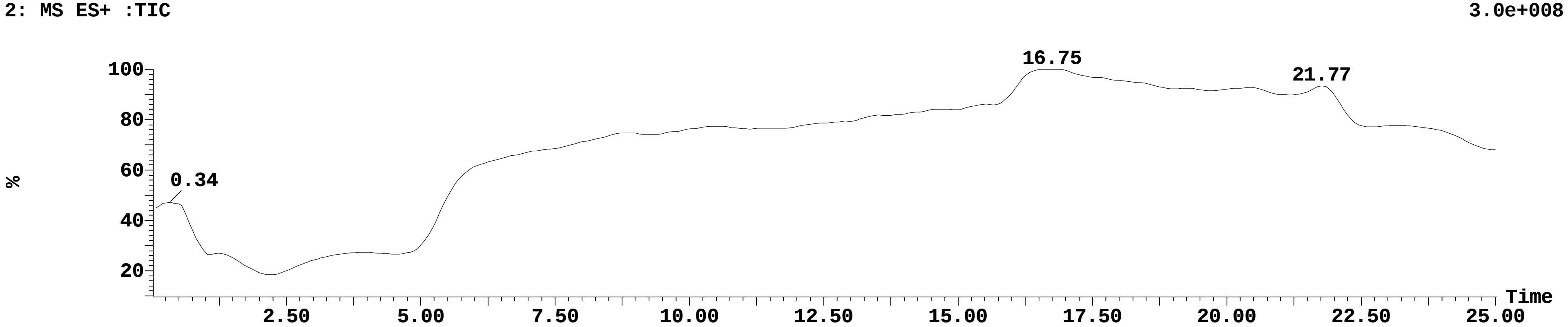

Peak Number	Compound	Time	AreaAbs	Area %Total	Width	Height	Mass Found

Sample: 644 | Vial:1:B,5 | ID:
File:LK-3 | Date:03-Aug-2023 | Time:13:20:09
Description:Default file

Printed: Thu Aug 03 15:05:46 2023

Sample Report (continued):

3: UV Detector: 214 5.591e-2
Range: 9.466e-2

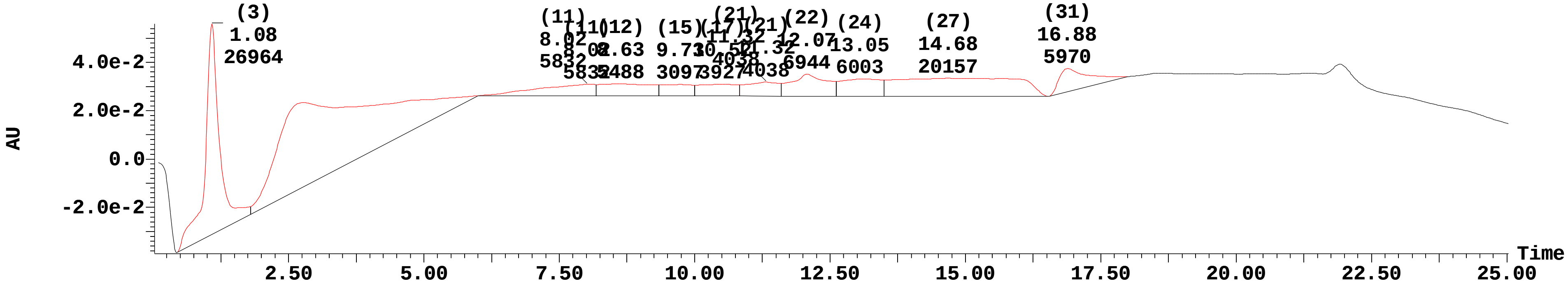

Peak Number	Compound	Time	AreaAbs	Area %Total	Width	Height	Mass Found
3		1.08	3e+004	16.80	1	9e+004	
10		6.00	7e+004	44.91	4		
11		8.02	6e+003	3.63	2	5e+003	
12		8.63	5e+003	3.42	1	5e+003	
15		9.73	3e+003	1.93	1	5e+003	
17		10.52	4e+003	2.45	1	5e+003	
21		11.32	4e+003	2.52	1	6e+003	
22		12.07	7e+003	4.33	1	9e+003	
24		13.07	6e+003	3.74	1	7e+003	
27		14.68	2e+004	12.56	3	7e+003	
31		16.88	6e+003	3.72	1	1e+004	

Sample: 644 Vial:1:B,5 ID:
File:LK-3 Date:03-Aug-2023 Time:13:20:09
Description:Default file

Printed: Thu Aug 03 15:05:46 2023

Sample Report (continued):

3: UV Detector: 254

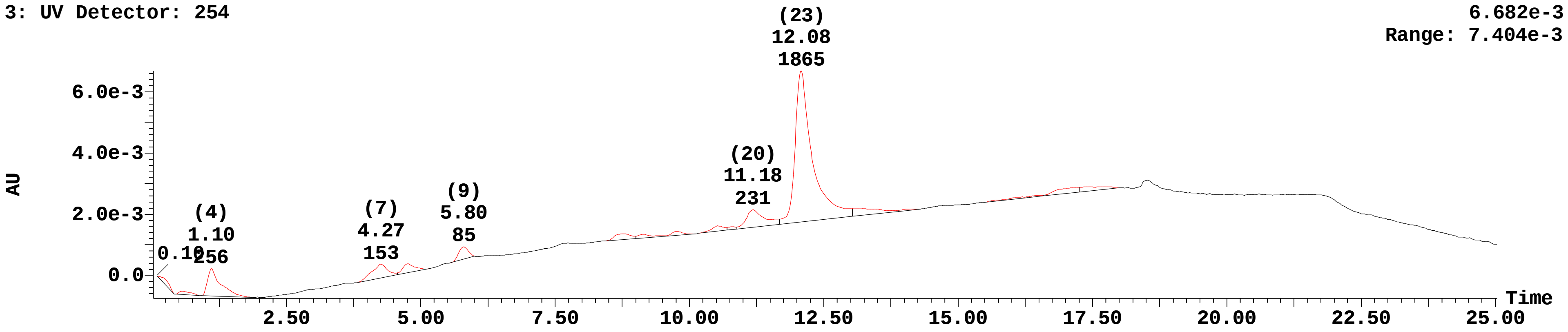

Peak Number	Compound	Time	AreaAbs	Area %Total	Width	Height	Mass Found
1		0.10	3e+001	0.97	0		
2		0.57	3e+001	0.89	0	1e+002	
4		1.10	3e+002	7.90	1	9e+002	
7		4.27	2e+002	4.73	1	4e+002	
8		4.75	8e+001	2.35	1	3e+002	
9		5.80	8e+001	2.62	0	4e+002	
13		8.78	7e+001	2.04	1	2e+002	
14		9.15	3e+001	1.02	1	1e+002	
16		9.77	3e+001	0.87	1	1e+002	
18		10.53	5e+001	1.48	1	2e+002	
19		10.78	2e+001	0.50	0	1e+002	
20		11.18	2e+002	7.13	1	6e+002	
23		12.08	2e+003	57.66	1	5e+003	
25		13.13	1e+002	3.87	1	3e+002	
26		14.30	1e+001	0.34	0		
28		15.80	5e+000	0.15	0	1e+001	
29		16.28	1e+001	0.45	0	2e+001	
30		16.58	9e+000	0.27	0	2e+001	
32		17.27	9e+001	2.66	1	1e+002	
33		17.40	7e+001	2.10	1	1e+002	

Sample: 644 Vial:1:B,5 ID:
File:LK-3 Date:03-Aug-2023 Time:13:20:09
Description:Default file

Printed: Thu Aug 03 15:05:46 2023

Sample Report (continued):

Peak ID	Compound	Time	Mass Found
1		0.10	

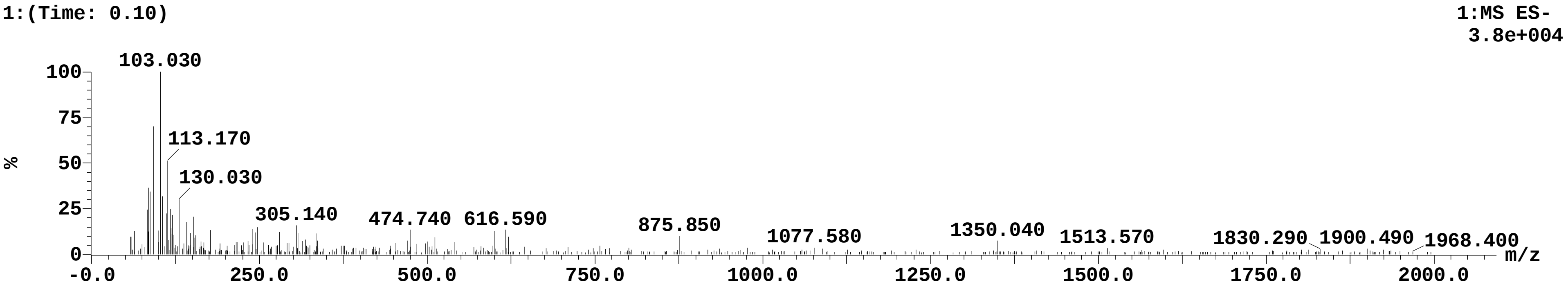

Peak ID	Compound	Time	Mass Found
2		0.57	

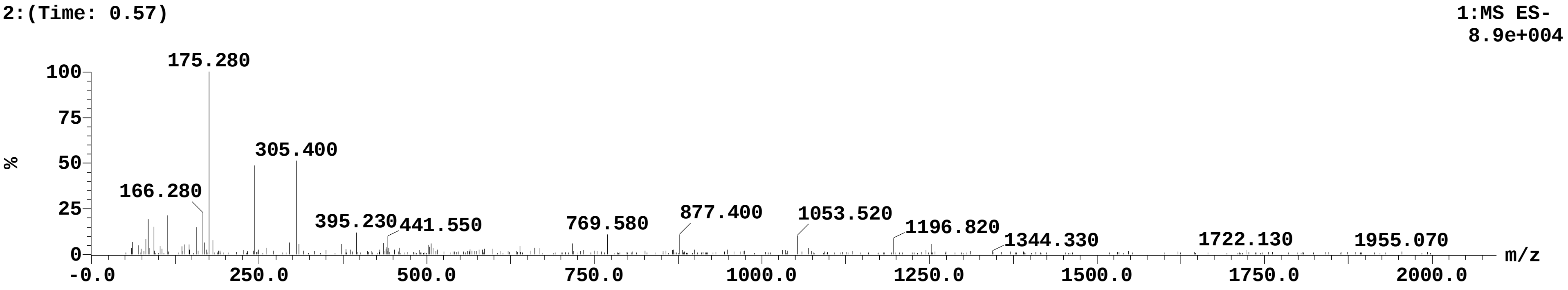

Sample: 644 Vial:1:B,5 ID:
File:LK-3 Date:03-Aug-2023 Time:13:20:09
Description:Default file

Printed: Thu Aug 03 15:05:46 2023

Sample Report (continued):

Peak ID	Compound	Time	Mass Found
3		1.08	

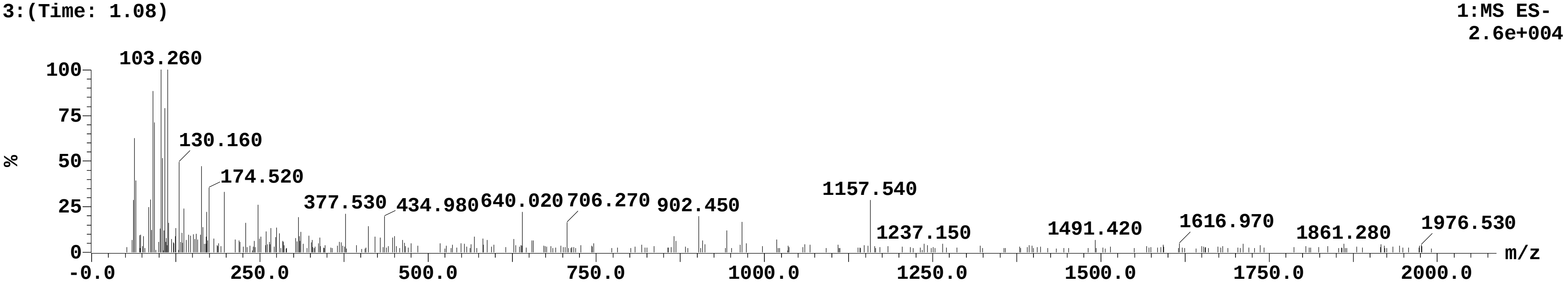

Peak ID	Compound	Time	Mass Found
4		1.10	

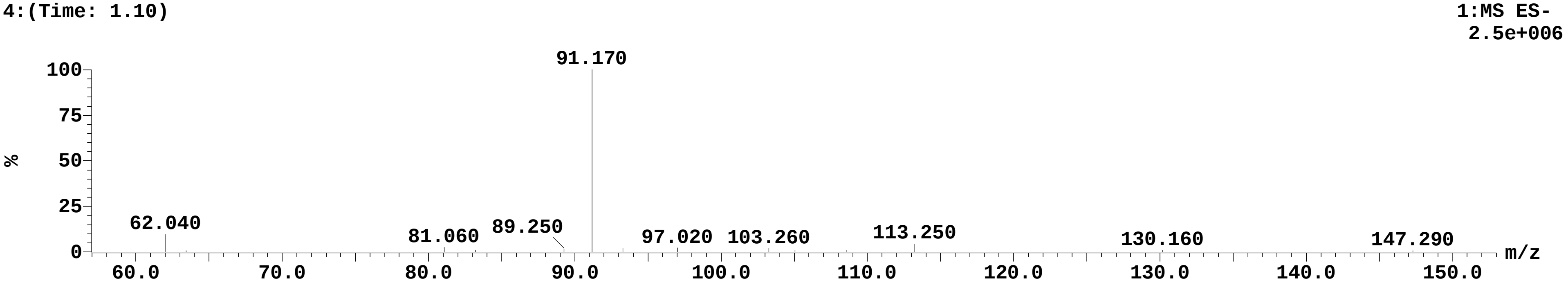

Sample Report (continued):

Peak ID	Compound	Time	Mass Found
5		1.27	

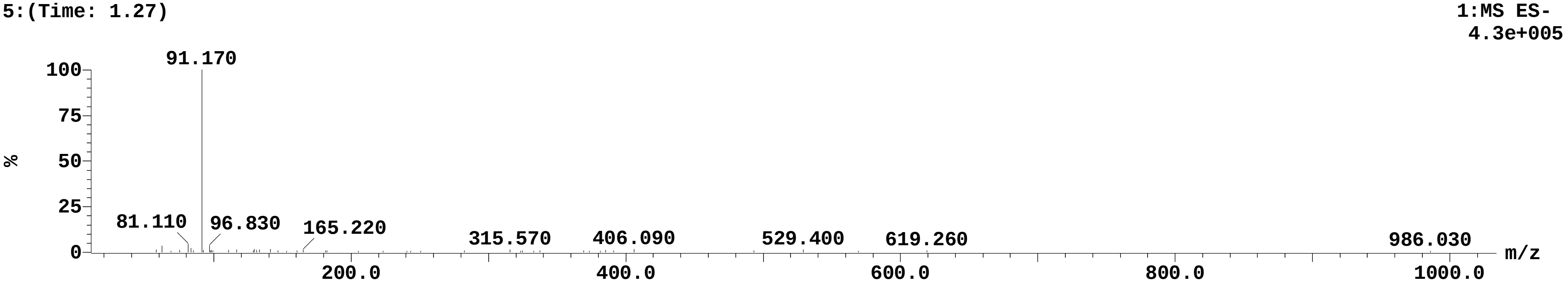

Peak ID	Compound	Time	Mass Found
6		3.40	

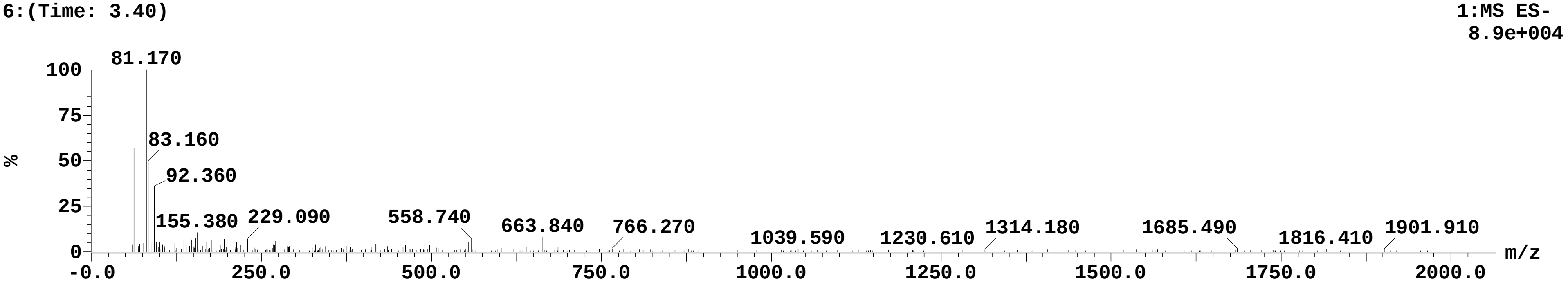

Sample: 644 Vial:1:B,5 ID:
File:LK-3 Date:03-Aug-2023 Time:13:20:09
Description:Default file

Printed: Thu Aug 03 15:05:46 2023

Sample Report (continued):

Peak ID	Compound	Time	Mass Found
7		4.27	

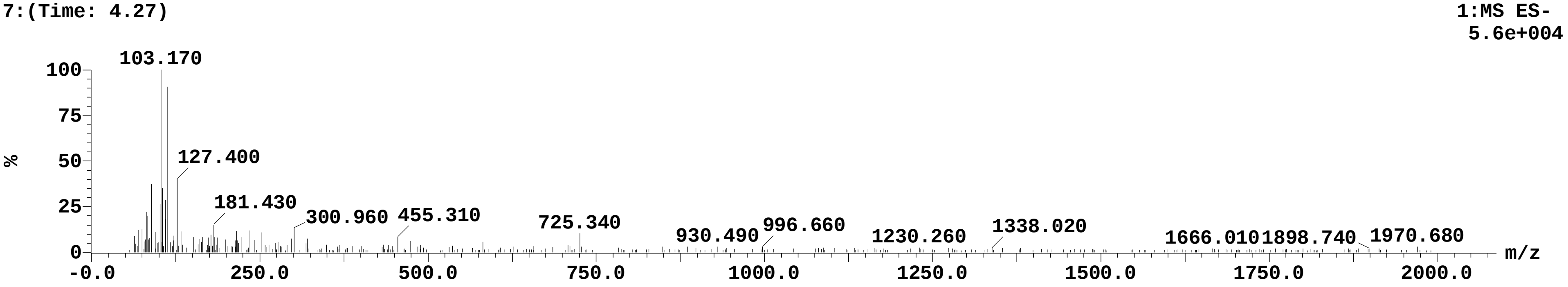

Peak ID	Compound	Time	Mass Found
8		4.75	

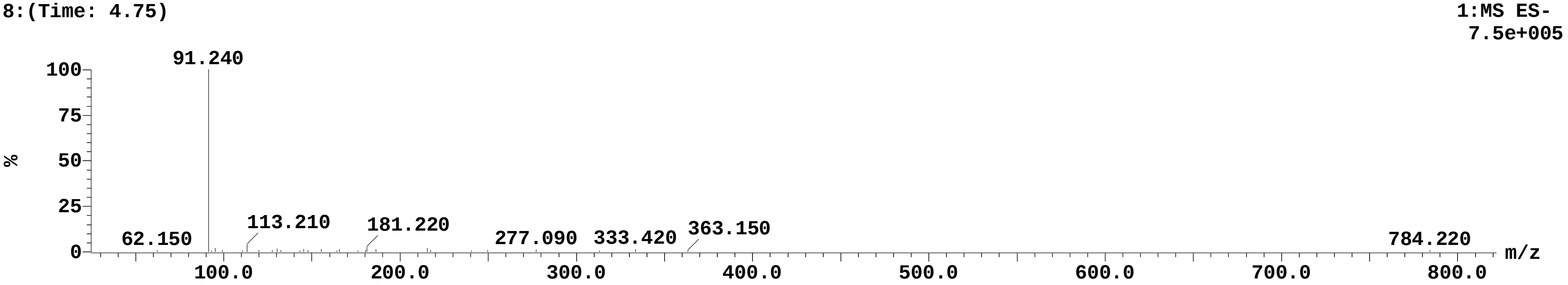

Sample Report (continued):

Peak ID	Compound	Time	Mass Found
9		5.80	

9:(Time: 5.80)

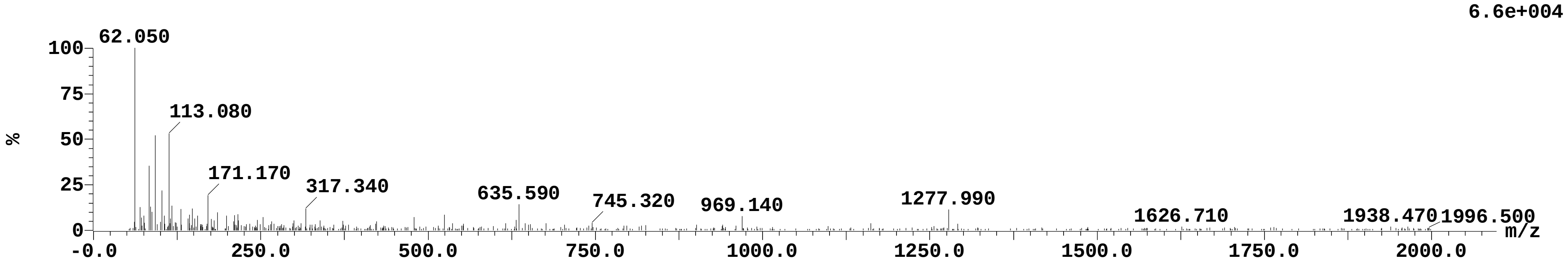

Peak ID	Compound	Time	Mass Found
10		6.00	

10:(Time: 6.00)

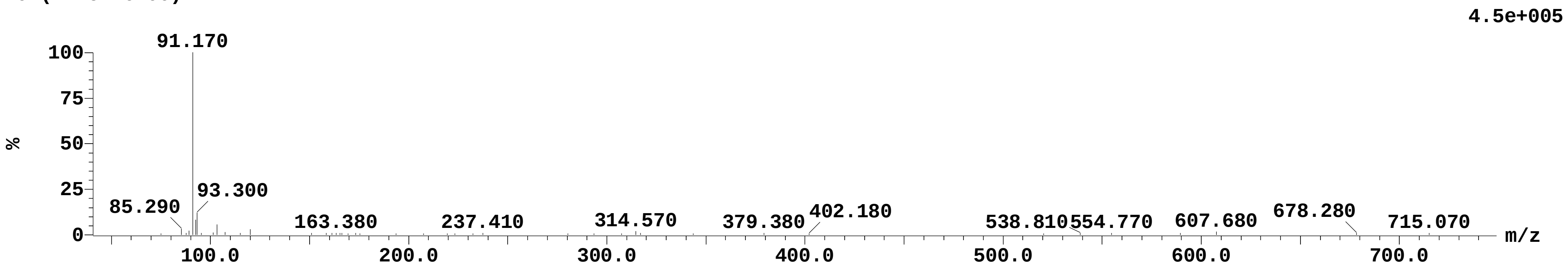

Sample: 644 Vial:1:B,5 ID:
File:LK-3 Date:03-Aug-2023 Time:13:20:09
Description:Default file

Printed: Thu Aug 03 15:05:46 2023

Sample Report (continued):

Peak ID	Compound	Time	Mass Found
11		8.02	

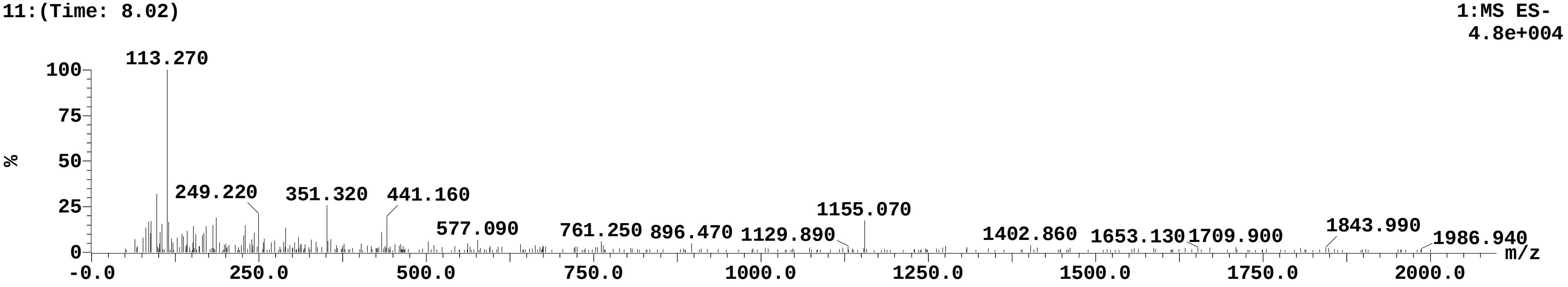

Peak ID	Compound	Time	Mass Found
12		8.63	

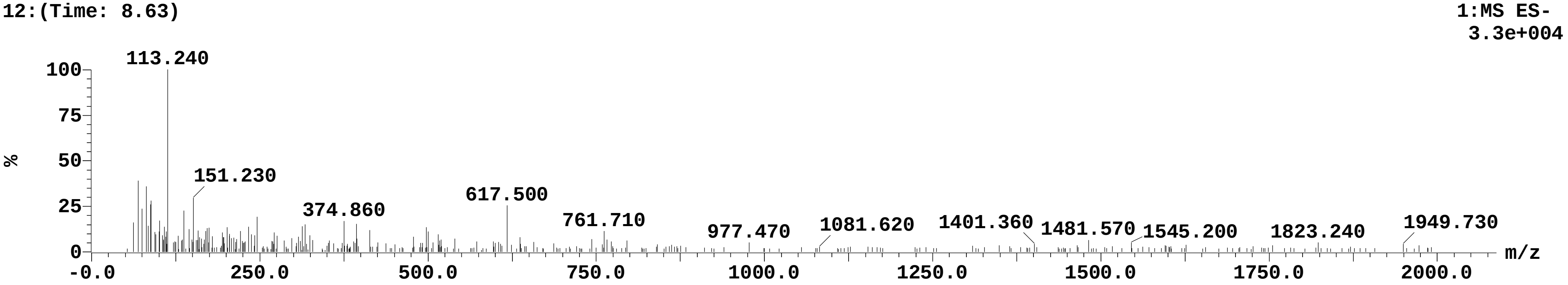

Sample: 644 Vial:1:B,5 ID:
File:LK-3 Date:03-Aug-2023 Time:13:20:09
Description:Default file

Printed: Thu Aug 03 15:05:46 2023

Sample Report (continued):

Peak ID	Compound	Time	Mass Found
13		8.78	

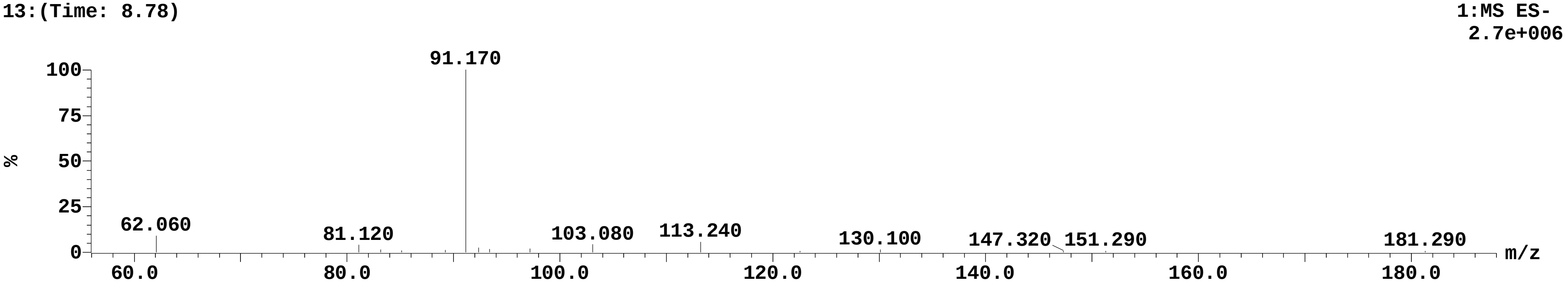

Peak ID	Compound	Time	Mass Found
14		9.15	

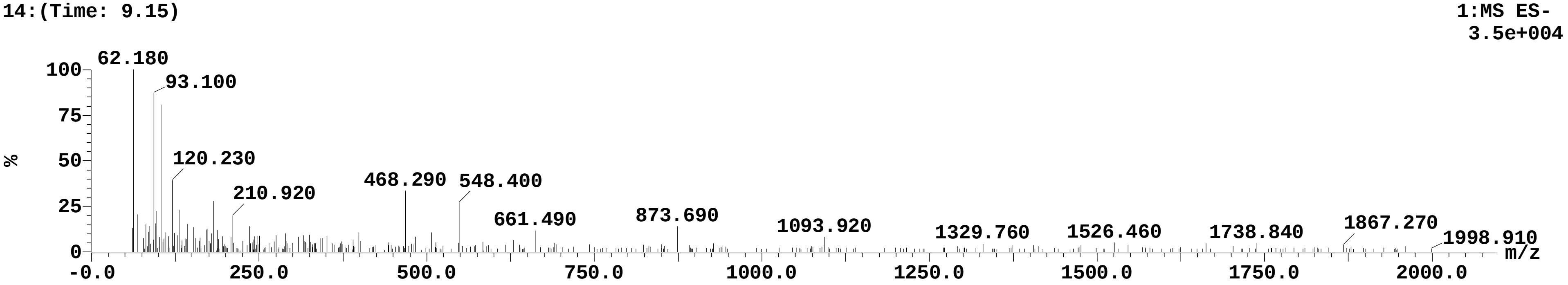

Sample: 644 Vial:1:B,5 ID:
File:LK-3 Date:03-Aug-2023 Time:13:20:09
Description:Default file

Printed: Thu Aug 03 15:05:46 2023

Sample Report (continued):

Peak ID	Compound	Time	Mass Found
15		9.73	

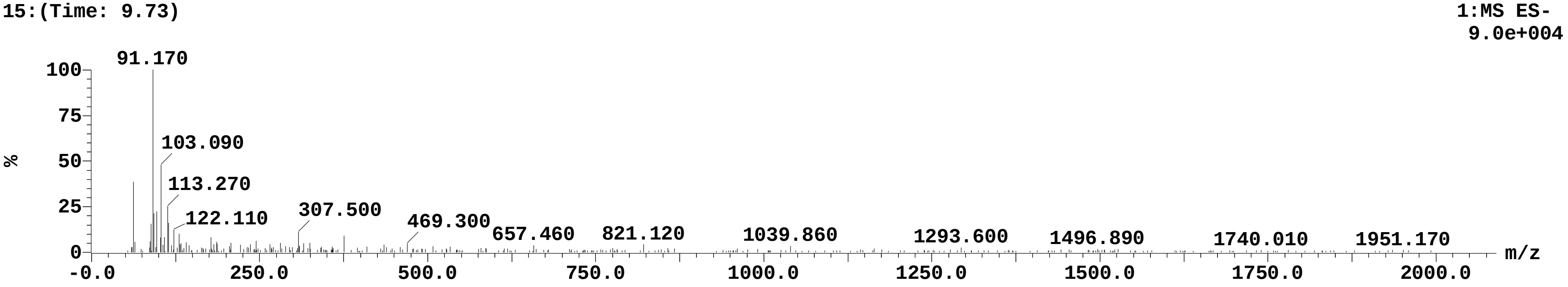

Peak ID	Compound	Time	Mass Found
16		9.77	

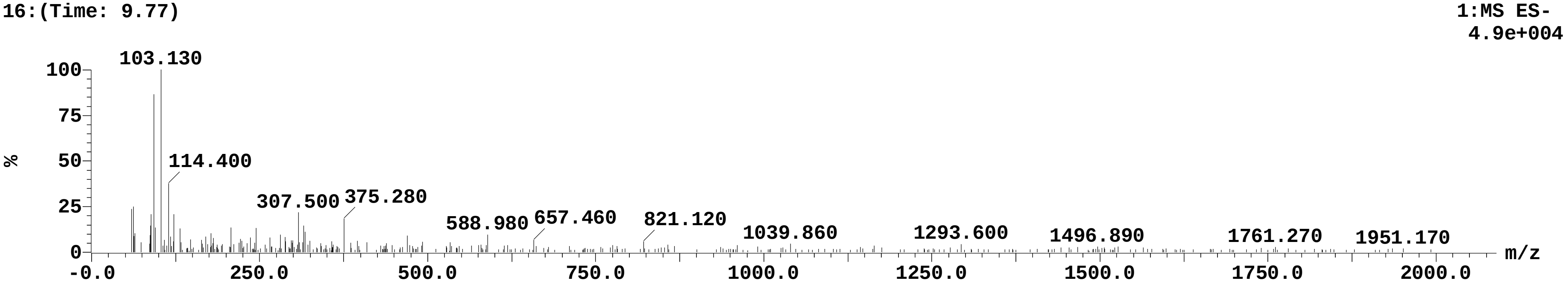

Sample: 644 Vial:1:B,5 ID:
File:LK-3 Date:03-Aug-2023 Time:13:20:09
Description:Default file

Printed: Thu Aug 03 15:05:46 2023

Sample Report (continued):

Peak ID	Compound	Time	Mass Found
17		10.52	

17:(Time: 10.52) 1:MS ES- 4.6e+004

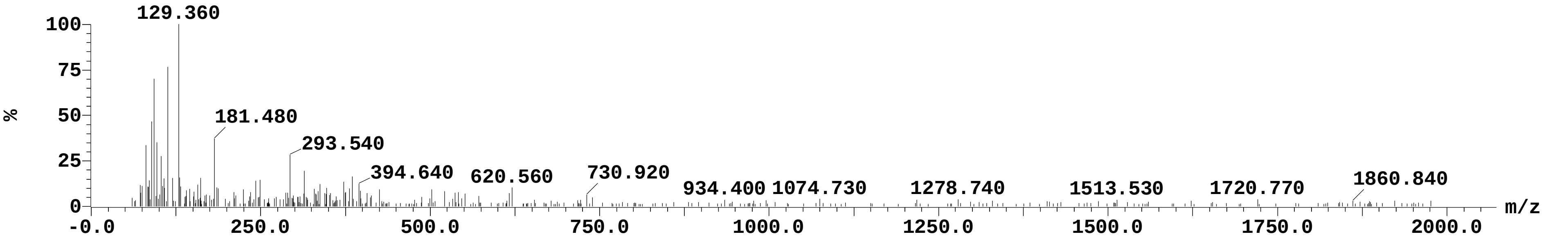

Peak ID	Compound	Time	Mass Found
18		10.53	

18:(Time: 10.53) 1:MS ES- 3.5e+004

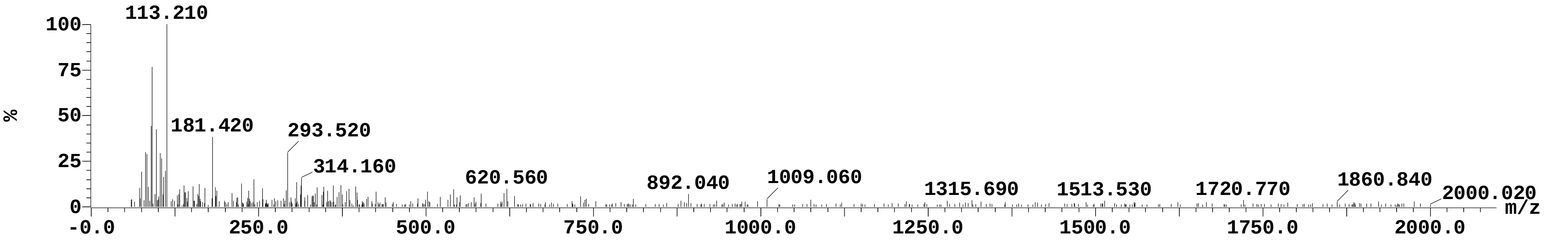

Sample: 644 | Vial:1:B,5 | ID:
File:LK-3 | Date:03-Aug-2023 | Time:13:20:09
Description:Default file

Printed: Thu Aug 03 15:05:46 2023

Sample Report (continued):

Peak ID	Compound	Time	Mass Found
19		10.78	

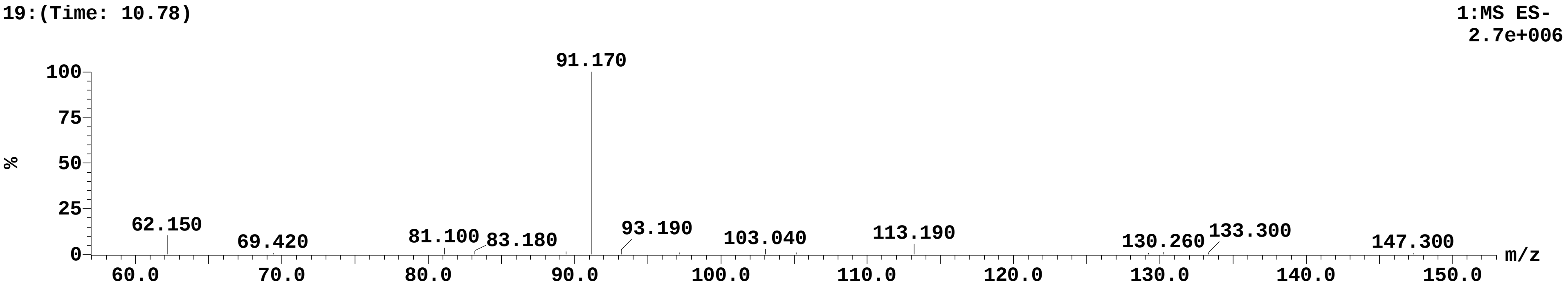

Peak ID	Compound	Time	Mass Found
20		11.18	

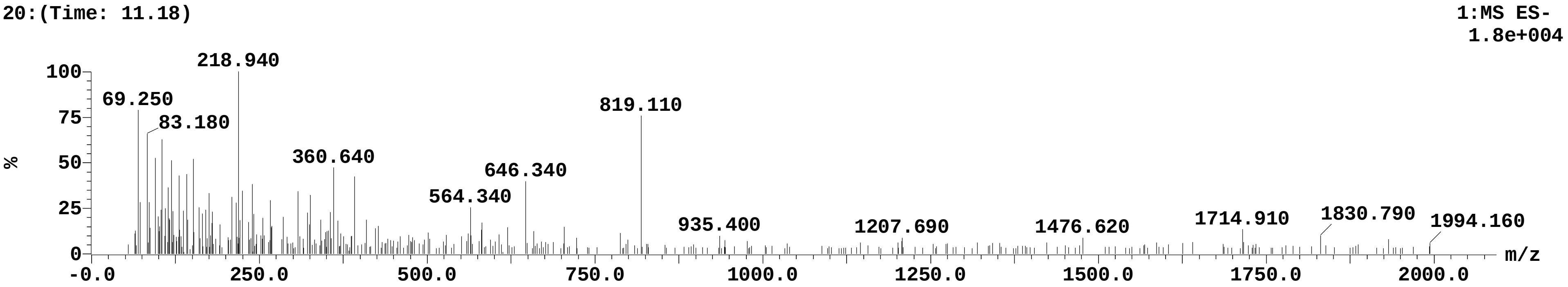

Sample: 644 Vial:1:B,5 ID:
File:LK-3 Date:03-Aug-2023 Time:13:20:09
Description:Default file

Printed: Thu Aug 03 15:05:46 2023

Sample Report (continued):

Peak ID	Compound	Time	Mass Found
21		11.32	

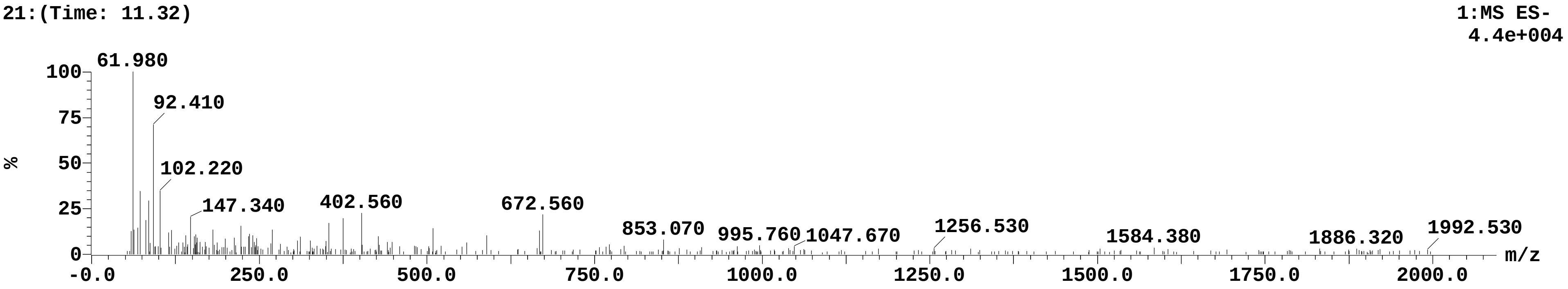

Peak ID	Compound	Time	Mass Found
22		12.07	

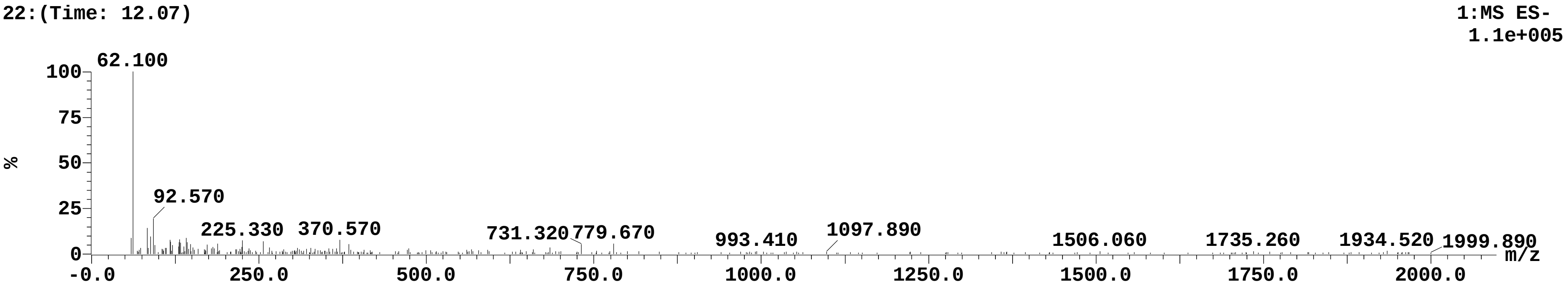

Sample: 644 Vial:1:B,5 ID:
File:LK-3 Date:03-Aug-2023 Time:13:20:09
Description:Default file

Printed: Thu Aug 03 15:05:46 2023

Sample Report (continued):

Peak ID	Compound	Time	Mass Found
23		12.08	

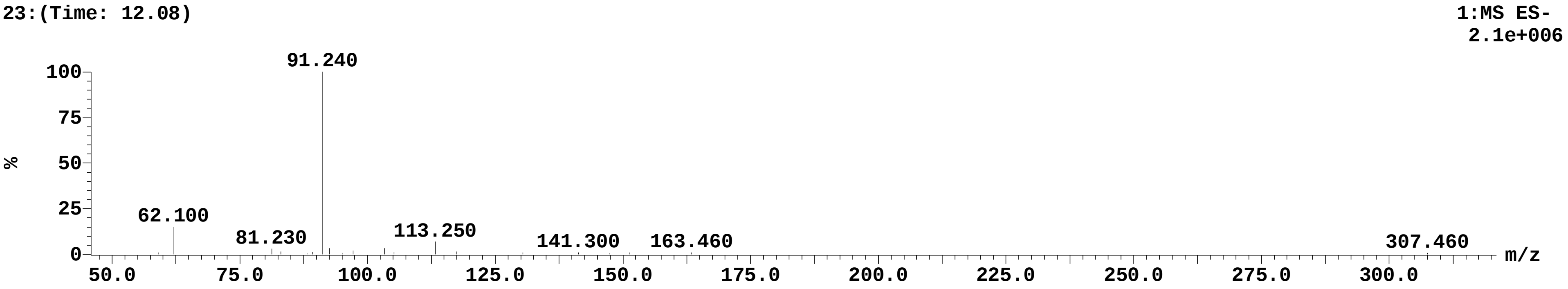

Peak ID	Compound	Time	Mass Found
24		13.07	

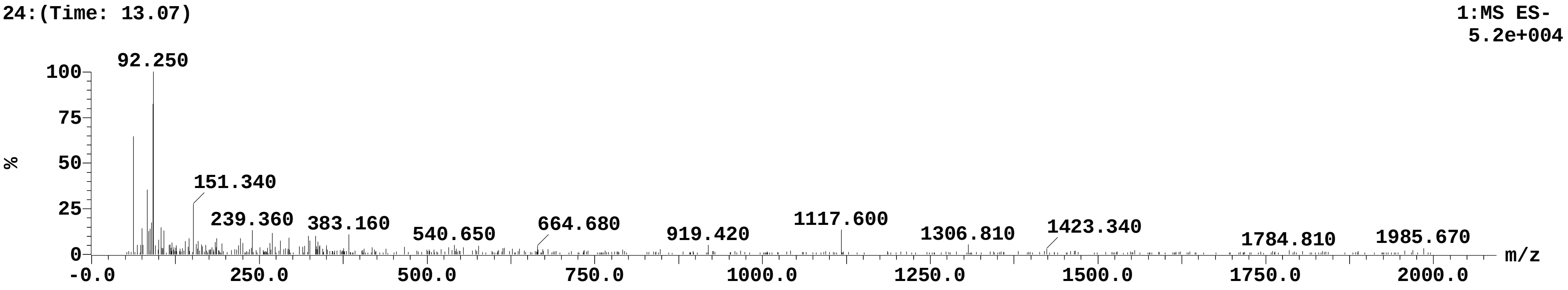

Sample Report (continued):

Peak ID	Compound	Time	Mass Found
25		13.13	

25:(Time: 13.13) 1:MS ES- 8.3e+004

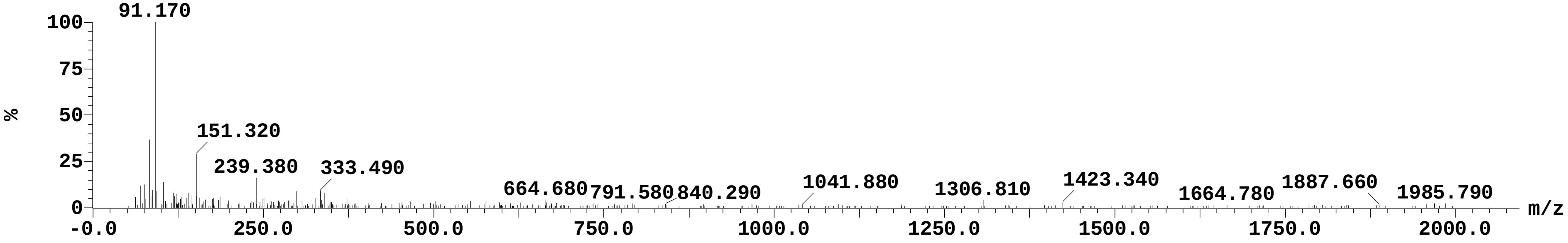

Peak ID	Compound	Time	Mass Found
26		14.30	

26:(Time: 14.30) 1:MS ES- 1.5e+004

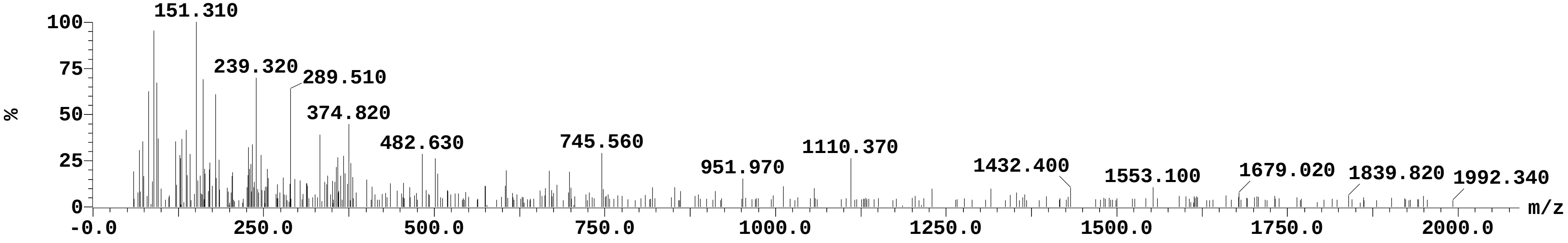

Sample Report (continued):

Peak ID	Compound	Time	Mass Found
27		14.68	

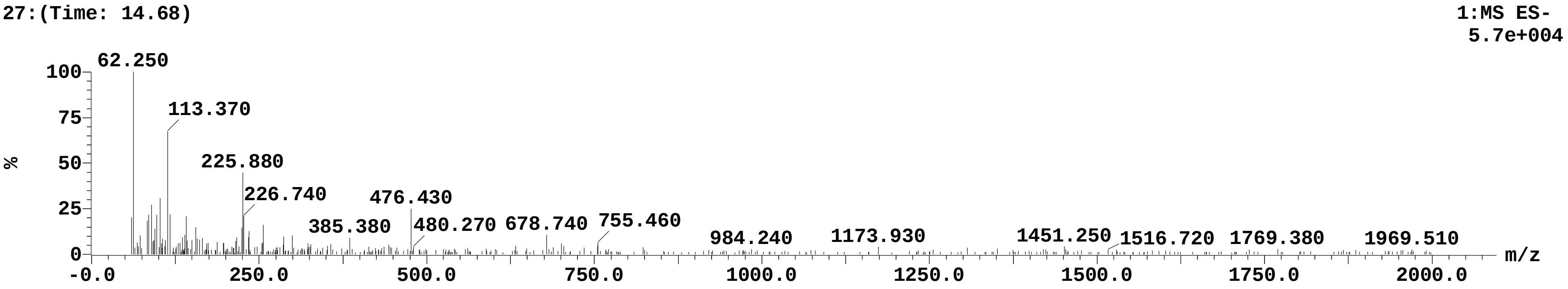

Peak ID	Compound	Time	Mass Found
28		15.80	

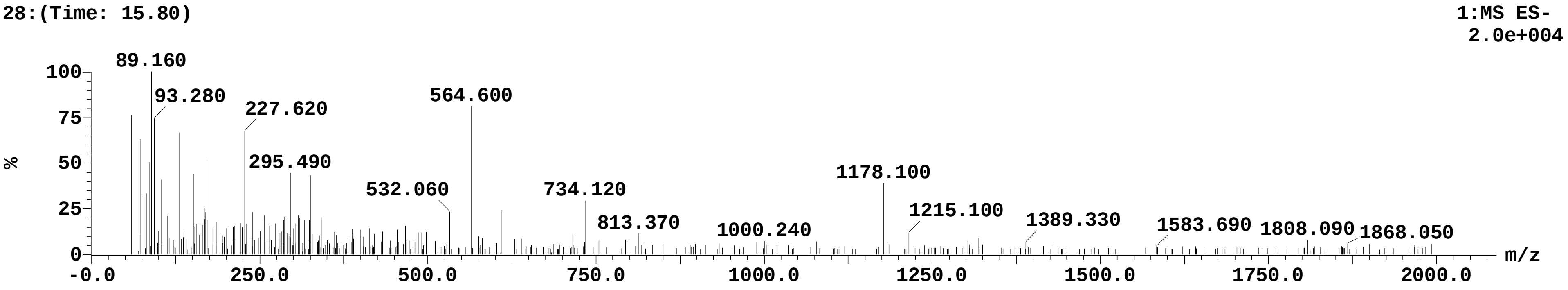

Sample Report (continued):

Peak ID	Compound	Time	Mass Found
29		16.28	

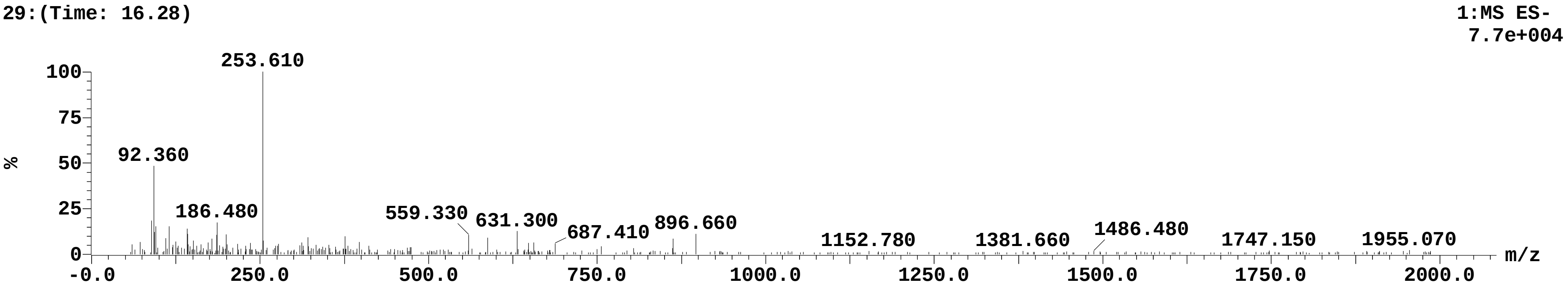

Peak ID	Compound	Time	Mass Found
30		16.58	

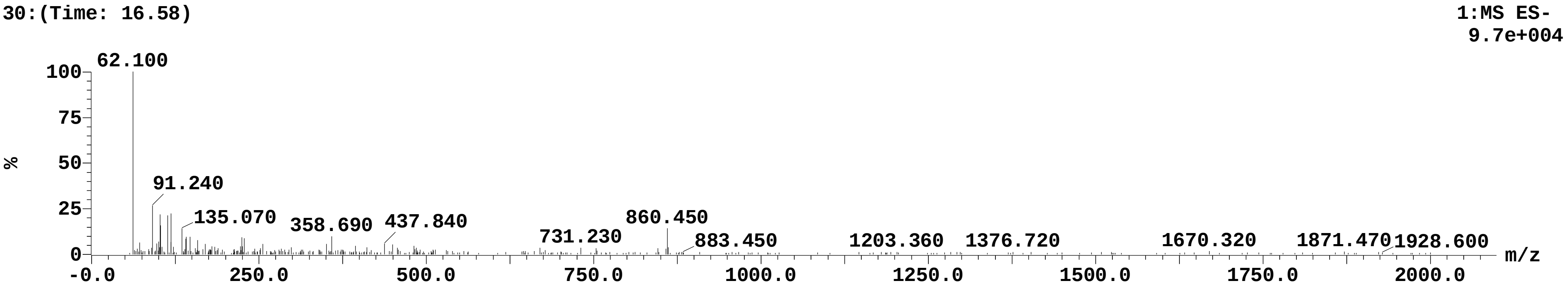

Sample: 644 Vial:1:B,5 ID:
File:LK-3 Date:03-Aug-2023 Time:13:20:09
Description:Default file

Printed: Thu Aug 03 15:05:46 2023

Sample Report (continued):

Peak ID	Compound	Time	Mass Found
31		16.88	

31:(Time: 16.88) 1:MS ES- 2.2e+005

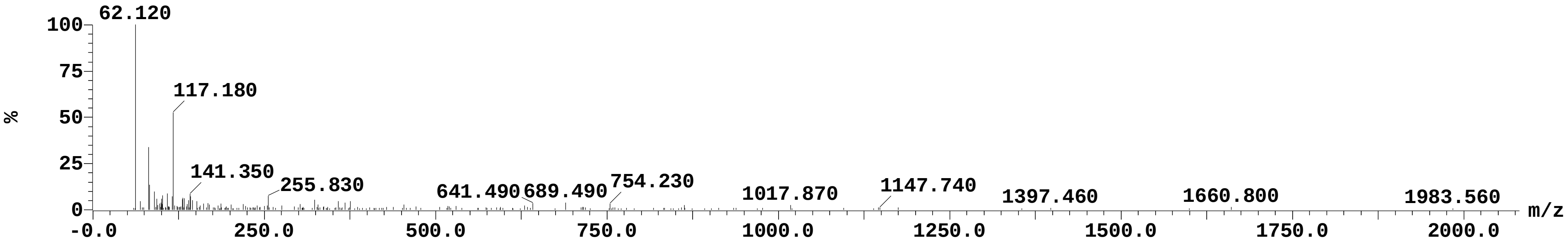

Peak ID	Compound	Time	Mass Found
32		17.27	

32:(Time: 17.27) 1:MS ES- 8.0e+004

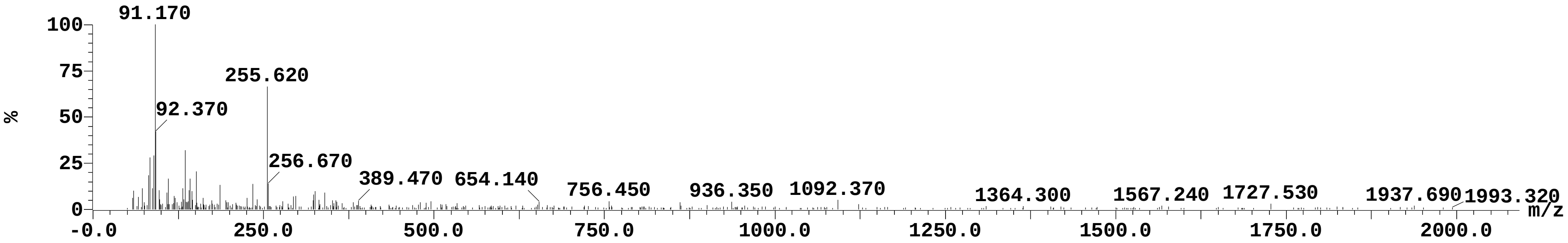

Sample: 644 Vial:1:B,5 ID:
File:LK-3 Date:03-Aug-2023 Time:13:20:09
Description:Default file

Printed: Thu Aug 03 15:05:46 2023

Sample Report (continued):

Peak ID	Compound	Time	Mass Found
33		17.40	

33:(Time: 17.40)

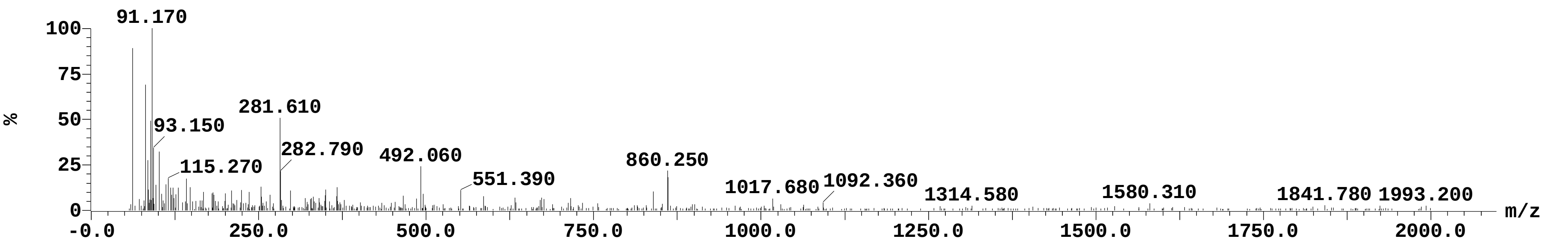

Peak ID	Compound	Time	Mass Found
34		21.60	

34:(Time: 21.60)

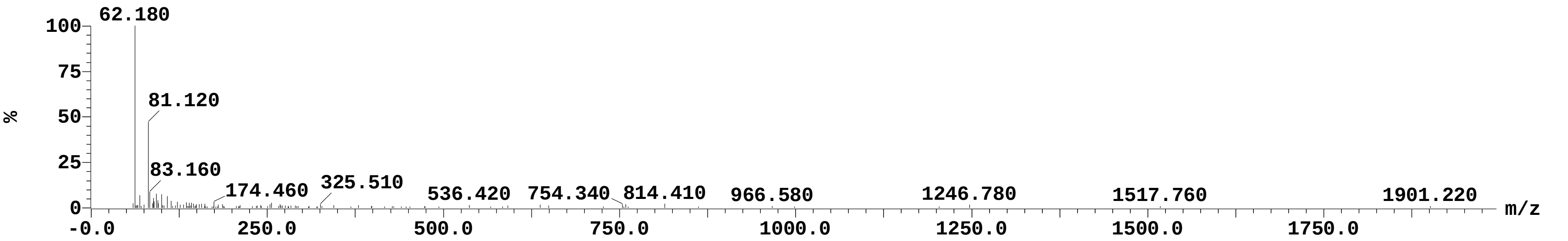

Sample: 644 Vial:1:B,5 ID:
File:LK-3 Date:03-Aug-2023 Time:13:20:09
Description:Default file

Printed: Thu Aug 03 15:05:46 2023

Sample Report (continued):

Peak ID	Compound	Time	Mass Found
1		0.10	

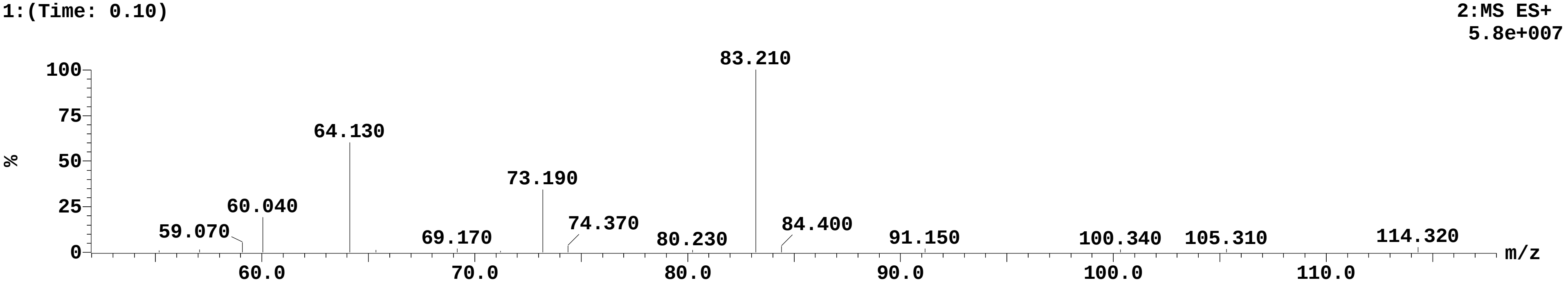

Peak ID	Compound	Time	Mass Found
2		0.57	

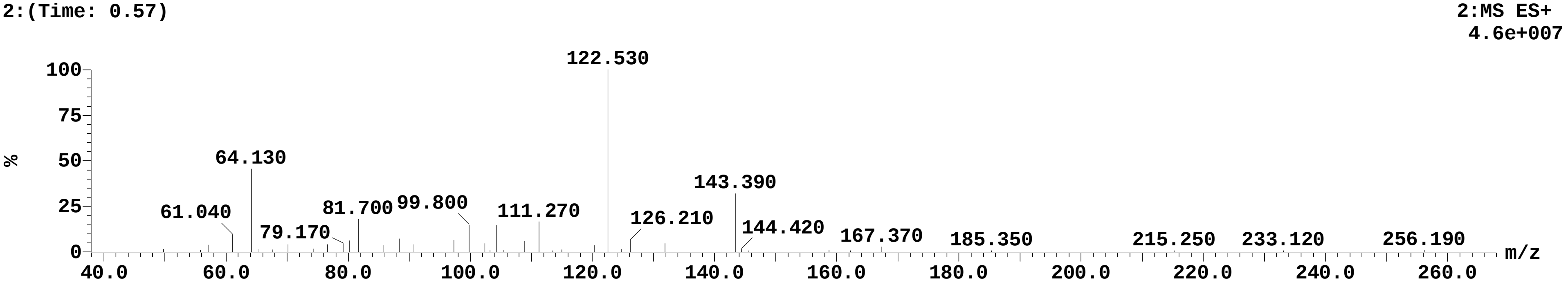

Sample Report (continued):

Peak ID	Compound	Time	Mass Found
3		1.08	

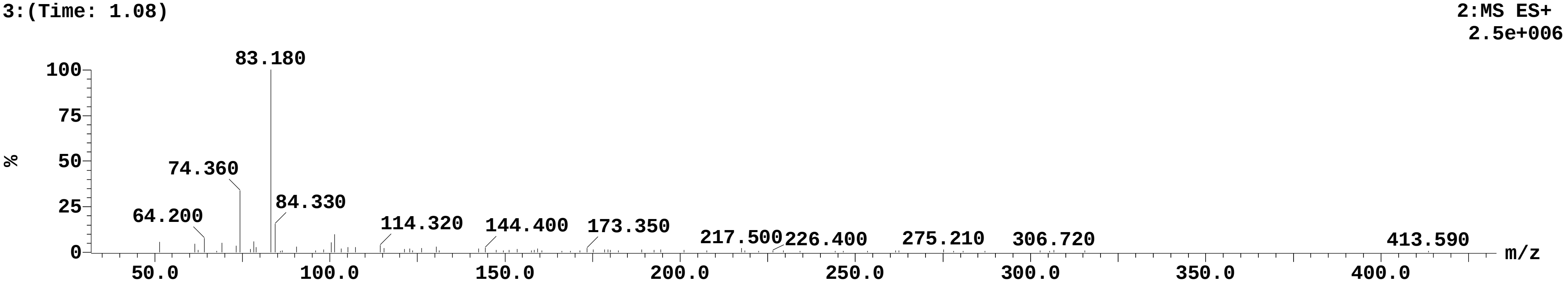

Peak ID	Compound	Time	Mass Found
4		1.10	

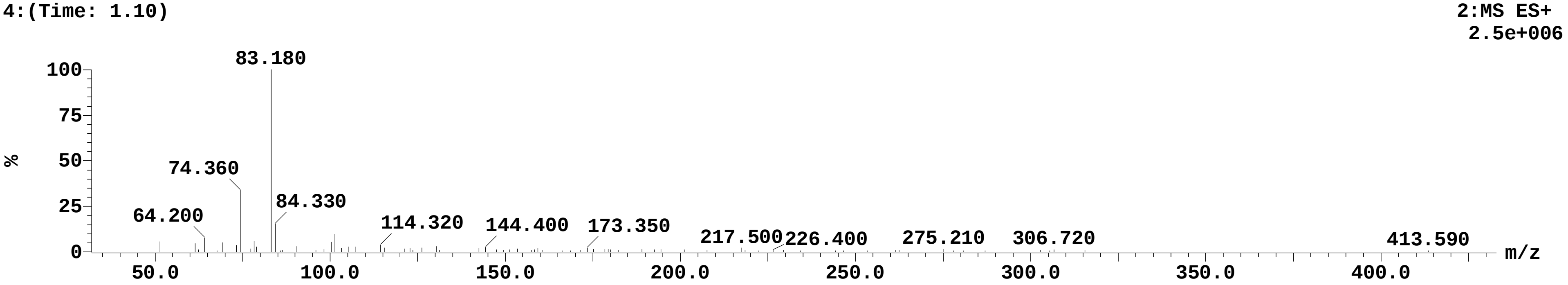

Sample: 644 Vial:1:B,5 ID:
File:LK-3 Date:03-Aug-2023 Time:13:20:09
Description:Default file

Printed: Thu Aug 03 15:05:46 2023

Sample Report (continued):

Peak ID	Compound	Time	Mass Found
7		4.27	

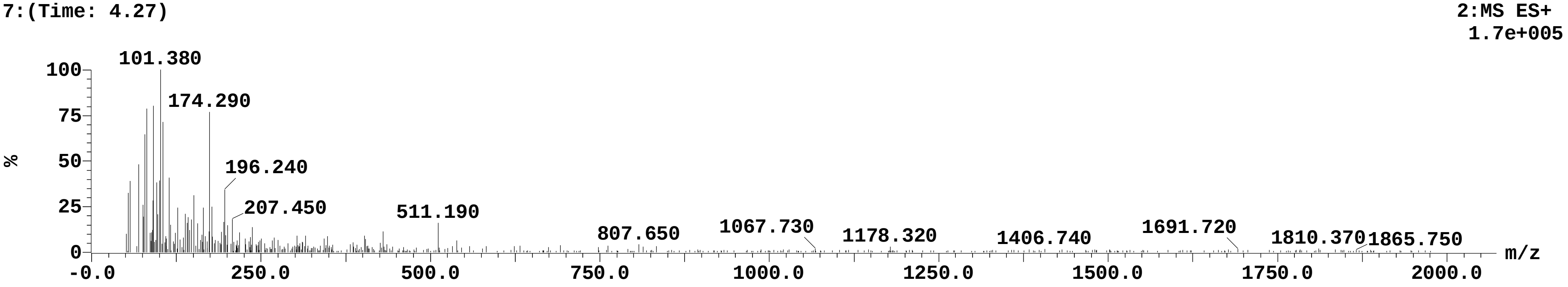

Peak ID	Compound	Time	Mass Found
8		4.75	

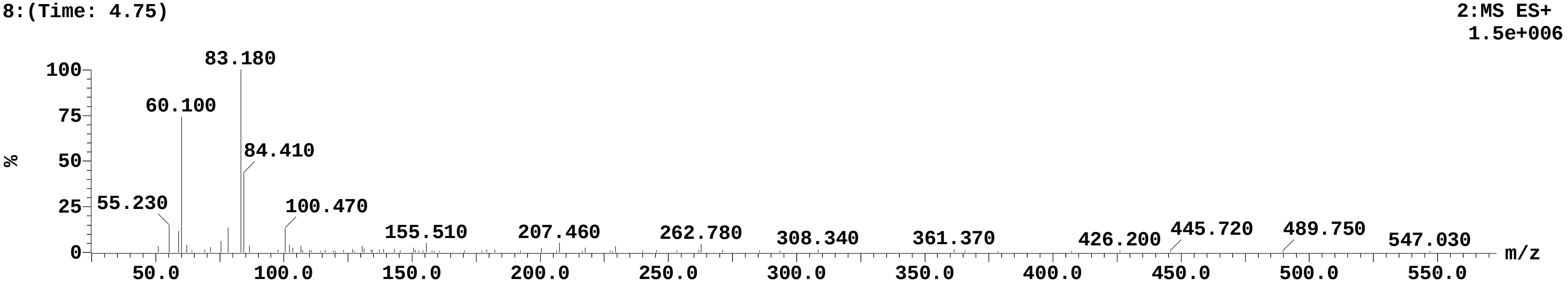

Sample Report (continued):

Peak ID	Compound	Time	Mass Found
9		5.80	

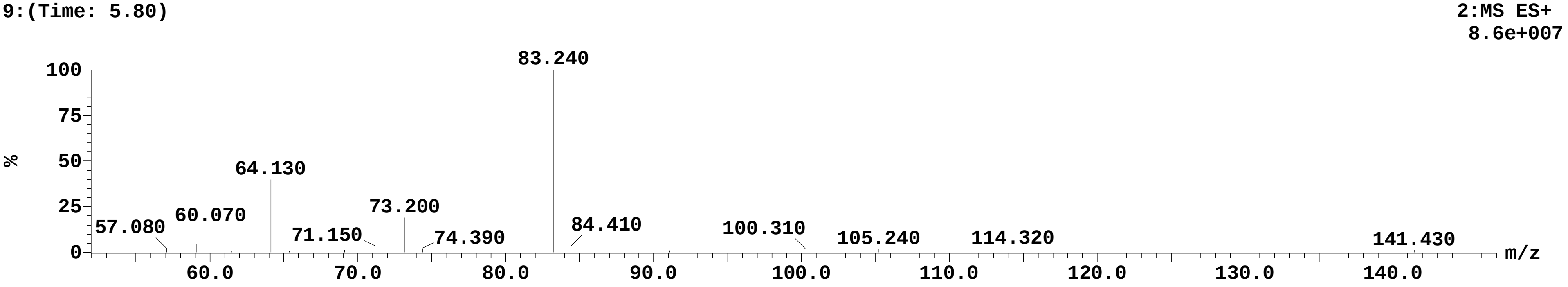

Peak ID	Compound	Time	Mass Found
10		6.00	

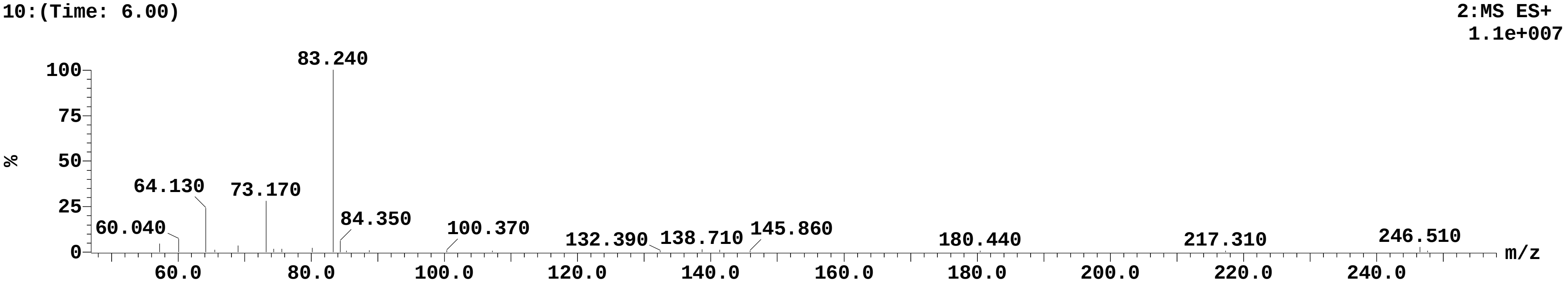

Sample Report (continued):

Peak ID	Compound	Time	Mass Found
11		8.02	

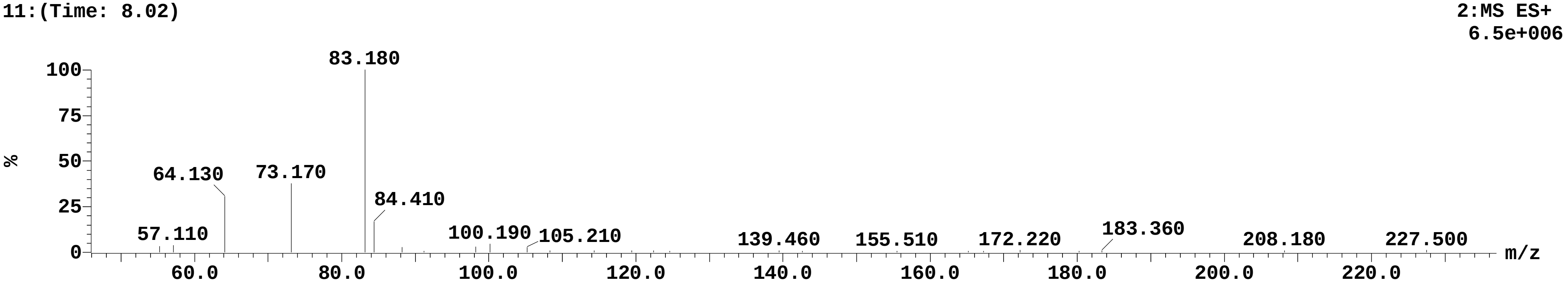

Peak ID	Compound	Time	Mass Found
12		8.63	

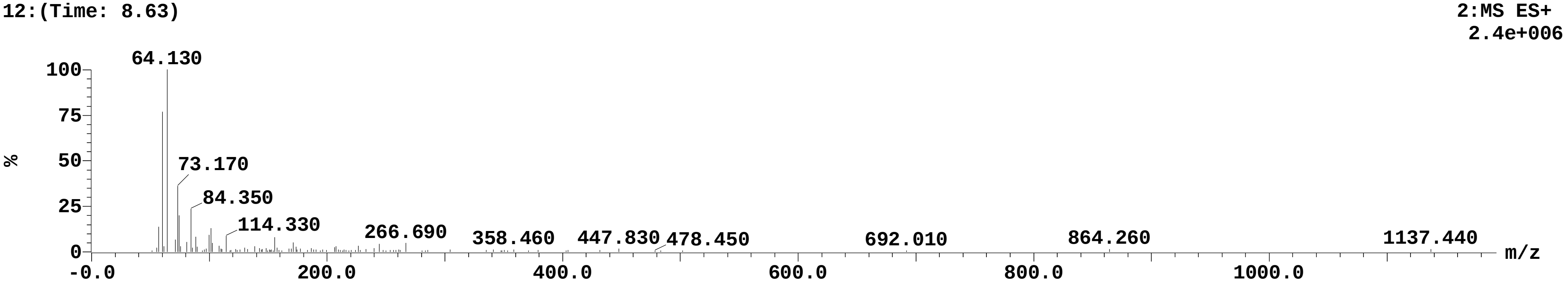

Sample: 644 Vial:1:B,5 ID:
File:LK-3 Date:03-Aug-2023 Time:13:20:09
Description:Default file

Printed: Thu Aug 03 15:05:46 2023

Sample Report (continued):

Peak ID	Compound	Time	Mass Found
13		8.78	

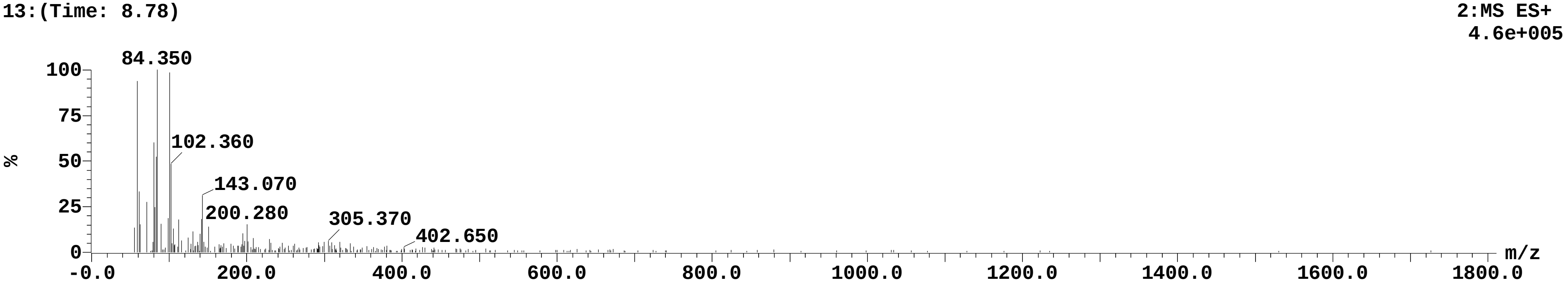

Peak ID	Compound	Time	Mass Found
14		9.15	

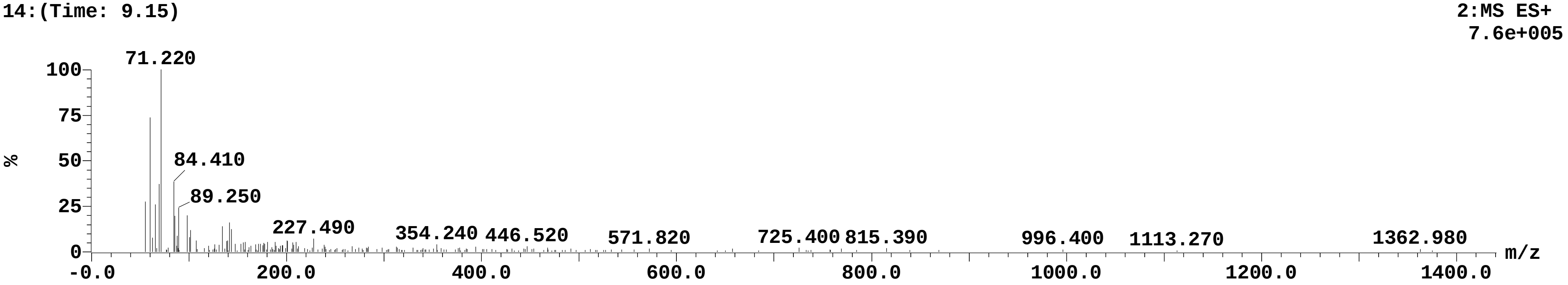

Sample: 644 Vial:1:B,5 ID:
File:LK-3 Date:03-Aug-2023 Time:13:20:09
Description:Default file

Printed: Thu Aug 03 15:05:46 2023

Sample Report (continued):

Peak ID	Compound	Time	Mass Found
15		9.73	

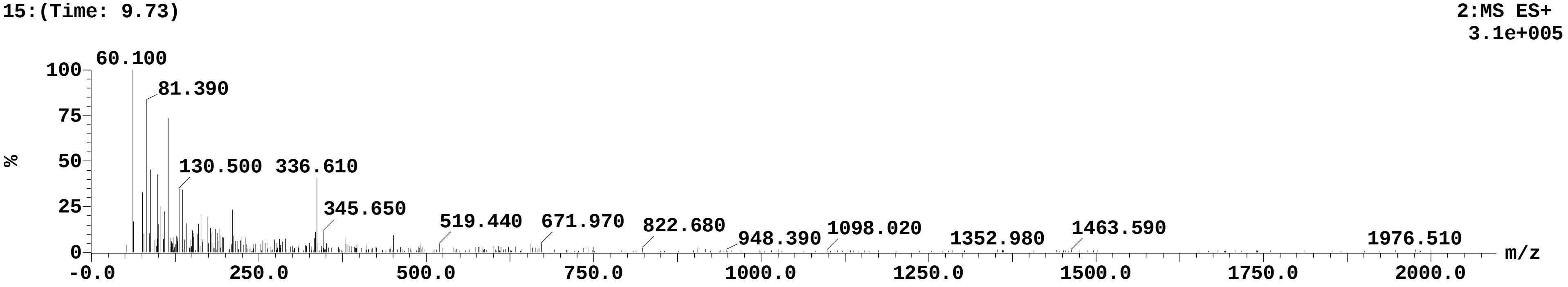

Peak ID	Compound	Time	Mass Found
16		9.77	

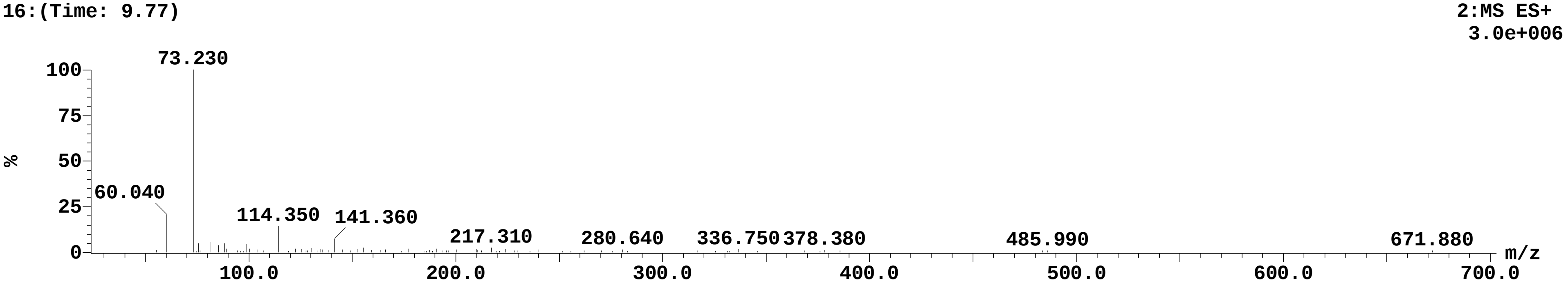

Sample: 644 Vial:1:B,5 ID:
File:LK-3 Date:03-Aug-2023 Time:13:20:09
Description:Default file

Printed: Thu Aug 03 15:05:46 2023

Sample Report (continued):

Peak ID	Compound	Time	Mass Found
17		10.52	

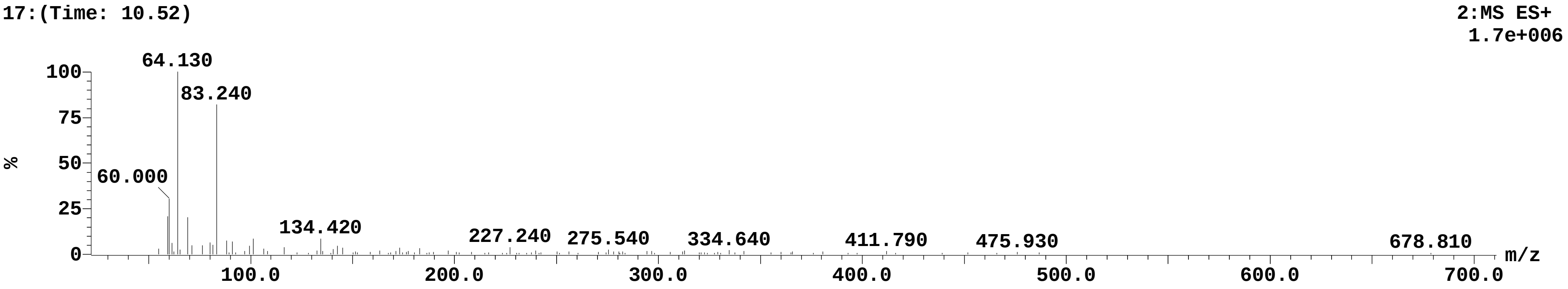

Peak ID	Compound	Time	Mass Found
18		10.53	

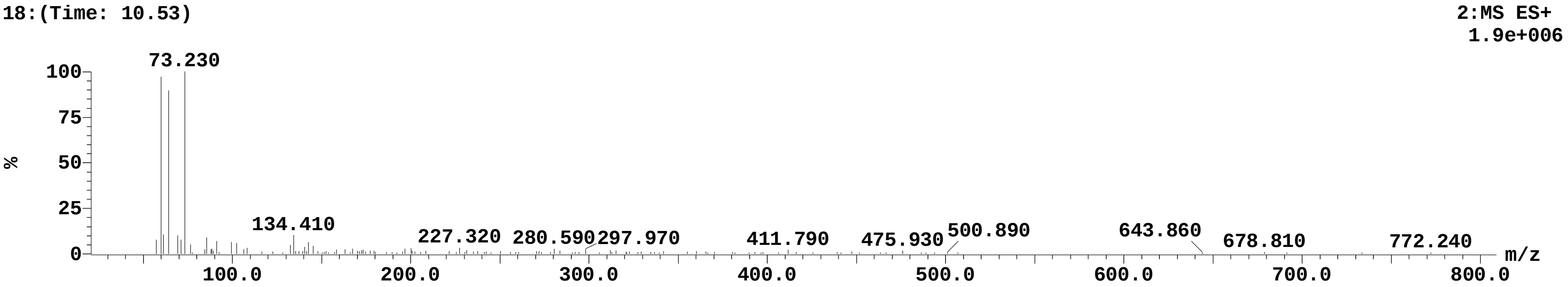

Sample: 644 Vial:1:B,5 ID:
File:LK-3 Date:03-Aug-2023 Time:13:20:09
Description:Default file

Printed: Thu Aug 03 15:05:46 2023

Sample Report (continued):

Peak ID	Compound	Time	Mass Found
19		10.78	

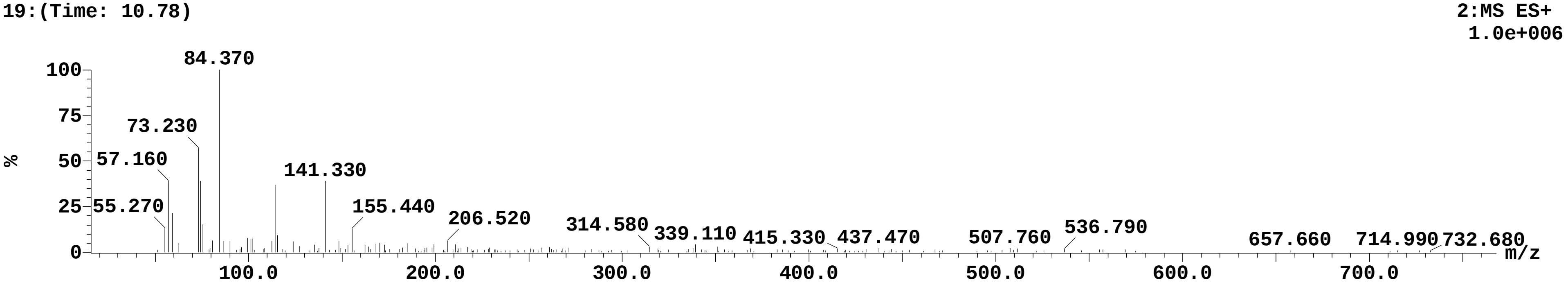

Peak ID	Compound	Time	Mass Found
20		11.18	

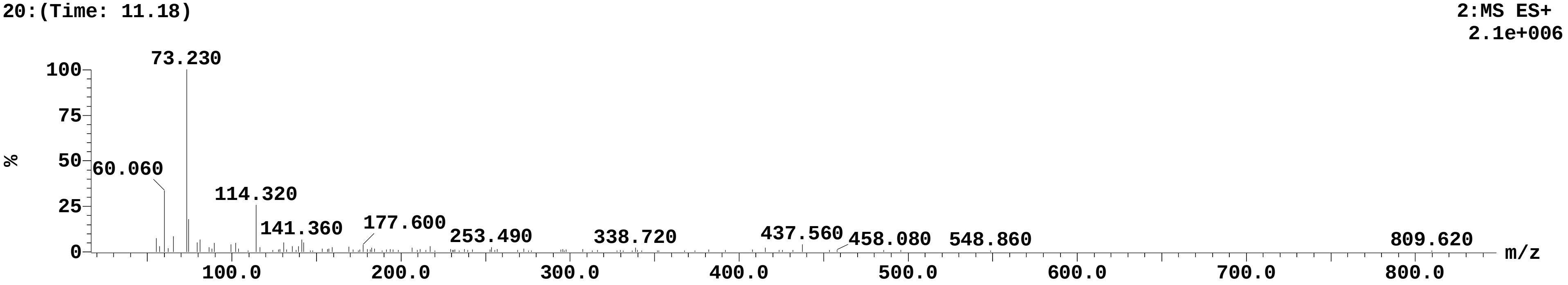

Sample: 644 Vial:1:B,5 ID:
File:LK-3 Date:03-Aug-2023 Time:13:20:09
Description:Default file

Printed: Thu Aug 03 15:05:46 2023

Sample Report (continued):

Peak ID	Compound	Time	Mass Found
21		11.32	

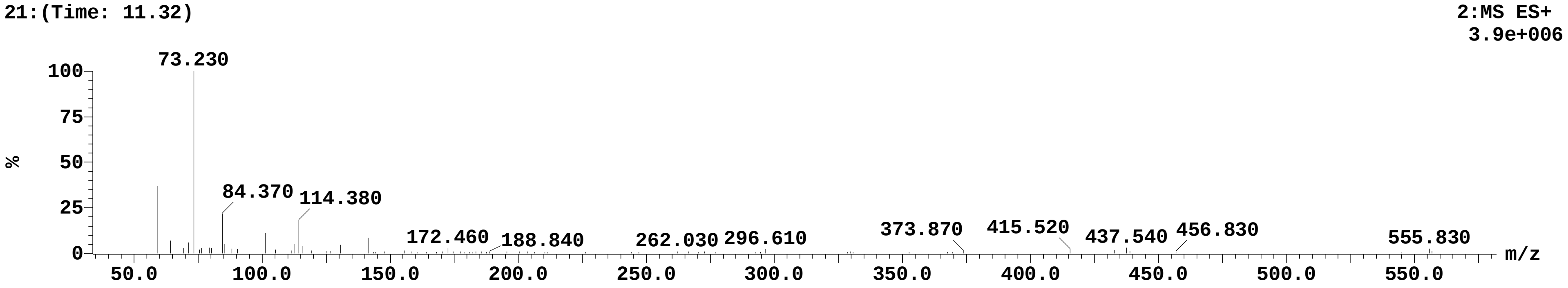

Peak ID	Compound	Time	Mass Found
22		12.07	

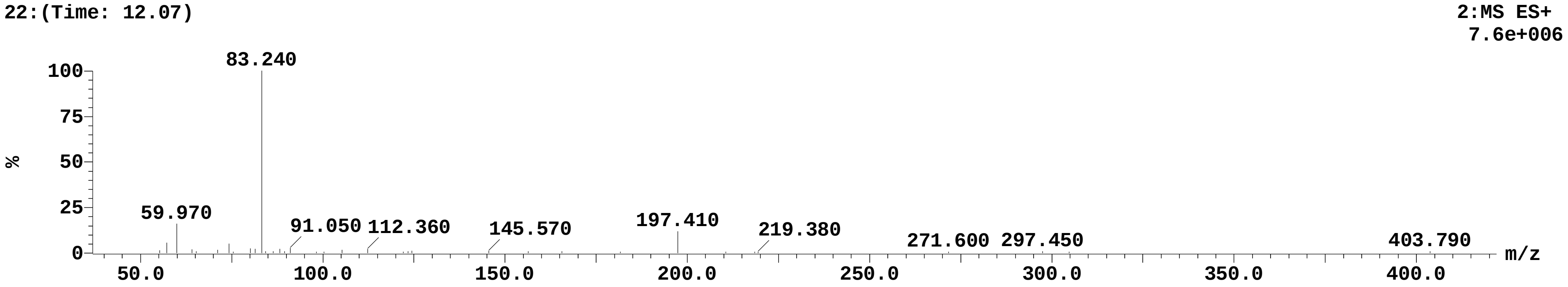

Sample: 644 Vial:1:B,5 ID:
File:LK-3 Date:03-Aug-2023 Time:13:20:09
Description:Default file

Printed: Thu Aug 03 15:05:46 2023

Sample Report (continued):

Peak ID	Compound	Time	Mass Found
23		12.08	

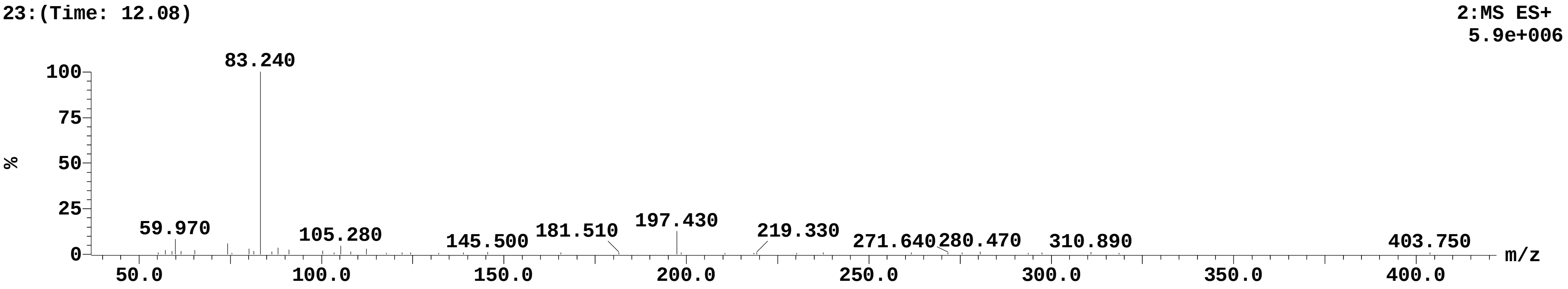

Peak ID	Compound	Time	Mass Found
24		13.07	

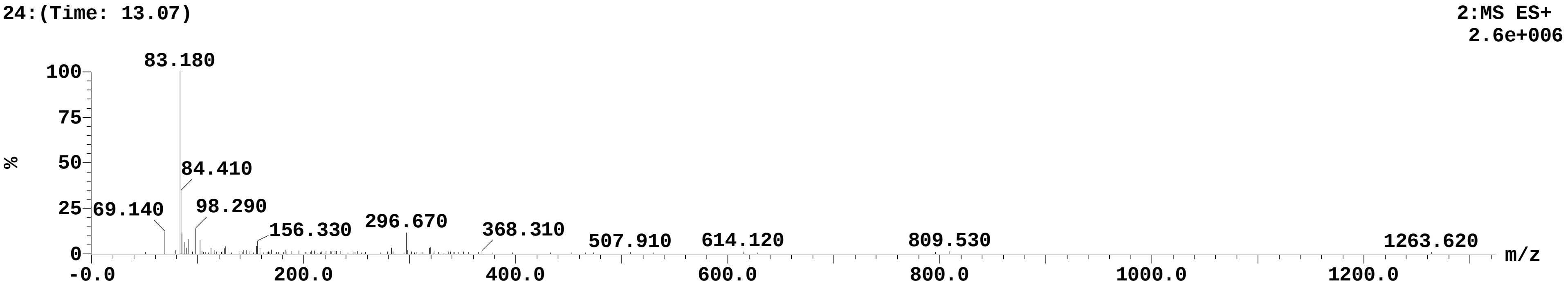

Sample Report (continued):

Peak ID	Compound	Time	Mass Found
25		13.13	

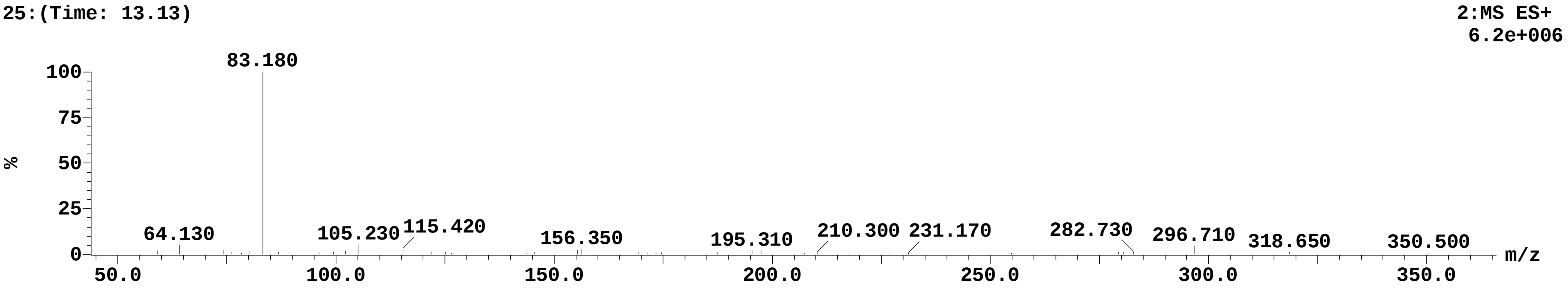

Peak ID	Compound	Time	Mass Found
26		14.30	

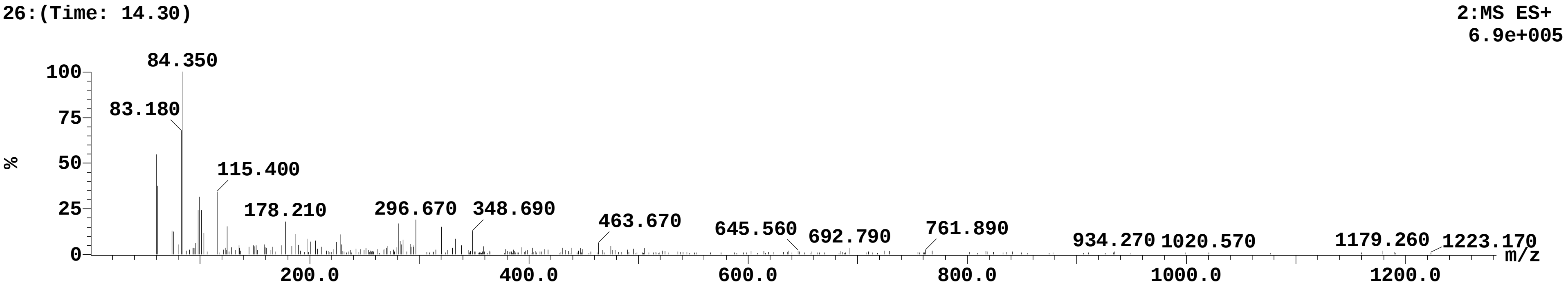

Sample: 644 Vial:1:B,5 ID:
File:LK-3 Date:03-Aug-2023 Time:13:20:09
Description:Default file

Printed: Thu Aug 03 15:05:46 2023

Sample Report (continued):

Peak ID	Compound	Time	Mass Found
27		14.68	

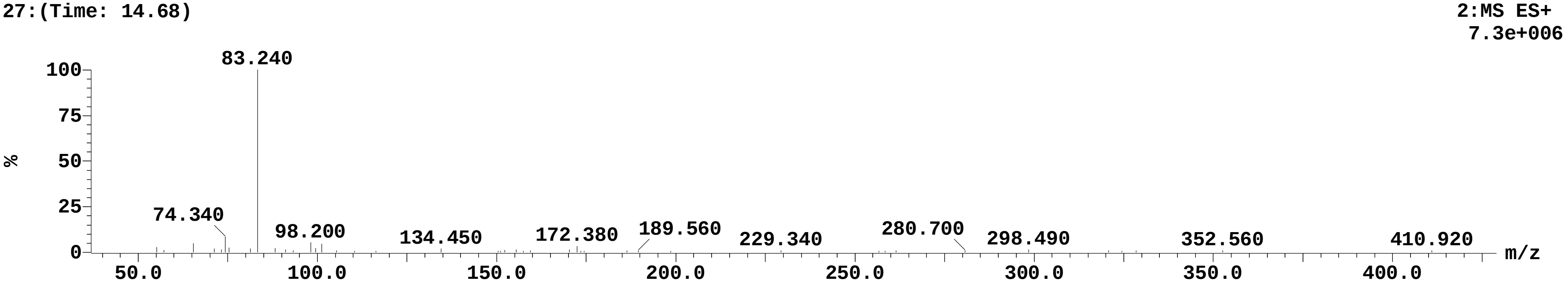

Peak ID	Compound	Time	Mass Found
28		15.80	

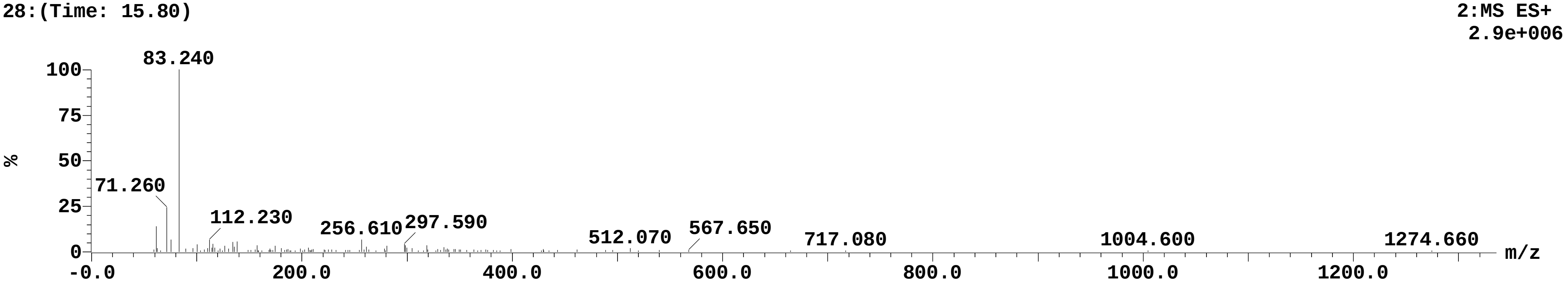

Sample: 644 Vial:1:B,5 ID:
File:LK-3 Date:03-Aug-2023 Time:13:20:09
Description:Default file

Printed: Thu Aug 03 15:05:46 2023

Sample Report (continued):

Peak ID	Compound	Time	Mass Found
29		16.28	

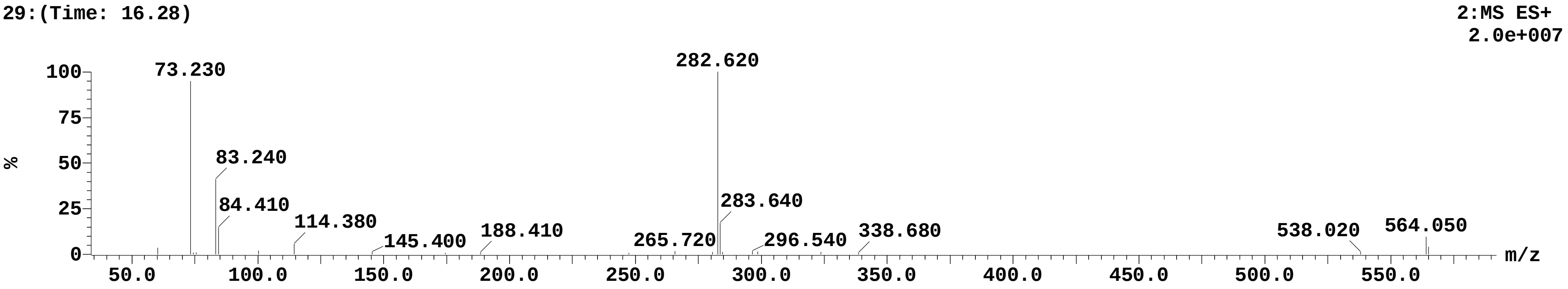

Peak ID	Compound	Time	Mass Found
30		16.58	

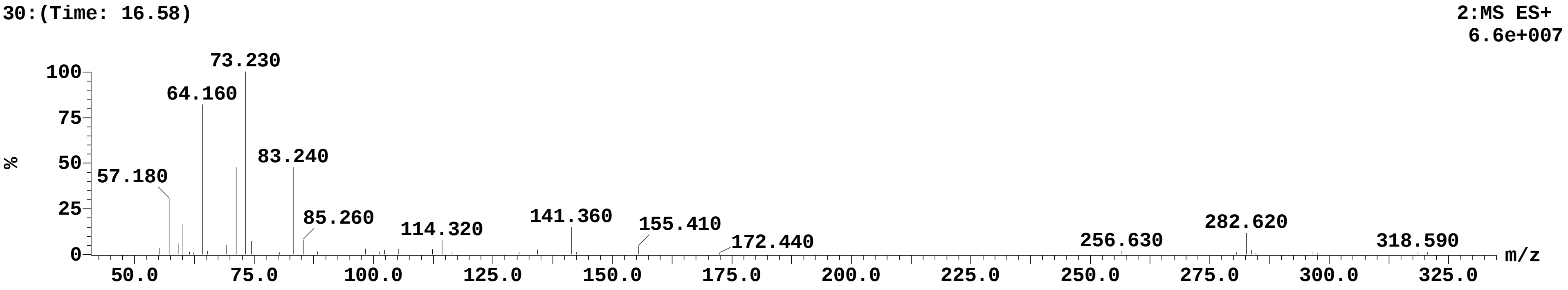

Sample Report (continued):

Peak ID	Compound	Time	Mass Found
31		16.88	

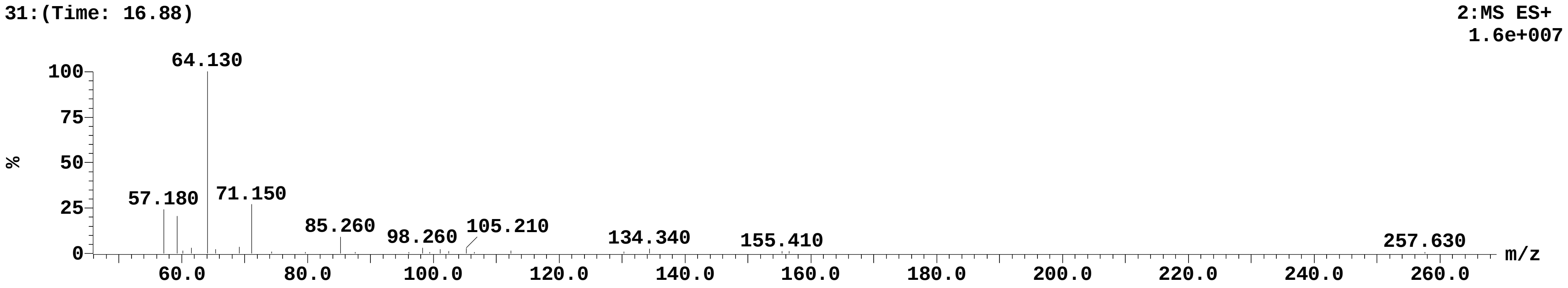

Peak ID	Compound	Time	Mass Found
32		17.27	

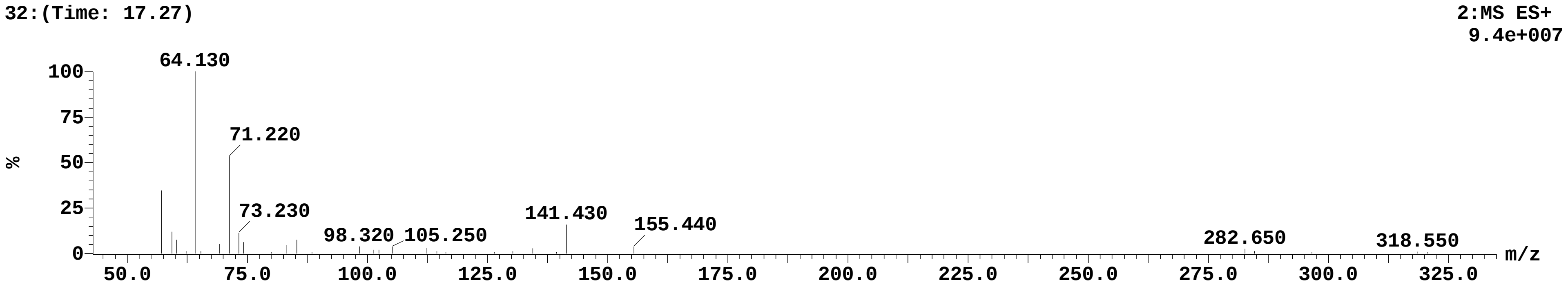

Sample: 644 Vial:1:B,5 ID:
File:LK-3 Date:03-Aug-2023 Time:13:20:09
Description:Default file

Printed: Thu Aug 03 15:05:46 2023

Sample Report (continued):

Peak ID	Compound	Time	Mass Found
33		17.40	

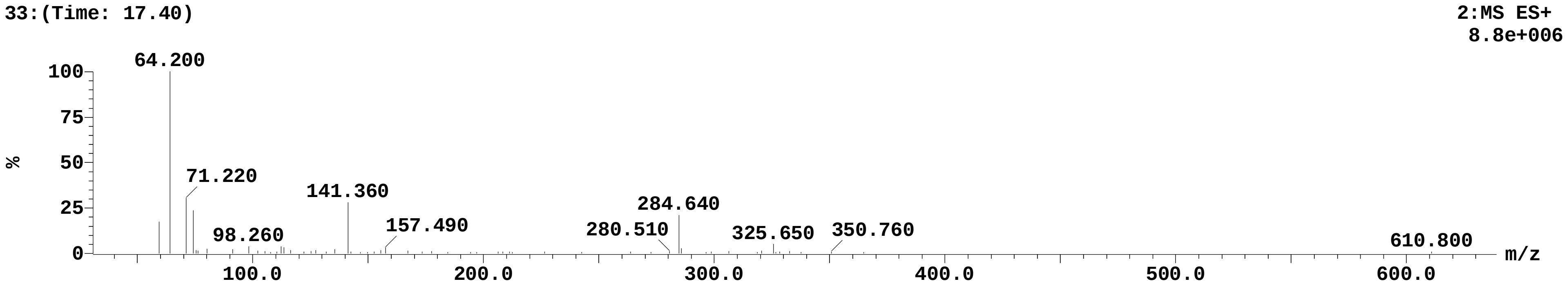

Supplement: Supplementary file 1 [file cimb-46-00230-s001.zip › cimb-2927003-supplementary.pdf]
